# Genome-Wide Association Study in *BRCA1* Mutation Carriers Identifies Novel Loci Associated with Breast and Ovarian Cancer Risk

**DOI:** 10.1371/journal.pgen.1003212

**Published:** 2013-03-27

**Authors:** Fergus J. Couch, Xianshu Wang, Lesley McGuffog, Andrew Lee, Curtis Olswold, Karoline B. Kuchenbaecker, Penny Soucy, Zachary Fredericksen, Daniel Barrowdale, Joe Dennis, Mia M. Gaudet, Ed Dicks, Matthew Kosel, Sue Healey, Olga M. Sinilnikova, Adam Lee, François Bacot, Daniel Vincent, Frans B. L. Hogervorst, Susan Peock, Dominique Stoppa-Lyonnet, Anna Jakubowska, kConFab Investigators, Paolo Radice, Rita Katharina Schmutzler, SWE-BRCA, Susan M. Domchek, Marion Piedmonte, Christian F. Singer, Eitan Friedman, Mads Thomassen, Ontario Cancer Genetics Network, Thomas V. O. Hansen, Susan L. Neuhausen, Csilla I. Szabo, Ignacio Blanco, Mark H. Greene, Beth Y. Karlan, Judy Garber, Catherine M. Phelan, Jeffrey N. Weitzel, Marco Montagna, Edith Olah, Irene L. Andrulis, Andrew K. Godwin, Drakoulis Yannoukakos, David E. Goldgar, Trinidad Caldes, Heli Nevanlinna, Ana Osorio, Mary Beth Terry, Mary B. Daly, Elizabeth J. van Rensburg, Ute Hamann, Susan J. Ramus, Amanda Ewart Toland, Maria A. Caligo, Olufunmilayo I. Olopade, Nadine Tung, Kathleen Claes, Mary S. Beattie, Melissa C. Southey, Evgeny N. Imyanitov, Marc Tischkowitz, Ramunas Janavicius, Esther M. John, Ava Kwong, Orland Diez, Judith Balmaña, Rosa B. Barkardottir, Banu K. Arun, Gad Rennert, Soo-Hwang Teo, Patricia A. Ganz, Ian Campbell, Annemarie H. van der Hout, Carolien H. M. van Deurzen, Caroline Seynaeve, Encarna B. Gómez Garcia, Flora E. van Leeuwen, Hanne E. J. Meijers-Heijboer, Johannes J. P. Gille, Margreet G. E. M. Ausems, Marinus J. Blok, Marjolijn J. L. Ligtenberg, Matti A. Rookus, Peter Devilee, Senno Verhoef, Theo A. M. van Os, Juul T. Wijnen, HEBON, EMBRACE, Debra Frost, Steve Ellis, Elena Fineberg, Radka Platte, D. Gareth Evans, Louise Izatt, Rosalind A. Eeles, Julian Adlard, Diana M. Eccles, Jackie Cook, Carole Brewer, Fiona Douglas, Shirley Hodgson, Patrick J. Morrison, Lucy E. Side, Alan Donaldson, Catherine Houghton, Mark T. Rogers, Huw Dorkins, Jacqueline Eason, Helen Gregory, Emma McCann, Alex Murray, Alain Calender, Agnès Hardouin, Pascaline Berthet, Capucine Delnatte, Catherine Nogues, Christine Lasset, Claude Houdayer, Dominique Leroux, Etienne Rouleau, Fabienne Prieur, Francesca Damiola, Hagay Sobol, Isabelle Coupier, Laurence Venat-Bouvet, Laurent Castera, Marion Gauthier-Villars, Mélanie Léoné, Pascal Pujol, Sylvie Mazoyer, Yves-Jean Bignon, GEMO Study Collaborators, Elżbieta Złowocka-Perłowska, Jacek Gronwald, Jan Lubinski, Katarzyna Durda, Katarzyna Jaworska, Tomasz Huzarski, Amanda B. Spurdle, Alessandra Viel, Bernard Peissel, Bernardo Bonanni, Giulia Melloni, Laura Ottini, Laura Papi, Liliana Varesco, Maria Grazia Tibiletti, Paolo Peterlongo, Sara Volorio, Siranoush Manoukian, Valeria Pensotti, Norbert Arnold, Christoph Engel, Helmut Deissler, Dorothea Gadzicki, Andrea Gehrig, Karin Kast, Kerstin Rhiem, Alfons Meindl, Dieter Niederacher, Nina Ditsch, Hansjoerg Plendl, Sabine Preisler-Adams, Stefanie Engert, Christian Sutter, Raymonda Varon-Mateeva, Barbara Wappenschmidt, Bernhard H. F. Weber, Brita Arver, Marie Stenmark-Askmalm, Niklas Loman, Richard Rosenquist, Zakaria Einbeigi, Katherine L. Nathanson, Timothy R. Rebbeck, Stephanie V. Blank, David E. Cohn, Gustavo C. Rodriguez, Laurie Small, Michael Friedlander, Victoria L. Bae-Jump, Anneliese Fink-Retter, Christine Rappaport, Daphne Gschwantler-Kaulich, Georg Pfeiler, Muy-Kheng Tea, Noralane M. Lindor, Bella Kaufman, Shani Shimon Paluch, Yael Laitman, Anne-Bine Skytte, Anne-Marie Gerdes, Inge Sokilde Pedersen, Sanne Traasdahl Moeller, Torben A. Kruse, Uffe Birk Jensen, Joseph Vijai, Kara Sarrel, Mark Robson, Noah Kauff, Anna Marie Mulligan, Gord Glendon, Hilmi Ozcelik, Bent Ejlertsen, Finn C. Nielsen, Lars Jønson, Mette K. Andersen, Yuan Chun Ding, Linda Steele, Lenka Foretova, Alex Teulé, Conxi Lazaro, Joan Brunet, Miquel Angel Pujana, Phuong L. Mai, Jennifer T. Loud, Christine Walsh, Jenny Lester, Sandra Orsulic, Steven A. Narod, Josef Herzog, Sharon R. Sand, Silvia Tognazzo, Simona Agata, Tibor Vaszko, Joellen Weaver, Alexandra V. Stavropoulou, Saundra S. Buys, Atocha Romero, Miguel de la Hoya, Kristiina Aittomäki, Taru A. Muranen, Mercedes Duran, Wendy K. Chung, Adriana Lasa, Cecilia M. Dorfling, Alexander Miron, BCFR, Javier Benitez, Leigha Senter, Dezheng Huo, Salina B. Chan, Anna P. Sokolenko, Jocelyne Chiquette, Laima Tihomirova, Tara M. Friebel, Bjarni A. Agnarsson, Karen H. Lu, Flavio Lejbkowicz, Paul A. James, Per Hall, Alison M. Dunning, Daniel Tessier, Julie Cunningham, Susan L. Slager, Chen Wang, Steven Hart, Kristen Stevens, Jacques Simard, Tomi Pastinen, Vernon S. Pankratz, Kenneth Offit, Douglas F. Easton, Georgia Chenevix-Trench, Antonis C. Antoniou, on behalf of CIMBA

**Affiliations:** 1 Department of Laboratory Medicine and Pathology, and Health Sciences Research, Mayo Clinic, Rochester, Minnesota, United States of America; 2 Department of Laboratory Medicine and Pathology, Mayo Clinic, Rochester, Minnesota, United States of America; 3 Centre for Cancer Genetic Epidemiology, Department of Public Health and Primary Care, University of Cambridge, Cambridge, United Kingdom; 4 Department of Health Sciences Research, Mayo Clinic, Rochester, Minnesota, United States of America; 5 Cancer Genomics Laboratory, Centre Hospitalier Universitaire de Québec and Laval University, Québec City, Canada; 6 Epidemiology Research Program, American Cancer Society, Atlanta, Georgia, United States of America; 7 Genetics Department, Queensland Institute of Medical Research, Brisbane, Australia; 8 Unité Mixte de Génétique Constitutionnelle des Cancers Fréquents, Hospices Civils de Lyon–Centre Léon Bérard, Lyon, France; 9 INSERM U1052, CNRS UMR5286, Université Lyon 1, Centre de Recherche en Cancérologie de Lyon, Lyon, France; 10 Department of Molecular Pharmacology and Experimental Therapeutics (MPET), Mayo Clinic, Rochester, Minnesota, United States of America; 11 Centre d'Innovation Génome Québec et Université McGill, Montreal, Canada; 12 Family Cancer Clinic, Netherlands Cancer Institute, Amsterdam, The Netherlands; 13 Institut Curie, Department of Tumour Biology, Paris, France; 14 Institut Curie, INSERM U830, Paris, France; 15 Université Paris Descartes, Sorbonne Paris Cité, Paris, France; 16 Department of Genetics and Pathology, Pomeranian Medical University, Szczecin, Poland; 17 Kathleen Cuningham Consortium for Research into Familial Breast Cancer–Peter MacCallum Cancer Center, Melbourne, Australia; 18 Unit of Molecular Bases of Genetic Risk and Genetic Testing, Department of Preventive and Predictive Medicine, Fondazione IRCCS Istituto Nazionale Tumori (INT), Milan, Italy; 19 IFOM, Fondazione Istituto FIRC di Oncologia Molecolare, Milan, Italy; 20 Centre of Familial Breast and Ovarian Cancer, Department of Gynaecology and Obstetrics and Centre for Integrated Oncology (CIO), Center for Molecular Medicine Cologne (CMMC), University Hospital of Cologne, Cologne, Germany; 21 Department of Oncology, Lund University, Lund, Sweden; 22 Abramson Cancer Center, University of Pennsylvania, Philadelphia, Pennsylvania, United States of America; 23 Gynecologic Oncology Group Statistical and Data Center, Roswell Park Cancer Institute, Buffalo, New York, United States of America; 24 Department of Obstetrics and Gynecology, and Comprehensive Cancer Center, Medical University of Vienna, Vienna, Austria; 25 Sheba Medical Center, Tel Aviv, Israel; 26 Department of Clinical Genetics, Odense University Hospital, Odense, Denmark; 27 Samuel Lunenfeld Research Institute, Mount Sinai Hospital, Toronto, Canada; 28 Center for Genomic Medicine, Rigshospitalet, Copenhagen University Hospital, Copenhagen, Denmark; 29 Department of Population Sciences, Beckman Research Institute of City of Hope, Duarte, California, United States of America; 30 Center for Translational Cancer Research, Department of Biological Sciences, University of Delaware, Newark, Delaware, United States of America; 31 Genetic Counseling Unit, Hereditary Cancer Program, IDIBELL–Catalan Institute of Oncology, Barcelona, Spain; 32 Clinical Genetics Branch, Division of Cancer Epidemiology and Genetics, National Cancer Institute, National Institutes of Health, Rockville, Maryland, United States of America; 33 Women's Cancer Program at the Samuel Oschin Comprehensive Cancer Institute, Cedars-Sinai Medical Center, Los Angeles, California, United States of America; 34 Dana-Farber Cancer Institute, Boston, Massachusetts, United States of America; 35 Department of Cancer Epidemiology, Moffitt Cancer Center, Tampa, Florida, United States of America; 36 Clinical Cancer Genetics (for the City of Hope Clinical Cancer Genetics Community Research Network), City of Hope, Duarte, California, United States of America; 37 Immunology and Molecular Oncology Unit, Istituto Oncologico Veneto IOV–IRCCS, Padua, Italy; 38 Department of Molecular Genetics, National Institute of Oncology, Budapest, Hungary; 39 Samuel Lunenfeld Research Institute, Mount Sinai Hospital, Toronto, Ontario, Canada; 40 Department of Pathology and Laboratory Medicine, University of Kansas Medical Center, Kansas City, Kansas, United States of America; 41 Molecular Diagnostics Laboratory, IRRP, National Centre for Scientific Research Demokritos, Aghia Paraskevi Attikis, Athens, Greece; 42 Department of Dermatology, University of Utah School of Medicine, Salt Lake City, Utah, United States of America; 43 Molecular Oncology Laboratory, Hospital Clinico San Carlos, IdISSC, Madrid, Spain; 44 Department of Obstetrics and Gynecology, University of Helsinki and Helsinki University Central Hospital, Helsinki, Finland; 45 Human Genetics Group, Spanish National Cancer Centre (CNIO), and Biomedical Network on Rare Diseases (CIBERER), Madrid, Spain; 46 Department of Epidemiology, Columbia University, New York, New York, United States of America; 47 Fox Chase Cancer Center, Philadelphia, Pennsylvania, United States of America; 48 Department of Genetics, University of Pretoria, Pretoria, South Africa; 49 Molecular Genetics of Breast Cancer, Deutsches Krebsforschungszentrum (DKFZ), Heidelberg, Germany; 50 Department of Preventive Medicine, Keck School of Medicine, University of Southern California, California, United States of America; 51 Divison of Human Cancer Genetics, Departments of Internal Medicine and Molecular Virology, Immunology and Medical Genetics, Comprehensive Cancer Center, The Ohio State University, Columbus, Ohio, United States of America; 52 Section of Genetic Oncology, Department of Laboratory Medicine, University of Pisa and University Hospital of Pisa, Pisa, Italy; 53 Center for Clinical Cancer Genetics and Global Health, University of Chicago Medical Center, Chicago, Illinois, United States of America; 54 Department of Medical Oncology, Beth Israel Deaconess Medical Center, Boston, Massachusetts, United States of America; 55 Center for Medical Genetics, Ghent University Hospital, Ghent, Belgium; 56 Departments of Medicine, Epidemiology, and Biostatistics, University of California San Francisco, San Francisco, California, United States of America; 57 Genetic Epidemiology Laboratory, Department of Pathology, University of Melbourne, Parkville, Australia; 58 N. N. Petrov Institute of Oncology, St. Petersburg, Russia; 59 Program in Cancer Genetics, Departments of Human Genetics and Oncology, McGill University, Montreal, Quebec, Canada; 60 Vilnius University Hospital Santariskiu Clinics, Hematology, Oncology and Transfusion Medicine Center, Department of Molecular and Regenerative Medicine, Vilnius, Lithuania; 61 Department of Epidemiology, Cancer Prevention Institute of California, Fremont, Califoria, United States of America; 62 The Hong Kong Hereditary Breast Cancer Family Registry, Cancer Genetics Center, Hong Kong Sanatorium and Hospital, Hong Kong, China; 63 Oncogenetics Laboratory, University Hospital Vall d'Hebron and Vall d'Hebron Institute of Oncology (VHIO), Barcelona, Spain; 64 Department of Medical Oncology, University Hospital, Vall d'Hebron, Barcelona, Spain; 65 Department of Pathology, Landspitali University Hospital and BMC, Faculty of Medicine, University of Iceland, Reykjavik, Iceland; 66 Department of Breast Medical Oncology and Clinical Cancer Genetics, University of Texas MD Anderson Cancer Center, Houston, Texas, United States of America; 67 Clalit National Israeli Cancer Control Center and Department of Community Medicine and Epidemiology, Carmel Medical Center and B. Rappaport Faculty of Medicine, Haifa, Israel; 68 Cancer Research Initiatives Foundation, Sime Darby Medical Centre and University Malaya Cancer Research Institute, University of Malaya, Kuala Lumpur, Malaysia; 69 UCLA Schools of Medicine and Public Health, Division of Cancer Prevention and Control Research, Jonsson Comprehensive Cancer Center, Los Angeles, California, United States of America; 70 VBCRC Cancer Genetics Laboratory, Peter MacCallum Cancer Centre, Melbourne, Australia; 71 Department of Genetics, University Medical Center, Groningen University, Groningen, The Netherlands; 72 Department of Pathology, Family Cancer Clinic, Erasmus University Medical Center, Rotterdam, The Netherlands; 73 Department of Medical Oncology, Family Cancer Clinic, Erasmus University Medical Center, Rotterdam, The Netherlands; 74 Department of Clinical Genetics and GROW, School for Oncology and Developmental Biology, MUMC, Maastricht, The Netherlands; 75 Department of Epidemiology, Netherlands Cancer Institute, Amsterdam, The Netherlands; 76 Department of Clinical Genetics, VU University Medical Centre, Amsterdam, The Netherlands; 77 Department of Medical Genetics, University Medical Center Utrecht, Utrecht, The Netherlands; 78 Department of Clinical Genetics, Maastricht University Medical Center, Maastricht, The Netherlands; 79 Department of Human Genetics and Department of Pathology, Radboud University Nijmegen Medical Centre, Nijmegen, The Netherlands; 80 Department of Human Genetics and Department of Pathology, Leiden University Medical Center, Leiden, The Netherlands; 81 Department of Clinical Genetics, Academic Medical Center, Amsterdam, The Netherlands; 82 Department of Human Genetics and Department of Clinical Genetics, Leiden University Medical Center, Leiden, The Netherlands; 83 The Hereditary Breast and Ovarian Cancer Research Group Netherlands, Netherlands Cancer Institute, Amsterdam, The Netherlands; 84 Genetic Medicine, Manchester Academic Health Sciences Centre, Central Manchester University Hospitals NHS Foundation Trust, Manchester, United Kingdom; 85 Clinical Genetics, Guy's and St. Thomas' NHS Foundation Trust, London, United Kingdom; 86 Oncogenetics Team, The Institute of Cancer Research and Royal Marsden NHS Foundation Trust, London, United Kingdom; 87 Yorkshire Regional Genetics Service, Leeds, United Kingdom; 88 University of Southampton Faculty of Medicine, Southampton University Hospitals NHS Trust, Southampton, United Kingdom; 89 Sheffield Clinical Genetics Service, Sheffield Children's Hospital, Sheffield, United Kingdom; 90 Department of Clinical Genetics, Royal Devon and Exeter Hospital, Exeter, United Kingdom; 91 Institute of Genetic Medicine, Centre for Life, Newcastle Upon Tyne Hospitals NHS Trust, Newcastle upon Tyne, United Kingdom; 92 Department of Clinical Genetics, St George's University of London, London, United Kingdom; 93 Northern Ireland Regional Genetics Centre, Belfast Health and Social Care Trust, and Department of Medical Genetics, Queens University Belfast, Belfast, United Kingdom; 94 North East Thames Regional Genetics Service, Great Ormond Street Hospital for Children NHS Trust and Institute for Womens Health, University College London, London, United Kingdom; 95 Clinical Genetics Department, St Michael's Hospital, Bristol, United Kingdom; 96 Cheshire and Merseyside Clinical Genetics Service, Liverpool Women's NHS Foundation Trust, Liverpool, United Kingdom; 97 All Wales Medical Genetics Services, University Hospital of Wales, Cardiff, United Kingdom; 98 North West Thames Regional Genetics Service, Kennedy-Galton Centre, Harrow, United Kingdom; 99 Nottingham Clinical Genetics Service, Nottingham University Hospitals NHS Trust, Nottingham, United Kingdom; 100 North of Scotland Regional Genetics Service, NHS Grampian and University of Aberdeen, Foresterhill, Aberdeen, United Kingdom; 101 All Wales Medical Genetics Services, Glan Clwyd Hospital, Rhyl, United Kingdom; 102 All Wales Medical Genetics Services, Singleton Hospital, Swansea, United Kingdom; 103 Centre François Baclesse, Caen, France; 104 Centre René Gauducheau, Nantes, France; 105 Oncogénétique Clinique, Hôpital René Huguenin/Institut Curie, Saint-Cloud, France; 106 Unité de Prévention et d'Epidémiologie Génétique, Centre Léon Bérard, Lyon, France; 107 Université Lyon 1, CNRS UMR5558, Lyon, France; 108 Department of Genetics, Centre Hospitalier Universitaire de Grenoble, Grenoble, France; 109 Institut Albert Bonniot, Université de Grenoble, Grenoble, France; 110 Laboratoire d'Oncogénétique, Hôpital René Huguenin, Institut Curie, Saint-Cloud, France; 111 Service de Génétique Clinique Chromosomique et Moléculaire, Centre Hospitalier Universitaire de St Etienne, St Etienne, France; 112 Département Oncologie Génétique, Prévention et Dépistage, INSERM CIC-P9502, Institut Paoli-Calmettes/Université d'Aix-Marseille II, Marseille, France; 113 Unité d'Oncogénétique, CHU Arnaud de Villeneuve, Montpellier, France; 114 Unité d'Oncogénétique, CRLCC Val d'Aurelle, Montpellier, France; 115 Department of Medical Oncology, Centre Hospitalier Universitaire Dupuytren, Limoges, France; 116 INSERM 896, CRCM Val d'Aurelle, Montpellier, France; 117 Département d'Oncogénétique, Centre Jean Perrin, Université de Clermont-Ferrand, Clermont-Ferrand, France; 118 National Cancer Genetics Network, UNICANCER Genetic Group, Centre de Recherche en Cancérologie de Lyon and Institut Curie Paris, Paris, France; 119 Postgraduate School of Molecular Medicine, Warsaw Medical University, Warsaw, Poland; 120 Division of Experimental Oncology 1, Centro di Riferimento Oncologico, IRCCS, Aviano, Italy; 121 Unit of Medical Genetics, Department of Preventive and Predictive Medicine, Fondazione IRCCS Istituto Nazionale Tumori (INT), Milan, Italy; 122 Division of Cancer Prevention and Genetics, Istituto Europeo di Oncologia, Milan, Italy; 123 Department of Molecular Medicine, Sapienza University, Rome, Italy; 124 Unit of Medical Genetics, Department of Clinical Physiopathology, University of Florence, Firenze, Italy; 125 Unit of Hereditary Cancer, Department of Epidemiology, Prevention and Special Functions, IRCCS AOU San Martino–IST Istituto Nazionale per la Ricerca sul Cancro, Genoa, Italy; 126 UO Anatomia Patologica, Ospedale di Circolo-Università dell'Insubria, Varese, Italy; 127 IFOM, Fondazione Istituto FIRC di Oncologia Molecolare and Cogentech Cancer Genetic Test Laboratory, Milan, Italy; 128 University Hospital of Schleswig-Holstein/University Kiel, Kiel, Germany; 129 Institute for Medical Informatics, Statistics and Epidemiology, University of Leipzig, Leipzig, Germany; 130 University Hospital Ulm, Ulm, Germany; 131 Hannover Medical School, Hanover, Germany; 132 Institute of Human Genetics, University of Würzburg, Wurzburg, Germany; 133 Department of Gynaecology and Obstetrics, University Hospital Carl Gustav Carus, Technical University Dresden, Dresden, Germany; 134 Department of Gynaecology and Obstetrics, Division of Tumor Genetics, Klinikum rechts der Isar, Technical University of Munich, Munich, Germany; 135 Department of Obstetrics and Gynaecology, University Medical Center Düsseldorf, Heinrich-Heine-University, Düsseldorf, Germany; 136 Department of Gynaecology and Obstetrics, University of Munich, Munich, Germany; 137 Institute of Human Genetics, University Hospital of Schleswig-Holstein, University of Kiel, Kiel, Germany; 138 Institute of Human Genetics, Münster, Germany; 139 Institute of Human Genetics, University of Heidelberg, Heidelberg, Germany; 140 Institute of Medical and Human Genetics, Berlin, Germany; 141 Institute of Human Genetics, University of Regensburg, Regensburg, Germany; 142 Department of Oncology and Pathology, Karolinska University Hospital, Stockholm, Sweden; 143 Division of Clinical Genetics, Department of Clinical and Experimental Medicine, Linköping University, Linköping, Sweden; 144 Department of Oncology, Lund University Hospital, Lund, Sweden; 145 Department of Immunology, Genetics and Pathology, Rudbeck Laboratory, Uppsala University, Uppsala, Sweden; 146 Department of Oncology, Sahlgrenska University Hospital, Gothenburg, Sweden; 147 Abramson Cancer Center and Department of Medicine, Perelman School of Medicine, University of Pennsylvania, Philadelphia, Pennsylvania, United States of America; 148 Abramson Cancer Center and Center for Clinical Epidemiology and Biostatistics, Perelman School of Medicine, University of Pennsylvania, Philadelphia, Pennsylvania, United States of America; 149 NYU Women's Cancer Program, New York University School of Medicine, New York, New York, United States of America; 150 Ohio State University, Columbus Cancer Council, Columbus, Ohio, United States of America; 151 Division of Gynecologic Oncology, North Shore University Health System, University of Chicago, Evanston, Illinois, United States of America; 152 Maine Medical Center, Maine Women's Surgery and Cancer Centre, Scarborough, Maine, United States of America; 153 ANZ GOTG Coordinating Centre, Australia New Zealand GOG, Camperdown, Australia; 154 The University of North Carolina at Chapel Hill, Chapel Hill, North Carolina, United States of America; 155 Department of Health Science Research, Mayo Clinic Arizona, Scottsdale, Arizona, United States of America; 156 Department of Clinical Genetics, Vejle Hospital, Vejle, Denmark; 157 Department of Clincial Genetics, Rigshospitalet, København, Denmark; 158 Section of Molecular Diagnostics, Department of Clinical Biochemistry, Aalborg University Hospital, Aalborg, Denmark; 159 Department of Clinical Genetics, Aarhus University Hospital, Aarhus, Denmark; 160 Clinical Genetics Service, Memorial Sloan-Kettering Cancer Center, New York, New York, United States of America; 161 Department of Laboratory Medicine and Pathobiology, University of Toronto, Toronto, Canada; 162 Department of Laboratory Medicine, and the Keenan Research Centre of the Li Ka Shing Knowledge Institute, St Michael's Hospital, Toronto, Canada; 163 Department of Oncology, Rigshospitalet, Copenhagen University Hospital, Copenhagen, Denmark; 164 Department of Clinical Genetics, Rigshospitalet, Copenhagen University Hospital, Copenhagen, Denmark; 165 Department of Cancer Epidemiology and Genetics, Masaryk Memorial Cancer Institute, Brno, Czech Republic; 166 Molecular Diagnostic Unit, Hereditary Cancer Program, IDIBELL–Catalan Institute of Oncology, Barcelona, Spain; 167 Genetic Counseling Unit, Hereditary Cancer Program, IDIBGI–Catalan Institute of Oncology, Girona, Spain; 168 Translational Research Laboratory, Breast Cancer and Systems Biology Unit, IDIBELL–Catalan Institute of Oncology, Barcelona, Spain; 169 Women's College Research Institute, University of Toronto, Toronto, Canada; 170 Biosample Repository, Fox Chase Cancer Center, Philadelphia, Pennsylvania, United States of America; 171 Department of Internal Medicine, Huntsman Cancer Institute, University of Utah School of Medicine, Salt Lake City, Utah, United States of America; 172 Department of Clinical Genetics, Helsinki University Central Hospital, Helsinki, Finland; 173 Institute of Biology and Molecular Genetics, Universidad de Valladolid (IBGM–UVA), Valladolid, Spain; 174 Departments of Pediatrics and Medicine, Columbia University, New York, New York, United States of America; 175 Genetics Service, Hospital de la Santa Creu i Sant Pau, Barcelona, Spain; 176 Department of Cancer Biology, Dana-Farber Cancer Institute, Boston, Massachusetts, United States of America; 177 Breast Cancer Family Registry, Cancer Prevention Institute of California, Fremont, California, United States of America; 178 Human Genetics Group and Genotyping Unit, Spanish National Cancer Centre (CNIO), and Biomedical Network on Rare Diseases (CIBERER), Madrid, Spain; 179 Divison of Human Genetics, Department of Internal Medicine, The Comprehensive Cancer Center, The Ohio State University, Columbus, Ohio, United States of America; 180 Cancer Risk Program, Helen Diller Family Cancer Center, University of California San Francisco, San Francisco, California, United States of America; 181 Unité de Recherche en Santé des Populations, Centre des Maladies du Sein Deschênes-Fabia, Centre de Recherche FRSQ du Centre Hospitalier Affilié Universitaire de Québec, Québec, Canada; 182 Latvian Biomedical Research and Study Centre, Riga, Latvia; 183 University of Pennsylvania, Philadelphia, Pennsylvania, United States of America; 184 Landspitali University Hospital and University of Iceland School of Medicine, Reykjavik, Iceland; 185 Clalit National Israeli Cancer Control Center and Department of Community Medicine and Epidemiology, Carmel Medical Center, Haifa, Israel; 186 Familial Cancer Centre, Peter MacCallum Cancer Centre, Melbourne, Australia; 187 Department of Medical Epidemiology and Biostatistics, Karolinska Institutet, Stockholm, Sweden; 188 Centre for Cancer Genetic Epidemiology, Department of Oncology, University of Cambridge, Cambridge, United Kingdom; 189 Department of Human Genetics, McGill University and Génome Québec Innovation Centre, McGill University, Montréal, Canada; National Cancer Institute, United States of America

## Abstract

*BRCA1*-associated breast and ovarian cancer risks can be modified by common genetic variants. To identify further cancer risk-modifying loci, we performed a multi-stage GWAS of 11,705 *BRCA1* carriers (of whom 5,920 were diagnosed with breast and 1,839 were diagnosed with ovarian cancer), with a further replication in an additional sample of 2,646 *BRCA1* carriers. We identified a novel breast cancer risk modifier locus at 1q32 for *BRCA1* carriers (rs2290854, P = 2.7×10^−8^, HR = 1.14, 95% CI: 1.09–1.20). In addition, we identified two novel ovarian cancer risk modifier loci: 17q21.31 (rs17631303, P = 1.4×10^−8^, HR = 1.27, 95% CI: 1.17–1.38) and 4q32.3 (rs4691139, P = 3.4×10^−8^, HR = 1.20, 95% CI: 1.17–1.38). The 4q32.3 locus was not associated with ovarian cancer risk in the general population or *BRCA2* carriers, suggesting a *BRCA1*-specific association. The 17q21.31 locus was also associated with ovarian cancer risk in 8,211 *BRCA2* carriers (P = 2×10^−4^). These loci may lead to an improved understanding of the etiology of breast and ovarian tumors in *BRCA1* carriers. Based on the joint distribution of the known *BRCA1* breast cancer risk-modifying loci, we estimated that the breast cancer lifetime risks for the 5% of *BRCA1* carriers at lowest risk are 28%–50% compared to 81%–100% for the 5% at highest risk. Similarly, based on the known ovarian cancer risk-modifying loci, the 5% of *BRCA1* carriers at lowest risk have an estimated lifetime risk of developing ovarian cancer of 28% or lower, whereas the 5% at highest risk will have a risk of 63% or higher. Such differences in risk may have important implications for risk prediction and clinical management for *BRCA1* carriers.

## Author Summary

*BRCA1* mutation carriers have increased and variable risks of breast and ovarian cancer. To identify modifiers of breast and ovarian cancer risk in this population, a multi-stage GWAS of 14,351 *BRCA1* mutation carriers was performed. Loci 1q32 and *TCF7L2* at 10q25.3 were associated with breast cancer risk, and two loci at 4q32.2 and 17q21.31 were associated with ovarian cancer risk. The 4q32.3 ovarian cancer locus was not associated with ovarian cancer risk in the general population or in *BRCA2* carriers and is the first indication of a *BRCA1*-specific risk locus for either breast or ovarian cancer. Furthermore, modeling the influence of these modifiers on cumulative risk of breast and ovarian cancer in *BRCA1* mutation carriers for the first time showed that a wide range of individual absolute risks of each cancer can be estimated. These differences suggest that genetic risk modifiers may be incorporated into the clinical management of *BRCA1* mutation carriers.

## Introduction

Breast and ovarian cancer risk estimates for *BRCA1* mutation carriers vary by the degree of family history of the disease, suggesting that other genetic factors modify cancer risks for this population [1]–[4]. Studies by the Consortium of Investigators of Modifiers of *BRCA1/2* (CIMBA) have shown that a subset of common alleles influencing breast and ovarian cancer risk in the general population are also associated with cancer risk in *BRCA1* mutation carriers [5]–[11]. In particular, the breast cancer associations were limited to loci associated with estrogen receptor (ER)-negative breast cancer in the general population (6q25.1, 12p11 and *TOX3*) [8]–[11].

To systematically search for loci associated with breast or ovarian cancer risk for *BRCA1* carriers we previously conducted a two-stage genome-wide association study (GWAS) [12]. The initial stage involved analysis of 555,616 SNPs in 2383 *BRCA1* mutation carriers (1,193 unaffected and 1,190 affected). After replication testing of 89 SNPs showing the strongest association, with 5,986 *BRCA1* mutation carriers, a locus on 19p13 was shown to be associated with breast cancer risk for *BRCA1* mutation carriers. The same locus was also associated with the risk of estrogen-receptor (ER) negative and triple negative (ER, Progesterone and HER2 negative) breast cancer in the general population [12], [13].

The Collaborative Oncological Gene-environment Study (COGS) consortium recently developed a 211,155 SNP custom genotyping array (iCOGS) in order to provide cost-effective genotyping of common and rare genetic variants to identify novel loci that explain the residual genetic variance of breast, ovarian and prostate cancers and fine-map known susceptibility loci. A total of 32,557 SNPs on the iCOGS array were selected on the basis of the *BRCA1* GWAS for the purpose of identifying breast and ovarian cancer risk modifiers for *BRCA1* mutation carriers. Genotype data from the iCOGS array were obtained for 11,705 samples from *BRCA1* carriers and the 17 most promising SNPs were then genotyped in an additional 2,646 *BRCA1* carriers. In this manuscript we report on the novel risk modifier loci identified by this multi-stage GWAS. No study has previously shown how the absolute risks of breast and ovarian cancer for *BRCA1* mutation carriers vary by the combined effects of risk modifying loci. Here we use the results from this study, in combination with previously identified modifiers, to obtain absolute risks of developing breast and ovarian cancer for *BRCA1* mutation carriers based on the joint distribution of all known genetic risk modifiers.

## Materials and Methods

### Ethics statement

All carriers participated in clinical or research studies at the host institutions, approved by local ethics committees.

### Study subjects

*BRCA1* mutation carriers were recruited by 45 study centers in 25 countries through CIMBA. The majority were recruited through cancer genetics clinics, and enrolled into national or regional studies. The remainder were identified by population-based sampling or community recruitment. Eligibility for CIMBA association studies was restricted to female carriers of pathogenic *BRCA1* mutations age 18 years or older at recruitment. Information collected included year of birth, mutation description, self-reported ethnic ancestry, age at last follow-up, ages at breast or ovarian cancer diagnoses, and age at bilateral prophylactic mastectomy and oophorectomy. Information on tumour characteristics, including ER-status of the breast cancers, was also collected. Related individuals were identified through a unique family identifier. Women were included in the analysis if they carried mutations that were pathogenic according to generally recognized criteria.

#### GWAS stage 1 samples

**Figure 1:**
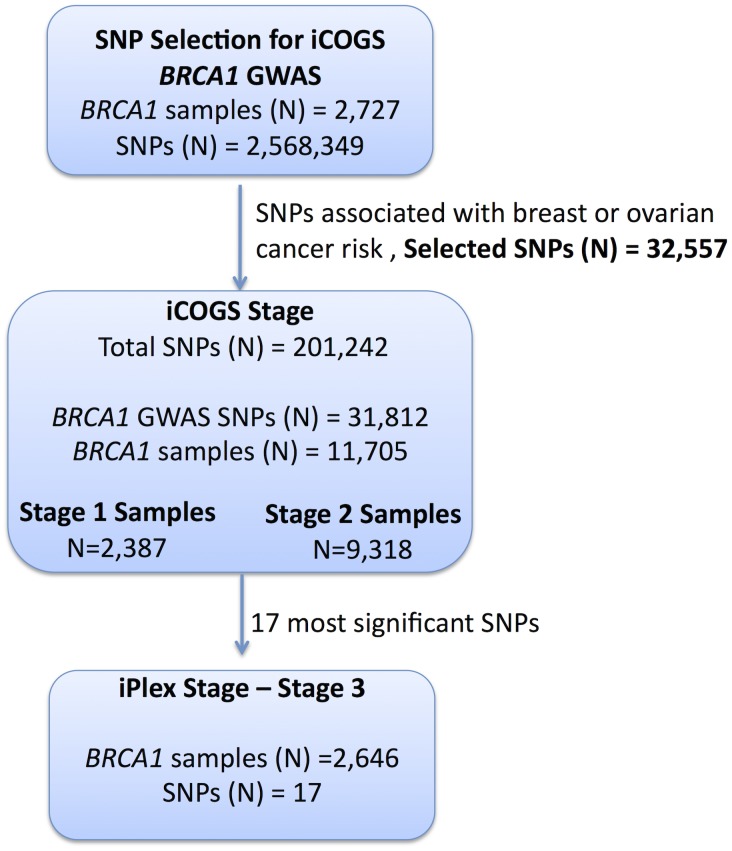
Study design for selection of the SNPs and genotyping of *BRCA1* samples. GWAS data from 2,727 *BRCA1* mutation carriers were analysed for associations with breast and ovarian cancer risk and 32,557 SNPs were selected for inclusion on the iCOGS array. A total of 11,705 *BRCA1* samples (after quality control (QC) checks) were genotyped on the 31,812 *BRCA1*-GWAS SNPs from the iCOGS array that passed QC. Of these samples, 2,387 had been genotyped at the SNP selection stage and are referred to as “stage 1” samples, whereas 9,318 samples were unique to the iCOGS study (“Stage 2” samples). Next, 17 SNPs that exhibited the most significant associations with breast and ovarian cancer were selected for genotyping in a third stage involving an additional 2,646 *BRCA1* samples (after QC).

A total of 2,727 *BRCA1* mutation carriers were genotyped on the Illumina Infinium 610K array (Figure 1). Of these 1,426 diagnosed with a first breast cancer under age 40 were considered “affected” in the breast cancer association analysis and 683 diagnosed with an ovarian cancer at any time were considered as “affected” in the ovarian cancer analysis. “Unaffected” in both analyses were over age 35 (Table S1) [12].

#### Replication study samples

All eligible *BRCA1* carriers from CIMBA with sufficient DNA were genotyped, including those used in Stage 1. In total, 13,310 samples from 45 centers in 25 countries were genotyped using the iCOGS array (Table S2). Among the 13,310 samples, those that were genotyped in the GWAS stage 1 SNP selection stage are referred to as “stage 1” samples, and the remainder are “stage 2” samples. An additional 2,646 *BRCA1* samples “stage 3” were genotyped on an iPLEX Mass Array of 17 SNPs from 12 loci selected after an interim analysis of iCOGS array data and were available for analysis after quality control (QC) (Figure 1). Carriers of pathogenic mutations in *BRCA2* were drawn from a parallel GWAS of genetic modifiers for *BRCA2* mutation carriers. *BRCA2* mutation carriers were recruited from CIMBA through 47 studies which were largely the same as the studies that contributed to the *BRCA1* GWAS with similar eligibility criteria. Samples from *BRCA2* mutation carriers were also genotyped using the iCOGS array. Details of this experiment are described elsewhere [14]. A total of 8,211 samples were available for analysis after QC.

### iCOGS SNP array

The iCOGS array was designed in a collaboration among the Breast Cancer Association Consortium (BCAC), Ovarian Cancer Association Consortium (OCAC), the Prostate Cancer Association Group to Investigate Cancer Associated Alterations in the Genome (PRACTICAL) and CIMBA. The general aims for designing the iCOGS array were to replicate findings from GWAS for identifying variants associated with breast, ovarian or prostate cancer (including subtypes and SNPs potentially associated with disease outcome), to facilitate fine-mapping of regions of interest, and to genotype “candidate” SNPs of interest within the consortia, including rarer variants. Each consortium was given a share of the array: nominally 25% of the SNPs each for BCAC, PRACTICAL and OCAC; 17.5% for CIMBA; and 7.5% for SNPs of common interest between the consortia. The final design comprised 220,123 SNPs, of which 211,155 were successfully manufactured. A total of 32,557 SNPs on the iCOGS array were selected based on 8 separate analyses of stage 1 of the CIMBA *BRCA1* GWAS that included 2,727 *BRCA1* mutation carriers [12]. After imputation for all SNPs in HapMap Phase II (CEU) a total of 2,568,349 (imputation r^2^>0.30) were available for analysis. Markers were evaluated for associations with: (1) breast cancer; (2) ovarian cancer; (3) breast cancer restricted to Class 1 mutations (loss-of-function mutations expected to result in a reduced transcript or protein level due to nonsense-mediated RNA decay); (4) breast cancer restricted to Class 2 mutations (mutations likely to generate stable proteins with potential residual or dominant negative function); (5) breast cancer by tumor ER-status; (6) breast cancer restricted to *BRCA1* 185delAG mutation carriers; (7) breast cancer restricted to *BRCA1* 5382insC mutation carriers; and (8) breast cancer by contrasting the genotype distributions in *BRCA1* mutation carriers, against the distribution in population-based controls. Analyses (1) and (2) were based on both imputed and observed genotypes, whereas the rest were based on only the observed genotypes. SNPs were ranked according to the 1 d.f. score-test for trend P-value (described below) and selected for inclusion based on nominal proportions of 61.5%, 20%, 2.5%, 2.5%, 2.5%, 0.5%, 0.5% and 10.0% for analyses (1) to (8). SNP duplications were not allowed and SNPs with a pairwise r^2^≥0.90 with a higher-ranking SNP were only allowed (up to a maximum of 2) if the P-value for association was <10^−4^ for analyses (1) and (2) and <10^−5^ for other analyses. SNPs with poor Illumina design scores were replaced by the SNP with the highest r^2^ (among SNPs with r^2^>0.80 based on HapMap data) that had a good quality design score. The analysis of associations with breast and ovarian cancer risks presented here included all 32,557 SNPs on iCOGS that were selected on the basis of the *BRCA1* GWAS.

### Genotyping and quality control

#### iCOGS genotyping

Genotyping was performed at Mayo Clinic. Genotypes for samples genotyped on the iCOGS array were called using Illumina's GenCall algorithm (Text S1). A total of 13,510 samples were genotyped for 211,155 SNPs. The sample and SNP QC process is summarised in Table S3. Of the 13,510 samples, 578 did not fulfil eligibility criteria based on phenotypic data and were excluded. A step-wise QC process was applied to the remaining samples and SNPs. Samples were excluded due to inferred gender errors, low call rates (<95%), low or high heterozygosity and sample duplications (cryptic and intended). Of the 211,155 markers genotyped, 9,913 were excluded due to Y-chromosome origin, low call rates (<95%), monomorphic SNPs, or SNPs with Hardy-Weinberg equilibrium (HWE) P<10^−7^ under a country-stratified test statistic [15] (Table S3). SNPs that gave discordant genotypes among known sample duplicates were also excluded. Multi-dimensional scaling was used to exclude individuals of non-European ancestry. We selected 37,149 weakly correlated autosomal SNPs (pair-wise r^2^<0.10) to compute the genomic kinship between all pairs of *BRCA1* carriers, along with 197 HapMap samples (CHB, JPT, YRI and CEU). These were converted to distances and subjected to multidimensional scaling (Figure S1). Using the first two components, we calculated the proportion of European ancestry for each individual [12] and excluded samples with >22% non-European ancestry (Figure S1). A total of 11,705 samples and 201,242 SNPs were available for analysis, including 31,812 SNPs selected by the *BRCA1* GWAS. The genotyping cluster plots for all SNPs that demonstrated genome-wide significance level of association or are presented below, were checked manually for quality (Figure S2).

#### iPLEX analysis

The most significant SNPs from 4 loci associated with ovarian cancer and 8 loci associated with breast cancer were selected (17 SNPs in total) for stage 3 genotyping. Genotyping using the iPLEX Mass Array platform was performed at Mayo Clinic. CIMBA QC procedures were applied. Samples that failed for ≥20% of the SNPs were excluded from the analysis. No SNPs failed HWE (P<0.01). The concordance among duplicates was ≥98%. Mutation carriers of self-reported non-European ancestry were excluded. A total of 2,646 *BRCA1* samples were eligible for analysis after QC.

### Statistical methods

The main analyses were focused on the evaluation of associations between each genotype and breast cancer or ovarian cancer risk separately. Analyses were carried out within a survival analysis framework. In the breast cancer analysis, the phenotype of each individual was defined by age at breast cancer diagnosis or age at last follow-up. Individuals were followed until the age of the first breast cancer diagnosis, ovarian cancer diagnosis, or bilateral prophylactic mastectomy, whichever occurred first; or last observation age. Mutation carriers censored at ovarian cancer diagnosis were considered unaffected. For the ovarian cancer analysis, the primary endpoint was the age at ovarian cancer diagnosis. Mutation carriers were followed until the age of ovarian cancer diagnosis, or risk-reducing salpingo-oophorectomy (RRSO) or age at last observation. In order to maximize the number of ovarian cancer cases, breast cancer was not considered as a censoring event in this analysis, and mutation carriers who developed ovarian cancer after a breast cancer diagnosis were considered as affected in the ovarian cancer analysis.

#### Association analysis

The majority of mutation carriers were sampled through families seen in genetic clinics. The first tested individual in a family is usually someone diagnosed with cancer at a relatively young age. Such study designs tend to lead to an over-sampling of affected individuals, and standard analytical methods like Cox regression may lead to biased estimates of the risk ratios [16], [17]. To adjust for this potential bias the data were analyzed within a survival analysis framework, by modeling the retrospective likelihood of the observed genotypes conditional on the disease phenotypes. A detailed description of the retrospective likelihood approach has been published [17], [18]. The associations between genotype and breast cancer risk at both stages were assessed using the 1 d.f. score test statistic based on this retrospective likelihood [17], [18]. To allow for the non-independence among related individuals, we accounted for the correlation between the genotypes by estimating the kinship coefficient for each pair of individuals using the available genomic data [16], [19], [20] and by robust variance estimation based on reported family membership [21]. We chose to present P-values based on the kinship adjusted score test as it utilises the degree of relationship between individuals. A genome-wide level of significance of 5×10^−8^ was used [22]. These analyses were performed in R using the GenABEL [23] libraries and custom-written functions in FORTRAN and Python.

To estimate the magnitude of the associations (HRs), the effect of each SNP was modeled either as a per-allele HR (multiplicative model) or as genotype-specific HRs, and were estimated on the log-scale by maximizing the retrospective likelihood. The retrospective likelihood was fitted using the pedigree-analysis software MENDEL [17], [24]. As sample sizes varied substantially between contributing centers heterogeneity was examined at the country level. All analyses were stratified by country of residence and used calendar-year and cohort-specific breast cancer incidence rates for *BRCA1* [25]. Countries with small number of mutation carriers were combined with neighbouring countries to ensure sufficiently large numbers within each stratum (Table S2). USA and Canada were further stratified by reported Ashkenazi Jewish (AJ) ancestry due to large numbers of AJ carriers. In stage 3 analysis involving several countries with small numbers of mutation carriers, we assumed only 3 large strata (Europe, Australia, USA/Canada). The combined iCOGS stage and stage 3 analysis was also stratified by stage of the experiment. The analysis of associations by breast cancer ER-status was carried out by an extension of the retrospective likelihood approach to model the simultaneous effect of each SNP on more than one tumor subtype [26] (Text S1).

#### Competing risk analysis

The associations with breast and ovarian cancer risk simultaneously were assessed within a competing risk analysis framework [17] by estimating HRs simultaneously for breast and ovarian cancer risk. This analysis provides unbiased estimates of association with both diseases and more powerful tests of association in cases where an association exists between a variant and at least one of the diseases [17]. Each individual was assumed to be at risk of developing either breast or ovarian cancer, and the probabilities of developing each disease were assumed to be independent conditional on the underlying genotype. A different censoring process was used, whereby individuals were followed up to the age of the first breast or ovarian cancer diagnosis and were considered to have developed the corresponding disease. No follow-up was considered after the first cancer diagnosis. Individuals censored for breast cancer at the age of bilateral prophylactic mastectomy and for ovarian cancer at the age of RRSO were assumed to be unaffected for the corresponding disease. The remaining individuals were censored at the last observation age and were assumed to be unaffected for both diseases.

#### Imputation

For the SNP selection process, the MACH software was used to impute non-genotyped SNPs based on the phased haplotypes from HapMap Phase II (CEU, release 22). The IMPUTE2 software [27] was used to impute non-genotyped SNPs for samples genotyped on the iCOGS array (stage 1 and 2 only), based on the 1,000 Genomes haplotypes (January 2012 version). Associations between each marker and cancer risk were assessed using a similar score test to that used for the observed SNPs, but based on the posterior genotype probabilities at each imputed marker for each individual. In all analyses, we considered only SNPs with imputation information/accuracy r^2^>0.30.

#### Absolute breast and ovarian cancer risks by combined SNP profile

We estimated the absolute risk of developing breast and ovarian cancer based on the joint distribution of all SNPs that were significantly associated with risk for *BRCA1* mutation carriers based on methods previously applied to *BRCA2* carriers [28]. We assumed that the average, age-specific breast and ovarian cancer incidences for *BRCA1* mutation carriers, over all modifying loci, agreed with published penetrance estimates for *BRCA1* [25]. The model assumed independence among the modifying loci and we used only the SNP with the strongest evidence of association from each region. We used only loci identified through the *BRCA1* GWAS that exhibited associations at a genome-wide significance level, and loci that were identified through population-based GWAS of breast or ovarian cancer risk, but were also associated with those risks for *BRCA1* mutation carriers. For each SNP, we used the per-allele HR and minor allele frequencies estimated from the present study. Genotype frequencies were obtained under the assumption of HWE.

## Results

Samples from 11,705 *BRCA1* carriers from 45 centers in 25 countries yielded high-quality data for 201,242 SNPs on the iCOGS array. The array included 31,812 *BRCA1* GWAS SNPs, which were analyzed here for their associations with breast and ovarian cancer risk for *BRCA1* mutation carriers (Table S2). Of the 11,705 *BRCA1* mutation carriers, 2,387 samples had also been genotyped for stage 1 of the GWAS and 9,318 were unique to the stage 2 iCOGS study.

### Breast cancer associations

When restricting analysis to stage 2 samples (4,681 unaffected, 4,637 affected), there was little evidence of inflation in the association test-statistic (λ = 1.038; Figure S3). Combined analysis of stage 1 and 2 samples (5,784 unaffected, 5,920 affected) revealed 66 SNPs in 28 regions with P<10^−4^ (Figure S4). These included variants from three loci (19p13, 6q25.1, 12p11) previously associated with breast cancer risk for *BRCA1* mutation carriers (Table 1). Further evaluation of 18 loci associated with breast cancer susceptibility in the general population found that only the *TOX3*, *LSP1*, 2q35 and *RAD51L1* loci were significantly associated with breast cancer for *BRCA1* carriers (Table 1, Table S4).

**Table 1:** Associations with breast or ovarian cancer risk for loci previously reported to be associated with cancer risk for *BRCA1* mutation carriers.

Locus	Previously published association in *BRCA1*				Strongest association in current set of 31,812 *BRCA1* GWAS SNPs					Association for published SNP in set of all iCOGS SNPs			
	SNP	all1/all2 (freq)	HR (95%CI)	P	SNP	all1/all2 (freq)	r^2^	HR (95%CI)	P	Best tag SNP (r^2^)	all1/all2 (freq)	HR (95%CI)	P
*Loci previously associated with breast cancer risk for BRCA1 carriers*													
19p13	rs8170	G/A (0.17)	1.26 (1.17–1.35)	2.3×10^−9^	rs8100241	G/A (0.52)	0.31	0.84 (0.80–0.88)	4.3×10^−13^	rs8170 (1.0)	G/A (0.17)	1.22 (1.14–1.29)	4.8×10^−10^
6q25.1	rs2046210	C/T (0.35)	1.17 (1.11–1.23)	4.5×10^−9^	rs3734805	A/C (0.08)	0.25	1.28 (1.18–1.39)	5×10^−9^	rs2046210* (1.0)	G/A (0.35)	1.15 (1.10–1.21)	2.8×10^−8^
12p11	rs10771399	A/G (0.11)	0.87 (0.81–0.94)	3.2×10^−4^	rs7957915	A/G (0.14)	0.85	0.85 (0.79–0.91)	8.1×10^−6^	rs10771399*	A/G (0.11)	0.85 (0.79–0.92)	2.7×10^−5^
*TOX3*	rs3803662	C/T (0.29)	1.09 (1.03–1.16)	0.0049	rs4784220	A/G (0.38)	0.52	1.08 (1.03–1.13)	0.0021	rs3803662*	G/A (0.29)	1.05 (1.00–1.11)	0.075
2q35	rs13387042^a^	G/A (0.52)	1.02 (0.96–1.07)	0.57	rs13389571	A/G (0.05)	0.02	0.86 (0.77–0.96)	0.011	rs13387042*	A/G (0.48)	1.01 (0.96–1.06)	0.74
*Known ovarian cancer susceptibility loci*													
9p22	rs3814113	T/C (0.34)	0.78 (0.72–0.85)	4.8×10^−9^	rs3814113	A/G (0.34)	1.00	0.77 (0.71–0.83)	5.9×10^−11^	rs3814113 (1.0)	A/G (0.34)	0.77 (0.71–0.83)	5.9×10^−11^
2q31	rs2072590	T/C (0.31)	1.06 (0.98–1.14)	0.16	rs1026032	A/G (0.26)	0.75	1.08 (0.99–1.17)	0.064	rs2072590* (1.0)	C/A (0.32)	1.05 (0.97–1.14)	0.20
8q24	rs10088218	G/A (0.13)	0.89 (0.81–0.99)	0.029	rs9918771	A/C (0.17)	0.31	0.86 (0.78–0.95)	0.0021	rs10088218 (1.0)	G/A (0.13)	0.86 (0.78–0.96)	0.0096
3q25	rs2665390	T/C (0.075)	1.25 (1.10–1.42)	2.7×10^−3^	rs7651446	C/A (0.043)	0.71	1.46 (1.25–1.71)	6.6×10^−6^	rs344008 (1.0)	G/A (0.075)	1.21 (1.07–1.38)	3.8×10^−3^
17q21	rs9303542	T/C (0.26)	1.08 (1.00–1.17)	0.06	rs11651753	G/A (0.43)	0.36	1.14 (1.06–1.23)	4.6×10^−4^	rs9303542* (1.0)	A/G (0.26)	1.12 (1.04–1.22)	8.0×10^−3^
19p13^b^	rs67397200	C/G (0.28)	1.16 (1.05–1.29)	3.8×10^−4^	c19_pos17158477	G/C (0.038)	0.01	0.64 (0.49–0.83)	7.0×10^−4^	rs67397200 (c19_pos17262404)	G/C (0.28)	1.12 (1.01–1.23)	0.027

Freq = frequency of allele 2 in unaffected *BRCA1* carriers.

HR = Per allele Hazard Ratio associated with allele 2, under a single disease risk model, unless specified.

r^2^: correlation between the SNP in the present study and the published SNP.

* SNP not in *BRCA1* GWAS SNP allocation on iCOGS chip.

a : rs13387042 was previously found to be associated only under the 2-df model.

b : analysis under a competing risks model.

After excluding SNPs from the known loci, there were 39 SNPs in 25 regions with P = 1.2×10^−6^–1.0×10^−4^. Twelve of these SNPs were genotyped by iPLEX in an additional 2,646 *BRCA1* carriers (1,252 unaffected, 1,394 affected, “stage 3” samples, Table S5). There was additional evidence of association with breast cancer risk for four SNPs at two loci (P<0.01, Table 2). When all stages were combined, SNPs rs2290854 and rs6682208 (r^2^ = 0.84) at 1q32, near *MDM4*, had combined *P*-values of association with breast cancer risk of 1.4×10^−7^ and 4×10^−7^,respectively. SNPs rs11196174 and rs11196175 (r^2^ = 0.96) at 10q25.3 (in *TCF7L2*) had combined P-values of 7.5×10^−7^ and 1.2×10^−6^. Analysis within a competing risks framework, where associations with breast and ovarian cancer risks are evaluated simultaneously [17], revealed stronger associations with breast cancer risk for all 4 SNPs, but no associations with ovarian cancer (Table 3). In particular, we observed a genome-wide significant association between the minor allele of rs2290854 from 1q32 and breast cancer risk (per-allele HR: 1.14; 95%CI: 1.09–1.20; p = 2.7×10^−8^). Country-specific HR estimates for all SNPs are shown in Figure S5. Analyses stratified by *BRCA1* mutation class revealed no significant evidence of a difference in the associations of any of the SNPs by the predicted functional consequences of *BRCA1* mutations (Table S6). SNPs in the *MDM4* and *TCF7L2* loci were associated with breast cancer risk for both class1 and class2 mutation carriers.

**Table 2:** Associations with breast and ovarian cancer risk for SNPs found to be associated with risk at all 3 stages of the experiment.

SNP, Chr, Position, Allele1/Allele2	Stage	Number		Allele 2 Frequency		HR* (95% CI)			
		Unaffected	Affected	Unaffected	Affected	Per Allele	Heterozygote	Homozygote	P-trend
***Breast Cancer***									
rs2290854, 1q32, 202782648, G/A	Stage 1	1104	1283	0.30	0.34	1.19 (1.08–1.30)	1.28 (1.12–1.47)	1.31 (1.06–1.61)	4.2×10^−4^
	Stage 2	4681	4637	0.31	0.33	1.09 (1.03–1.16)	1.10 (1.02–1.19)	1.18 (1.03–1.35)	0.003
	Stages1+2	5785	5920	0.31	0.33	1.12 (1.06–1.17)	1.15 (1.07–1.23)	1.21 (1.08–1.36)	1.7×10^−5^
	Stage 3	1252	1393	0.30	0.33	1.19 (1.07–1.32)	1.24 (1.07–1.43)	1.36 (1.06–1.74)	0.0013
	Combined	7037	7313	0.31	0.33	1.13 (1.08–1.18)	1.16 (1.09–1.24)	1.24 (1.11–1.37)	1.4×10^−7^
rs6682208, 1q32, 202832806, G/A	Stage 1	1104	1283	0.32	0.35	1.14 (1.04–1.25)	1.24 (1.09–1.42)	1.20 (0.98–1.47)	0.0070
	Stage 2	4681	4637	0.32	0.34	1.10 (1.04–1.17)	1.09 (1.01–1.19)	1.21 (1.06–1.38)	0.0014
	Stages1+2	5785	5920	0.32	0.34	1.11 (1.05–1.17)	1.13 (1.05–1.21)	1.21 (1.08–1.35)	5.4×10^−5^
	Stage 3	1250	1394	0.30	0.34	1.19 (1.07–1.32)	1.31 (1.14–1.51)	1.28 (1.01–1.63)	8.6×10^−4^
	Combined	7035	7314	0.32	0.34	1.12 (1.07–1.17)	1.16 (1.09–1.23)	1.22 (1.11–1.35)	4.3×10^−7^
rs11196174, 10q25.3, 114724086, A/G	Stage 1	1103	1282	0.27	0.32	1.15 (1.05–1.27)	1.17 (1.03–1.34)	1.31 (1.05–1.63)	0.0038
	Stage 2	4681	4636	0.29	0.31	1.10 (1.04–1.17)	1.13 (1.04–1.23)	1.17 (1.01–1.35)	0.0017
	Stages1+2	5784	5918	0.28	0.31	1.12 (1.06–1.18)	1.14 (1.06–1.23)	1.21 (1.07–1.37)	3.1×10^−5^
	Stage 3	1251	1393	0.28	0.31	1.16 (1.05–1.29)	1.08 (0.93–1.25)	1.46 (1.15–1.85)	0.0057
	Combined	7035	7311	0.28	0.31	1.13 (1.07–1.18)	1.13 (1.06–1.21)	1.26 (1.13–1.40)	7.5×10^−7^
rs11196175, 10q25.3, 114726604, A/G	Stage 1	1101	1280	0.27	0.31	1.15 (1.05–1.27)	1.18 (1.03–1.35)	1.29 (1.03–1.62)	0.0043
	Stage 2	4674	4627	0.28	0.30	1.10 (1.03–1.17)	1.13 (1.04–1.22)	1.17 (1.01–1.35)	0.0020
	Stages1+2	5775	5907	0.28	0.30	1.12 (1.06–1.18)	1.14 (1.06–1.22)	1.21 (1.07–1.37)	3.9×10^−5^
	Stage 3	1251	1394	0.27	0.31	1.16 (1.04–1.29)	1.06 (0.91–1.22)	1.48 (1.17–1.87)	0.0075
	Combined	7026	7301	0.28	0.31	1.12 (1.07–1.18)	1.12 (1.05–1.20)	1.26 (1.13–1.41)	1.2×10^−6^
***Ovarian Cancer***									
rs17631303, 17q21, 40872185, A/G	Stage 1	1797	574	0.19	0.25	1.46 (1.22–1.74)	1.36 (1.01–1.68)	2.46 (1.53–3.96)	1.3×10^−5^
	Stage 2	7996	1257	0.19	0.21	1.20 (1.07–1.35)	1.10 (0.96–1.26)	1.83 (1.34–2.48)	1.5×10^−3^
	Stages1+2	9793	1831	0.19	0.22	1.27 (1.16–1.40)	1.15 (1.03–1.29)	2.03 (1.16–2.61)	3.0×10^−7^
	Stage 3	2204	442	0.17	0.21	1.27 (1.07–1.51)	1.24 (0.99–1.56)	1.67 (1.07–2.62)	0.014
	Combined	11997	2273	0.19	0.22	1.27 (1.17–1.38)	1.17 (1.06–1.29)	1.95 (1.57–2.42)	1.4×10^−8^
rs183211, 17q21, 42143493, G/A	Stage 1	1812	575	0.22	0.28	1.45 (1.23–1.71)	1.37 (1.11–1.69)	2.29 (1.53–3.41)	2.5×10^−5^
	Stage 2	8054	1264	0.23	0.25	1.20 (1.07–1.33)	1.13 (0.99–1.28)	1.62 (1.22–2.14)	1.1×10^−3^
	Stages1+2	9866	1839	0.23	0.26	1.25 (1.15–1.37)	1.16 (1.04–1.29)	1.83 (1.46–2.28)	5.7×10^−7^
	Stage 3	2204	442	0.22	0.26	1.25 (1.06–1.48)	1.15 (0.92–1.44)	1.79 (1.21–2.67)	0.018
	Combined	12070	2281	0.23	0.26	1.25 (1.16–1.35)	1.16 (1.05–1.27)	1.82 (1.5–2.21)	3.1×10^−8^
rs4691139, 4q32.3, 166128171, A/G	Stage 1	1812	575	0.47	0.53	1.24 (1.08–1.42)	1.46 (1.13–1.88)	1.55 (1.16–2.05)	3.6×10^−3^
	Stage 2	8054	1264	0.48	0.52	1.18 (1.08–1.29)	1.29 (1.10–1.50)	1.40 (1.17–1.67)	1.3×10^−4^
	Stages1+2	9866	1839	0.48	0.52	1.20 (1.11–1.29)	1.33 (1.17–1.52)	1.44 (1.24–1.67)	1.1×10^−6^
	Stage 3	2204	441	0.47	0.52	1.20 (1.04–1.39)	1.19 (0.91–1.54)	1.44 (1.08–1.94)	9×10^−3^
	Combined	12070	2280	0.48	0.52	1.20 (1.17–1.38)	1.30 (1.16–1.46)	1.44 (1.26–1.65)	3.4×10^−8^

* HRs estimated under the single disease risk models.

**Table 3:** Analysis of associations with breast and ovarian cancer risk simultaneously (competing risks analysis) for SNPs found to be associated with breast or ovarian cancer.

SNP, Chr, Position, Allele1/Allele2	Unaffected (Allele2 Freq)	Ovarian Cancer (Allele2 Freq)	Breast Cancer (Allele2 Freq)	Ovarian Cancer		Breast Cancer	
				HR (95% CI)	P	HR (95% CI)	P
*SNPs found to be associated with breast cancer risk.*							
rs2290854, 1q32, 202782648, G/A	5473 (0.31)	1618 (0.31)	7259 (0.33)	1.08 (0.99–1.18)	0.08	1.14 (1.09–1.20)	2.7×10^−8^
rs6682208, 1q32, 202832806, G/A	5471 (0.32)	1618 (0.33)	7260 (0.34)	1.08 (1.00–1.18)	0.06	1.13 (1.08–1.19)	1.2×10^−7^
rs11196174, 10q25.3, 114724086, A/G	5471 (0.28)	1618 (0.29)	7257 (0.31)	1.07 (0.98–1.16)	0.16	1.14 (1.08–1.19)	3.2×10^−7^
rs11196175, 10q25.3, 114726604, A/G	5465 (0.28)	1615 (0.29)	7247 (0.31)	1.07 (0.97–1.16)	0.16	1.14 (1.08–1.19)	3.9×10^−7^
*SNPs found to be associated with ovarian cancer risk*							
rs17631303, 17q21, 40872185, A/G	5445 (0.19)	1610 (0.22)	7215 (0.19)	1.26 (1.14–1.39)	1.0×10^−5^	1.02 (0.96–1.08)	0.52
rs183211, 17q21, 42143493, G/A	5473 (0.23)	1618 (0.26)	7260 (0.23)	1.25 (1.14–1.38)	3.5×10^−6^	1.02 (0.97–1.08)	0.42
rs4691139, 4q32.3, 166128171, A/G	5473 (0.48)	1617 (0.53)	7269 (0.48)	1.21 (1.12–1.31)	2.8×10^−6^	0.98 (0.93–1.02)	0.28

Both the 1q32 and 10q25.3 loci were primarily associated with ER-negative breast cancer for *BRCA1* (rs2290854: ER-negative HR = 1.16, 95%CI: 1.10–1.22, P = 1.2×10^−7^; rs11196174: HR = 1.14, 95%CI: 1.07–1.20, P = 9.6×10^−6^), although the differences between the ER-negative and ER-positive HRs were not significant (Table S7). Given that ER-negative breast cancers in *BRCA1* and *BRCA2* mutation carriers are phenotypically similar [29], we also evaluated associations between these SNPs and ER-negative breast cancer in 8,211 *BRCA2* mutation carriers. While the 10q25.3 SNPs were not associated with overall or ER-negative breast cancer risk for *BRCA2* carriers, the 1q32 SNPs were associated with ER-negative (rs2290854 HR = 1.16, 95%CI:1.01–1.34, P = 0.033; rs6682208 HR = 1.19, 95%CI:1.04–1.35, P = 0.016), but not ER-positive breast cancer (rs2290854 P-diff = 0.006; rs6682208 P-diff = 0.001). Combining the *BRCA1* and *BRCA2* samples provided strong evidence of association with ER-negative breast cancer (rs2290854: P = 1.25×10^−8^; rs6682208: P = 2.5×10^−7^).

The iCOGS array included additional SNPs from the 1q32 region that were not chosen based on the *BRCA1* GWAS. Of these non-*BRCA1* GWAS SNPs, only SNP rs4951407 was more significantly associated with risk than the *BRCA1*-GWAS selected SNPs (P = 3.3×10^−6^, HR = 1.12, 95%CI:1.07–1.18, using stage 1 and stage 2 samples). The evidence of association with breast cancer risk was again stronger under the competing risks analysis (HR = 1.14, 95%CI: 1.08–1.20, P = 6.1×10^−7^). Backward multiple regression analysis, considering only the genotyped SNPs (P<0.01), revealed that the most parsimonious model included only rs4951407. SNPs from the 1000 Genomes Project, were imputed for the stage 1 and stage 2 samples (Figure S6). Only imputed SNP rs12404974, located between *PIK3C2B* and *MDM4* (r^2^ = 0.77 with rs4951407), was more significantly associated with breast cancer (P = 2.7×10^−6^) than any of the genotyped SNPs. None of the genotyped or imputed SNPs from 10q25.3 provided P-values smaller than those for rs11196174 and rs11196175 (Figure S7).

### Ovarian cancer associations

Analyses of associations with ovarian cancer risk using the stage 2 samples (8,054 unaffected, 1,264 affected) revealed no evidence of inflation in the association test-statistic (λ = 1.039, Figure S3). In the combined analysis of stage 1 and 2 samples (9866 unaffected, 1839 affected), 62 SNPs in 17 regions were associated with ovarian cancer risk for *BRCA1* carriers at P<10^−4^ (Figure S3). These included SNPs in the 9p22 and 3q25 loci previously associated with ovarian cancer risk in both the general population and *BRCA1* carriers [6], [7] (Table 1). Associations (P<0.01) with ovarian cancer risk were also observed for SNPs in three other known ovarian cancer susceptibility loci (8q24, 17q21, 19p13), but not 2q31 (Table 1). For all loci except 9p22, SNPs were identified that displayed smaller P-values of association than previously published results [5]–[7].

After excluding SNPs from known ovarian cancer susceptibility regions, there were 48 SNPs in 15 regions with P = 5×10^−7^ to 10^−4^. Five SNPs from four of these loci were genotyped in the stage 3 samples (2,204 unaffected, 442 with ovarian cancer). Three SNPs showed additional evidence of association with ovarian cancer risk (P<0.02, Table 2; Table S5). In the combined stage 1–3 analyses, SNPs rs17631303 and rs183211 (r^2^ = 0.68) on chromosome 17q21.31 had P-values for association of 1×10^−8^ and 3×10^−8^ respectively, and rs4691139 at 4q32.3 had a P-value of 3.4×10^−8^ (Table 2).

The minor alleles of rs17631303 (HR = 1.27, 95%CI:1.17–1.38) and rs183211 (HR = 1.25, 95%CI: 1.16–1.35) at 17q21.31 were associated with increased ovarian cancer risk (Table 2). Analysis of the associations within a competing risks framework, revealed no association with breast cancer risk (Table 3). The ovarian cancer effect size was maintained in the competing risk analysis but the significance of the association was slightly weaker (P = 2×10^−6^–1×10^−5^). This is expected because 663 ovarian cancer cases occurring after a primary breast cancer diagnosis were excluded for this analysis. The evidence of association was somewhat stronger under the genotype-specific model (2-df P = 1.6×10^−9^ and P = 2.6×10^−9^ for rs17631303 and rs183211 respectively in all samples combined) with larger HR estimates for the rare homozygote genotypes than those expected under a multiplicative model (Table 2).

Previous studies of the known common ovarian cancer susceptibility alleles found significant associations with ovarian cancer for both *BRCA1* and *BRCA2* carriers [6], [7]. Thus, we evaluated the associations between the 17q21.31 SNPs and ovarian cancer risk for *BRCA2* carriers using iCOGS genotype data (7580 unaffected and 631 affected). Both rs17631303 and rs183211 were associated with ovarian cancer risk for *BRCA2* carriers (P = 1.98×10^−4^ and 9.26×10^−4^), with similar magnitude and direction of association as for *BRCA1* carriers. Combined analysis of *BRCA1* and *BRCA2* mutation carriers provided strong evidence of association (P = 2.80×10^−10^ and 2.01×10^−9^, Table 4).

**Table 4:** Associations with SNPs at the novel 17q21 region with ovarian cancer risk for *BRCA1* and *BRCA2* mutation carriers.

SNP, Allele1/Allele2	*BRCA1* (Stage 1 & 2 samples)				*BRCA2*				*BRCA1* & *BRCA2* samples combined
	Unaffected (All2 freq)	Ovarian Cancer (All2 freq)	HR* (95%CI)	P-trend	Unaffected (All2 freq)	Ovarian Cancer (All2 freq)	HR* (95%CI)	P-trend	P-trend
rs17631303, A/G	9793 (0.19)	1831 (0.22)	1.27 (1.16–1.40)	3.04×10^−7^	7481 (0.19)	626 (0.24)	1.32 (1.15–1.52)	1.98×10^−4^	2.80×10^−10^
rs2077606, G/A	9736 (0.19)	1810 (0.22)	1.27 (1.15–1.40)	5.51×10^−7^	7421 (0.19)	613 (0.23)	1.31 (1.13–1.50)	5.60×10^−4^	1.27×10^−9^
rs2532348, A/G	9511 (0.21)	1789 (0.24)	1.25 (1.14–1.37)	8.71×10^−7^	7407 (0.23)	615 (0.28)	1.33 (1.17–1.51)	4.62×10^−5^	2.49×10^−10^
rs183211, G/A	9866 (0.23)	1839 (0.26)	1.25 (1.15–1.37)	5.67×10^−7^	7580 (0.25)	631 (0.30)	1.26 (1.11–1.43)	9.26×10^−4^	2.01×10^−9^
rs169201, A/G	9865 (0.20)	1839 (0.23)	1.27 (1.15–1.37)	5.04×10^−7^	7578 (0.21)	631 (0.26)	1.36 (1.19–1.55)	1.72×10^−5^	6.24×10^−11^
rs199443, G/A	9849 (0.20)	1835 (0.23)	1.26 (1.15–1.39)	5.15×10^−7^	7580 (0.21)	631 (0.26)	1.35 (1.18–1.54)	2.57×10^−5^	8.87×10^−11^
rs199534, A/C	9865 (0.20)	1839 (0.23)	1.26 (1.15–1.39)	6.26×10^−7^	7575 (0.21)	630 (0.26)	1.35 (1.18–1.55)	1.90×10^−5^	8.57×10^−11^

* HRs estimated under the single disease risk model.

**Figure 2:**
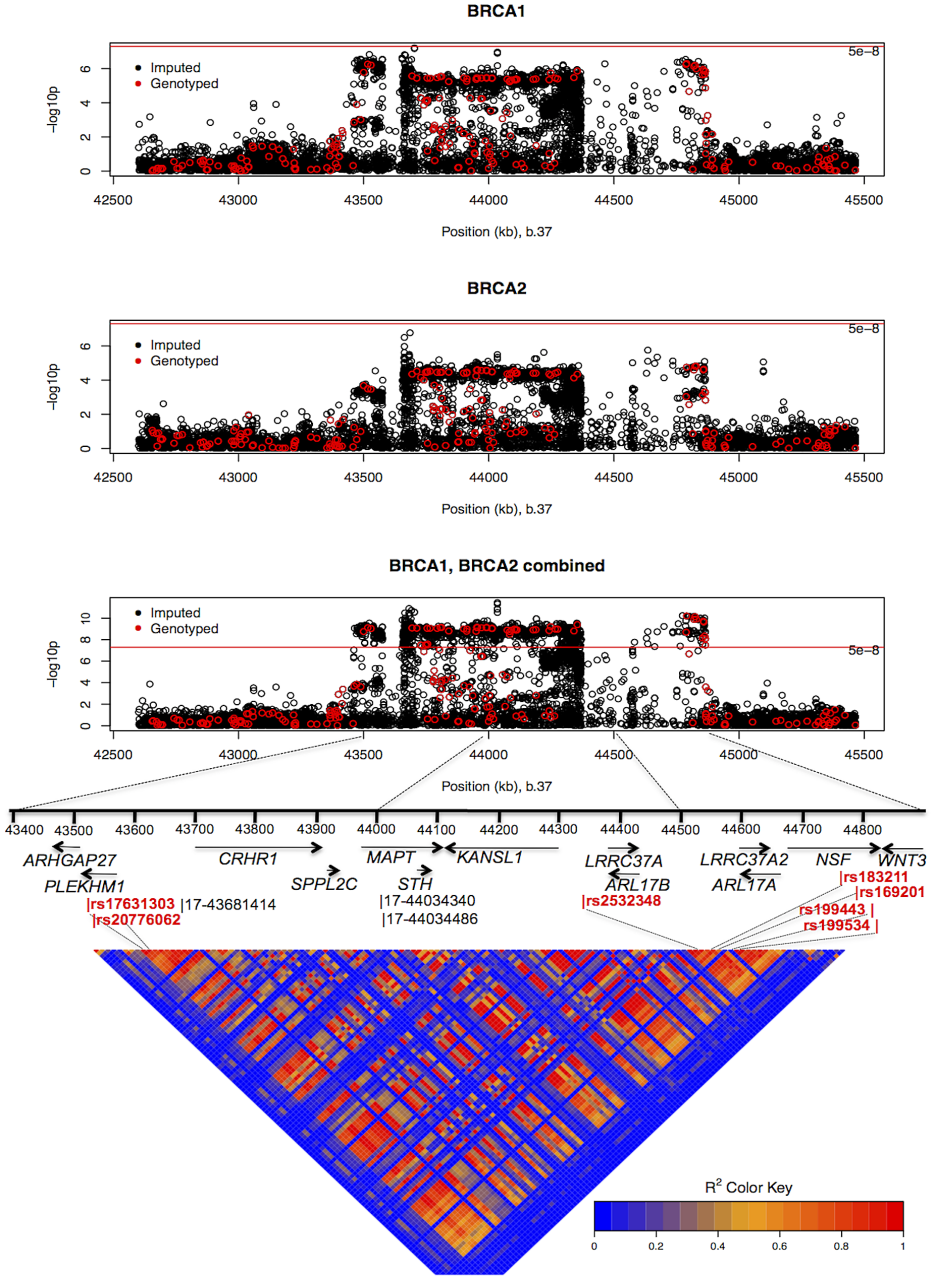
Mapping of the 17q21 locus. *Top 3 panels:* P-values of association (−log_10_ scale) with ovarian cancer risk for genotyped and imputed SNPs (1000 Genomes Project CEU), by chromosome position (b.37) at the 17q21 region, for *BRCA1*, *BRCA2* mutation carriers and combined. Results based on the kinship-adjusted score test statistic (1 d.f.). *Fourth panel:* Genes in the region spanning (43.4–44.9 Mb, b.37) and the location of the most significant genotyped SNPs (in red font) and imputed SNPs (in black font). *Bottom panel:* Pairwise r^2^ values for genotyped SNPs on iCOG array in the 17q21 region covering positions (43.4–44.9 Mb, b.37).

The combined analysis of stage 1 and 2 samples, and *BRCA2* carriers, identified seven SNPs on the iCOGS array (pairwise r^2^ range: 0.68–1.00) from a 1.3 Mb (40.8–42.1 Mb, build 36.3) region of 17q21.31 that were strongly associated (P<1.27×10^−9^) with ovarian cancer risk (Table 4, Figure 2). Stepwise-regression analysis based on observed genotype data retained only one of the seven SNPs in the model, but it was not possible to distinguish between the SNPs. Imputation through the 1000 Genomes Project, revealed several SNPs in 17q21.31 with stronger associations (Figure 2, Table S8) than the most significant genotyped SNP in the combined *BRCA1/2* analysis (rs169201, P = 6.24×10^−11^). The most significant SNP (rs140338099 (17-44034340), P = 3×10^−12^), located in *MAPT*, was highly correlated (r^2^ = 0.78) with rs169201 in *NSF* (Figure 2). This locus appears to be distinct from a previously identified ovarian cancer susceptibility locus located >1 Mb distal on 17q21 (spanning 43.3–44.3 Mb, build 36.3) [30]. None of the SNPs in the novel region were strongly correlated with any of the SNPs in the 43.3–44.3 Mb region (maximum r^2^ = 0.07, Figure S8). The most significantly associated SNP from the *BRCA1* GWAS from the 43.3–44.3 Mb locus was rs11651753 (p = 4.6×10^−4^) (Table 1) (r^2^<0.023 with the seven most significant SNPs in the novel 17q21.31 region). An analysis of the joint associations of rs11651753 and rs17631303 from the two 17q21 loci with ovarian cancer risk for *BRCA1* carriers (Stage 1 and 2 samples) revealed that both SNPs remained significant in the model (P-for inclusion = 0.001 for rs11651753, 1.2×10^−6^ for rs17631303), further suggesting that the two regions are independently associated with ovarian cancer for *BRCA1* carriers.

The minor allele of rs4691139 at the novel 4q32.3 region was also associated with an increased ovarian cancer risk for *BRCA1* carriers (per-allele HR = 1.20, 95%CI:1.17–1.38, Table 2), but was not associated with breast cancer risk (Table 3). No other SNPs from the 4q32.3 region on the iCOGS array were more significantly associated with ovarian cancer for *BRCA1* carriers. Analysis of associations with variants identified through 1000 Genomes Project-based imputation of the Stage 1 and 2 samples, revealed 19 SNPs with stronger evidence of association (P = 5.4×10^−7^ to 1.1×10^−6^) than rs4691139 (Figure S9). All were highly correlated (pairwise r^2^>0.89) and the most significant (rs4588418) had r^2^ = 0.97 with rs4691139. There was no evidence for association between rs4691139 and ovarian cancer risk for *BRCA2* carriers (HR = 1.08, 95%CI: 0.96–1.21, P = 0.22).

### Absolute risks of developing breast and ovarian cancer

**Figure 3:**
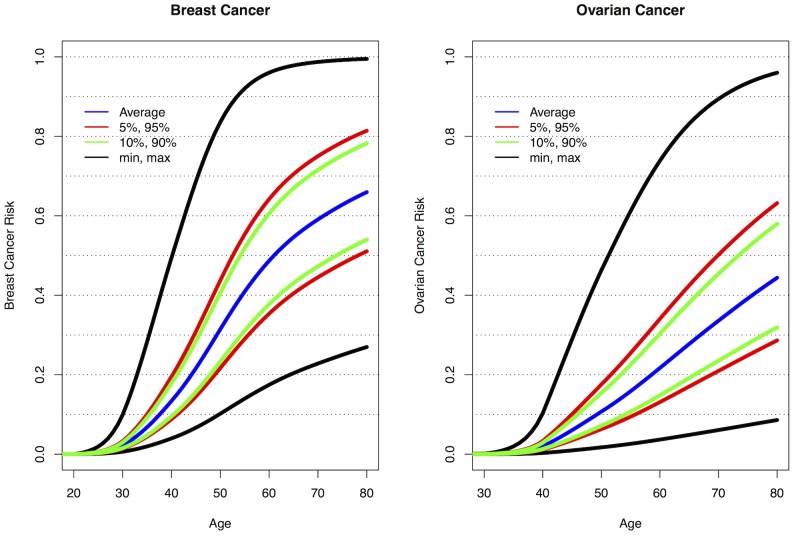
Predicted breast and ovarian cancer absolute risks for *BRCA1* mutation carriers at the 5^th^, 10^th^, 90^th^, and 95^th^ percentiles of the combined SNP profile distributions. The minimum, maximum and average risks are also shown. Predicted cancer risks are based on the associations of known breast or ovarian cancer susceptibility loci (identified through GWAS) with cancer risk for *BRCA1* mutation carriers and loci identified through the present study. Breast cancer risks based on the associations with: 1q32, 10q25.3, 19p13, 6q25.1, 12p11, *TOX3*, 2q35, *LSP1*, *RAD51L1* (based on HR and minor allele frequency estimates from Table 1, Table 2, and Table S4) and *TERT* [31]. Ovarian cancer risks based on the associations with: 9p22, 8q24, 3q25, 17q21, 19p13 (Table 1) and 17q21.31, 4q32.3 (Table 2). Only the top SNP from each region was chosen. Average breast and ovarian cancer risks were obtained from published data [25]. The methods for calculating the predicted risks have been described previously [28].

The current analyses suggest that 10 loci are now known to be associated with breast cancer risk for *BRCA1* mutation carriers: 1q32, 10q25.3, 19p13, 6q25.1, 12p11, *TOX3*, 2q35, *LSP1* and *RAD51L1* all reported here and *TERT* [31]. Similarly, seven loci are associated with ovarian cancer risk for *BRCA1* mutation carriers: 9p22, 8q24, 3q25, 17q21, 19p13, 17q21.31 and 4q32.3. Figure S10 shows the range of combined HRs at different percentiles of the combined genotype distribution, based on the single SNP HR and minor allele estimates from Table 1, Table 2, and Table S4 and for TERT from Bojesen et al [31] and assuming that all SNPs interact multiplicatively. Relative to *BRCA1* mutation carriers at lowest risk, the median, 5^th^ and 95^th^ percentile breast cancer HRs were 3.40, 2.27, and 5.35 respectively. These translate to absolute risks of developing breast cancer by age 80 of 65%, 51% and 81% for those at median, 5^th^ and 95^th^ percentiles of the combined genotype distribution (Figure 3, Figure S10). Similarly, the median, 5^th^ and 95^th^ percentile combined HRs for ovarian cancer were 6.53, 3.75 and 11.12 respectively, relative to those at lowest ovarian cancer risk (Figure S10). These HRs translate to absolute risks of developing ovarian cancer of 44%, 28% and 63% by age 80 for the median, 5^th^ and 95^th^ percentile of the combined genotype distribution (Figure 3).

## Discussion

In this study we analyzed data from 11,705 *BRCA1* mutation carriers from CIMBA who were genotyped using the iCOGS high-density custom array, which included 31,812 SNPs selected on the basis of a *BRCA1* GWAS. This study forms the large-scale replication stage of the first GWAS of breast and ovarian cancer risk modifiers for *BRCA1* mutation carriers. We have identified a novel locus at 1q32, containing the *MDM4* oncogene, that is associated with breast cancer risk for *BRCA1* mutation carriers (P<5×10^−8^). A separate locus at 10q23.5, containing the *TCF7L2* gene, provided strong evidence of association with breast cancer risk for *BRCA1* carriers but did not reach a GWAS level of significance. We have also identified two novel loci associated with ovarian cancer for *BRCA1* mutation carriers at 17q21.31 and 4q32.2 (P<5×10^−8^). We further confirmed associations with loci previously shown to be associated with breast or ovarian cancer risk for *BRCA1* mutation carriers. In most cases stronger associations were detected with either the same SNP reported previously (due to increased sample size) or other SNPs in the regions. Future fine mapping studies of these loci will aim to identify potentially causal variants for the observed associations.

Although the 10q25.3 locus did not reach the strict GWAS level of significance for association with breast cancer risk, the association was observed at all three independent stages of the experiment. Additional evidence for the involvement of this locus in breast cancer susceptibility comes from parallel studies of the Breast Cancer Association Consortium (BCAC). SNPs at 10q25.3 had also been independently selected for inclusion on the iCOGS array through population based GWAS of breast cancer. Analyses of those SNPs in BCAC iCOGS studies also found that SNPs at 10q25.3 were associated with breast cancer risk in the general population [32]. Thus, 10q25.3 is likely a breast cancer risk-modifying locus for *BRCA1* mutation carriers. The most significant SNPs at 10q25.3 were located in *TCF7L2*, a transcription factor that plays a key role in the Wnt signaling pathway and in glucose homeostasis, and is expressed in normal and malignant breast tissue (The Cancer Genome Atlas (TCGA)). Variation in the *TCF7L2* locus has previously been associated with Type 2 diabetes in a number of GWAS. The most significantly associated SNPs with Type 2 diabetes (rs7903146 and rs4506565) [22], [33] were also associated with breast cancer risk for *BRCA1* mutation carriers in stage 1 and 2 analyses (p = 3.7×10^−4^ and p = 2.5×10^−4^ respectively); these SNPs were correlated with the most significant hit (rs11196174) for *BRCA1* breast cancer (r^2^ = 0.40 and 0.37 based on stage 1 and 2 samples). This raises the possibility that variants in this locus influence breast cancer indirectly through effects on cellular metabolism.

We found that SNPs at 1q32 were primarily associated with ER-negative breast cancer risk for *BRCA1* mutation carriers. There was also evidence of association with ER-negative breast cancer for *BRCA2* mutation carriers. SNPs at the 1q32 region were independently selected for inclusion on iCOGS through GWAS of breast cancer in the general population by BCAC. In parallel analyses of iCOGS data by BCAC, 1q32 was found to be associated with ER-negative breast cancer [34] but not overall breast cancer risk [32]. Taken together, these results are in agreement with our findings and in line with the observation that the majority of *BRCA1* breast cancers are ER-negative. However, they are not in agreement with a previous smaller candidate-gene study that found an association between a correlated SNP in *MDM4* (r^2^>0.85) and overall breast cancer risk [35]. The 1q32 locus includes the *MDM4* oncogene which plays a role in regulation of p53 and MDM2 and the apoptotic response to cell stress. *MDM4* is expressed in breast tissue and is amplified and overexpressed along with *LRRN2* and *PIK3C2B* in breast and other tumor types (TCGA) [36]–[38]. Although fine mapping will be necessary to identify the functionally relevant SNPs in this locus, we found evidence of cis-regulatory variation impacting *MDM4* expression [39]–[41] (Text S1, Table S9, Figure S11), suggesting that common variation in the 1q32 locus may influence the risk of breast cancer through direct effects on *MDM4* expression.

Several correlated SNPs at 17q21.31 from the iCOGS array provided strong evidence of association with ovarian cancer risk in both *BRCA1* and *BRCA2* mutation carriers. A subsequent analysis of these SNPs, which were selected through the *BRCA1* GWAS, in case-control samples from the Ovarian Cancer Association Consortium (OCAC), revealed that the 17q21.31 locus is associated with ovarian cancer risk in the general population [Wey et al, personal communication]. Thus, 17q21.31 is likely a novel susceptibility locus for ovarian cancer in *BRCA1* mutation carriers. The most significant associations at 17q21.31 were clustered in a large region of strong linkage disequilibrium which has previously been identified as a “17q21.31 inversion” (∼900 kb long) consisting of two haplotypes (termed H1 and H2) [42]. The minor allele of rs2532348 (MAF = 0.21), which tags H2, was associated with increased ovarian cancer risk for *BRCA1* mutation carriers (Table 4). The 1.3 Mb 17q21.31 locus contains 13 genes and several predicted pseudogenes (Figure 2), several of which are expressed in normal ovarian surface epithelium and ovarian adenocarcinoma [43]. Variation in this region has been associated with Parkinson's disease (*MAPT*, *PLEKHM1*, *NSF*, *c17orf69*) progressive supranuclear palsy (*MAPT*), celiac disease (*WNT3*), bone mineral density (*CRHR1*) (NHGRI GWAS catalog) and intracranial volume [44]. Of the top hits for these phenotypes, SNP rs199533 in *NSF*, previously associated with Parkinson's disease [45] and rs9915547 associated with intracranial volume [44] were strongly associated with ovarian cancer (P<10^−9^ in *BRCA1/2* combined). Whether these phenotypes have shared causal variants in this locus remains to be elucidated. Further exploration of the functional relevance of the strongest hits in the 17q21.31 locus (P<10^−8^ in *BRCA1/2* combined) provided evidence that cis-regulatory variation alters expression of several genes at 17q21, including *PLEKHM1*, *c17orf69*, *ARHGAP27*, *MAPT*, *KANSL1* and *WNT3* [39], [41] (Table S9, Figure S12), suggesting that ovarian cancer risk may be associated with altered expression of one or more genes in this region.

Our analyses revealed that a second novel locus at 4q32.3 was also associated with ovarian cancer risk for *BRCA1* mutation carries (P<5×10^−8)^. However, we found no evidence of association for these SNPs with ovarian cancer risk for *BRCA2* mutation carriers using 8,211 CIMBA samples genotyped using the iCOGS array. Likewise, no evidence of association was found between rs4691139 at 4q32.3 and ovarian cancer risk in the general population based on data by OCAC data derived from 18,174 cases and 26,134 controls (odds ratio = 1.00, 95%CI:0.97–1.04, P = 0.76) [46]. The confidence intervals rule out a comparable effect to that found in *BRCA1* carriers. Therefore, our findings may represent a *BRCA1*-specific association with ovarian cancer risk, the first of its kind. The 4q32.2 region contains several members of the *TRIM* (Tripartite motif containing) gene family, *c4orf39* and *TMEM192*. *TRIM60*, *c4orf39* and *TMEM19*2 are expressed in normal ovarian epithelium and/or ovarian tumors (TCGA).

In summary, we have identified a novel locus at 1q32 associated with breast cancer risk for *BRCA1* mutation carriers, which was also associated with ER-negative breast cancer for *BRCA2* carriers and in the general population. A separate locus at 10q23.5 provided strong evidence of association with breast cancer risk for *BRCA1* carriers. We have also identified 2 novel loci associated with ovarian cancer for *BRCA1* mutation carriers. Of these, the 4q32.2 locus was associated with ovarian cancer risk for *BRCA1* carriers but not for *BRCA2* carriers or in the general population. Additional functional characterisation of the loci will further improve our understanding of the biology of breast and ovarian cancer development in *BRCA1* carriers. Taken together with other identified genetic modifiers, 10 loci are now known to be associated with breast cancer risk for *BRCA1* mutation carriers (1q32, 10q25.3, 19p13, 6q25.1, 12p11, *TOX3*, 2q35, *LSP1*, *RAD51L1* and *TERT* and seven loci are known to be associated with ovarian cancer risk for *BRCA1* mutation carriers (9p22, 8q24, 3q25, 17q21, 19p13 and 17q21.31, 4q32.3).

As *BRCA1* mutations confer high breast and ovarian cancer risks, the results from the present study, taken together with other identified genetic modifiers, demonstrate for the first time that they can result in large differences in the absolute risk of developing breast or ovarian cancer for *BRCA1* between genotypes. For example, the breast cancer lifetime risks for the 5% of *BRCA1* carriers at lowest risk are predicted to be 28–50% compared to 81–100% for the 5% at highest risk (Figure 3). Based on the distribution of ovarian cancer risk modifiers, the 5% of *BRCA1* mutation carriers at lowest risk will have a lifetime risk of developing ovarian cancer of 28% or lower whereas the 5% at highest risk will have a lifetime risk of 63% or higher. Similarly, the breast cancer risk by age 40 is predicted to be 4–9% for the 5% of *BRCA1* carriers at lowest risk compared to 20–49% for the 5% at highest risk, whereas the ovarian cancer risk at age 50 ranges from 3–7% for the 5% at lowest risk and from 18–47% for the 5% at highest risk. The risks at all ages for the 10% at highest or lowest risk of breast and ovarian cancer are predicted to be similar to those for the highest and lowest 5%. Thus, at least 20% of BRCA1 mutation carriers are predicted to have absolute risks of disease that are different from the average *BRCA1* carriers. These large differences in cancer risks may have practical implications for the clinical management of *BRCA1* mutation carriers, for example in deciding the timing of interventions. Such risks, in combination with other lifestyle and hormonal risk factors could be incorporated into cancer risk prediction algorithms for use by clinical genetics centers. These algorithms could then be used to inform the development of effective and consistent clinical recommendations for the clinical management of *BRCA1* mutation carriers.

## Supporting Information

Figure S1 Multidimensional scaling of stage 1 and stage 2 (genotyped on iCOGS) samples. Panel A: Graphical representation of the first two components, for the *BRCA1* carriers, for subgroups defined by the common 185delAG (c.68_69delAG) *BRCA1* Jewish founder mutation, the 5382insC (c.5266dupC) Eastern European founder mutation and Hapap individuals (CEU: European; ASI: Includes CHB and JPT populations; YRI: African). Panel B: Red dots represent the samples with >22% non-European ancestry, excluded from the analysis.
(PDF)

Figure S2 Genotyping cluster plots in the *BRCA1* samples for the key associated SNPs.
(PDF)

Figure S3 Quantile-quantile plot for the kinship adjusted score test statistic for stage 2 samples (1 degree of freedom χ^2^ trend test) for the associations with breast cancer (panel A) and ovarian cancer (panel B) risk for *BRCA1* mutation carriers. The y = x line corresponds to the expected distribution, under the hypothesis of no inflation. Inflation was estimated using the values of the lowest 90% test statistics.
(PDF)

Figure S4 P-values (on −log_10_ scale) by chromosomal position, for the associations of 31,812 *BRCA1* GWAS SNPs with breast (panel A) and ovarian (panel B) cancer risk for *BRCA1* mutation carriers in the combined stage 1 and stage 2 samples. Blue lines correspond to a P-value of 10^−5^; red lines correspond to P-value 5×10^−8^.
(PDF)

Figure S5 Forest plots of the associations by country of residence of *BRCA1* mutation carriers in the combined stage 1, stage 2 and stage 3 samples for SNPs found to be associated with breast and ovarian cancer risk for *BRCA1* mutation carriers. Squares indicate the country specific, per-allele HR estimates for the SNPs. The area of the square is proportional to the inverse of the variance of the estimate. Horizontal lines indicate 95% confidence intervals. There was some evidence of heterogeneity in country-specific HR estimates for the rs2290854 and rs6682208 SNP (P = 0.04 and 0.02 respectively, Figure S3), but after accounting for opposite effects of these SNPs in Finland/Denmark, there was no evidence of heterogeneity. There was some evidence of heterogeneity in the country-specific HRs for rs17631303 (P-het = 0.004, df = 19) but this was no longer present after excluding one country (Poland, P-het = 0.12, df = 18), or when restricting analyses to Stage 1 and 2 samples only (P-het = 0.09, df = 19). There was no evidence of heterogeneity for correlated SNP rs183211 (P-het = 0.10). There was no evidence of hereterogeneity in the country-specific HRs for any of the other SNPs (P>0.68).
(PDF)

Figure S6 *MDM4* regional association plot using *BRCA1* stage 1 and stage 2 samples. P-values for association (−log_10_ scale) with breast cancer risk for *BRCA1* mutation carriers for genotyped SNPs (diamond symbols ◊) and SNPs imputed from the 1000 genomes project data (square symbols □), by position (hg18) on chromosome 1. Red gradient represents r^2^ value with the most significant genotyped SNP rs4951407. The blue peaks represent recombination rate in the region.
(PDF)

Figure S7 *TCF7L2* regional association plot using *BRCA1* stage 1 and stage 2 samples. P-values for association (−log_10_ scale) with breast cancer risk for *BRCA1* mutation carriers for genotyped SNPs (diamond symbols ◊) and SNPs imputed from the 1000 genomes project data (square symbols □), by position (hg18) on chromosome 1. Missing genotypes were replaced by imputed results. Red gradient represents r^2^ value with the most significant genotyped SNP rs11196174. The blue peaks represent recombination rate in the region.
(PDF)

Figure S8 Linkage disequilibrium patterns between the SNPs in the novel (17q21.31) and previously identified regions on 17q21. SNPs in the novel region are uncorrelated with SNPs in the 43.3–44.3 Mb region (positions according to hg build 36.3).
(PDF)

Figure S9 4q32.3 regional association plot using *BRCA1* stage 1 and stage 2 samples. P-values for association (−log_10_ scale) with ovarian cancer risk for *BRCA1* mutation carriers for genotyped SNPs (diamond symbols ◊) and imputed SNPs from the 1000 genomes project data (square symbols □), by position (hg18) on chromosome 1. Red gradient represents r^2^ value with the most significant genotyped SNP rs4691139. Blue peaks represent recombination rate in the region.
(PDF)

Figure S10 Combined Hazard Ratios (HR) for breast and ovarian cancer for *BRCA1* mutation carriers. (A) HR for Breast Cancer based on 10 loci associated with breast cancer risk for *BRCA1* mutation carriers. (B) Ovarian Cancer based on 7 loci associated with ovarian cancer risk for *BRCA1* mutation carriers. All HRs computed relative to the lowest risk category. The Y-axes translate the combined HRs into absolute risks of developing breast or ovarian cancer by age 80. The absolute risks and HRs at different percentiles of the combined genotype distribution are also marked. The combined HRs were obtained under the assumption that the loci interact multiplicatively.
(PDF)

Figure S11 Cis-eQTL and allelic expression (AE) analyses at *MDM4* locus. A) Cis-eQTLs for SNPs at *MDM4* locus using expression data from primary human osteoblasts (HOb). B) AE mapping for cis-regulatory variation in *MDM4* locus using primary skin fibroblasts. Coordinates (hg18) for locus shown on top; blue tracks indicate the −log_10_(P value) of the association across all SNPs tested. The location of transcripts in this region is shown below.
(PDF)

Figure S12 Cis-eQTL and allelic expression (AE) analyses at chr17q21.31 locus. (Upper panel) Cis-eQTLs for SNPs at c17orf69 locus using expression data from primary human osteoblasts. Allelic expression mapping for cis-regulatory variation in *KANSL1* (middle panel) and *WNT3* loci (lower panel) using a CEU population panel of lymphoblastoid cells. Coordinates (hg18) for loci are shown on top; blue tracks indicate the −log_10_(P value) of the association across all SNPs tested. The location of transcripts in these regions are shown.
(PDF)

Table S1 Affected and unaffected *BRCA1* mutation carriers by study country in the breast and ovarian cancer analysis used in SNP selection for the iCOGS array.
(DOCX)

Table S2 Origin of *BRCA1* samples by Country and Stage used in the current analysis.
(DOCX)

Table S3 Sample and SNP quality control summary.
(DOCX)

Table S4 Associations with breast cancer risk for *BRCA1* mutation carriers, for known breast cancer susceptibility variants.
(DOCX)

Table S5 Associations with *BRCA1* breast or ovarian cancer risk for SNPs genotyped at stages 1, 2, and 3.
(DOCX)

Table S6 Analysis of breast cancer associations by *BRCA1* mutation class.
(DOCX)

Table S7 Associations with Breast Cancer ER status in *BRCA1* carriers for SNPs genotyped in stages 1–3.
(DOCX)

Table S8 Imputed SNPs at the novel 17q21 region with P-values less than the most significant genotyped SNP (rs169201).
(DOCX)

Table S9 SNPs associated (P<1×10^−5^) with local expression and Allelic Imbalance.
(DOCX)

Text S1 Supplementary Methods.
(DOCX)

## References

[1] Antoniou A, Pharoah PD, Narod S, Risch HA, Eyfjord JE, et al (2003) Average risks of breast and ovarian cancer associated with *BRCA1* or *BRCA2* mutations detected in case series unselected for family history: a combined analysis of 22 studies. Am J Hum Genet 72: 1117–1130. 12677558 10.1086/375033 PMC1180265

[2] Antoniou AC, Chenevix-Trench G (2010) Common genetic variants and cancer risk in Mendelian cancer syndromes. Curr Opin Genet Dev 20: 299–307 S0959-437X(10)00044-4 [pii];10.1016/j.gde.2010.03.010 [doi]. 20399636 10.1016/j.gde.2010.03.010

[3] Begg CB, Haile RW, Borg A, Malone KE, Concannon P, et al (2008) Variation of breast cancer risk among *BRCA1*/2 carriers. JAMA 299: 194–201. 18182601 10.1001/jama.2007.55-a PMC2714486

[4] Simchoni S, Friedman E, Kaufman B, Gershoni-Baruch R, Orr-Urtreger A, et al (2006) Familial clustering of site-specific cancer risks associated with *BRCA1* and *BRCA2* mutations in the Ashkenazi Jewish population. Proc Natl Acad Sci U S A 103: 3770–3774. 16537453 10.1073/pnas.0511301103 PMC1450152

[5] Couch FJ, Gaudet MM, Antoniou AC, Ramus SJ, Kuchenbaecker KB, et al (2012) Common cariants at the 19p13.1 and *ZNF365* loci are associated with ER subtypes of breast cancer and ovarian cancer risk in *BRCA1* and BRCA2 mutation carriers. Cancer Epidemiol Biomarkers Prev 21: 645–657 1055-9965.EPI-11-0888 [pii];10.1158/1055-9965.EPI-11-0888 [doi]. 22351618 10.1158/1055-9965.EPI-11-0888 PMC3319317

[6] Ramus SJ, Kartsonaki C, Gayther SA, Pharoah PD, Sinilnikova OM, et al (2010) Genetic variation at 9p22.2 and ovarian cancer risk for *BRCA1* and BRCA2 mutation carriers. J Natl Cancer Inst 103: 105–116 djq494 [pii];10.1093/jnci/djq494 [doi]. 21169536 10.1093/jnci/djq494 PMC3107565

[7] Ramus SJ, Antoniou AC, Kuchenbaecker KB, Soucy P, Beesley J, et al (2012) Ovarian cancer susceptibility alleles and risk of ovarian cancer in *BRCA1* and *BRCA2* mutation carriers. Hum Mutat 33: 690–702 10.1002/humu.22025 [doi]. 22253144 10.1002/humu.22025 PMC3458423

[8] Antoniou AC, Spurdle AB, Sinilnikova OM, Healey S, Pooley KA, et al (2008) Common breast cancer-predisposition alleles are associated with breast cancer risk in *BRCA1* and *BRCA*2 mutation carriers. Am J Hum Genet 82: 937–948. 18355772 10.1016/j.ajhg.2008.02.008 PMC2427217

[9] Antoniou AC, Sinilnikova OM, McGuffog L, Healey S, Nevanlinna H, et al (2009) Common variants in *LSP1*, 2q35 and 8q24 and breast cancer risk for *BRCA1* and BRCA2 mutation carriers. Hum Mol Genet 18: 4442–4456 ddp372 [pii];10.1093/hmg/ddp372 [doi]. 19656774 10.1093/hmg/ddp372 PMC2782243

[10] Antoniou AC, Kartsonaki C, Sinilnikova OM, Soucy P, McGuffog L, et al (2011) Common alleles at 6q25.1 and 1p11.2 are associated with breast cancer risk for *BRCA1* and *BRCA2* mutation carriers. Hum Mol Genet 20: 3304–3321 ddr226 [pii];10.1093/hmg/ddr226 [doi]. 21593217 10.1093/hmg/ddr226 PMC3652640

[11] Antoniou AC, Kuchenbaecker KB, Soucy P, Beesley J, Chen X, et al (2012) Common variants at 12p11, 12q24, 9p21, 9q31.2 and in *ZNF365* are associated with breast cancer risk for *BRCA1* and/or *BRCA2* mutation carriers. Breast Cancer Res 14: R33 bcr3121 [pii];10.1186/bcr3121 [doi]. 22348646 10.1186/bcr3121 PMC3496151

[12] Antoniou AC, Wang X, Fredericksen ZS, McGuffog L, Tarrell R, et al (2010) A locus on 19p13 modifies risk of breast cancer in *BRCA1* mutation carriers and is associated with hormone receptor-negative breast cancer in the general population. Nat Genet 42: 885–892 ng.669 [pii];10.1038/ng.669 [doi]. 20852631 10.1038/ng.669 PMC3130795

[13] Stevens KN, Vachon CM, Lee AM, Slager S, Lesnick T, et al (2011) Common breast cancer susceptibility loci are associated with triple negative breast cancer. Cancer Res 0008-5472.CAN-11-1266 [pii];10.1158/0008-5472.CAN-11-1266 [doi]. 10.1158/0008-5472.CAN-11-1266 PMC3327299 21844186

[14] Gaudet MM, Kuchenbaecker KB, Vijai J, Klein RJ, Kirchhoff T, et al (2013) Identification of a BRCA2-specific Modifier Locus at 6p24 Related to Breast Cancer Risk. PLoS Genet 9: e1003173 doi:10.1371/journal.pgen.1003173. 23544012 10.1371/journal.pgen.1003173 PMC3609647

[15] Robertson A, Hill WG (1984) Deviations from Hardy-Weinberg proportions: sampling variances and use in estimation of inbreeding coefficients. Genetics 107: 703–718. 6745643 10.1093/genetics/107.4.703 PMC1202385

[16] Antoniou AC, Goldgar DE, Andrieu N, Chang-Claude J, Brohet R, et al (2005) A weighted cohort approach for analysing factors modifying disease risks in carriers of high-risk susceptibility genes. Genet Epidemiol 29: 1–11. 15880399 10.1002/gepi.20074

[17] Barnes DR, Lee A, Easton DF, Antoniou AC (2012) Evaluation of association methods for analysing modifiers of disease risk in carriers of high-risk mutations. Genet Epidemiol 36: 274–291 10.1002/gepi.21620 [doi]. 22714938 10.1002/gepi.21620

[18] Antoniou AC, Sinilnikova OM, Simard J, Leone M, Dumont M, et al (2007) RAD51 135G→C modifies breast cancer risk among *BRCA2* mutation carriers: results from a combined analysis of 19 studies. Am J Hum Genet 81: 1186–1200. 17999359 10.1086/522611 PMC2276351

[19] Amin N, van Duijn CM, Aulchenko YS (2007) A genomic background based method for association analysis in related individuals. PLoS ONE 2: e1274 doi:10.1371/journal.pone.0001274. 18060068 10.1371/journal.pone.0001274 PMC2093991

[20] Leutenegger AL, Prum B, Genin E, Verny C, Lemainque A, et al (2003) Estimation of the inbreeding coefficient through use of genomic data. Am J Hum Genet 73: 516–523 10.1086/378207 [doi];S0002-9297(07)62015-1 [pii]. 12900793 10.1086/378207 PMC1180677

[21] Boos DD (1992) On generalised score tests. American Statistician 46: 327–333.

[22] Wellcome Trust Case Control Consortium (2007) Genome-wide association study of 14,000 cases of seven common diseases and 3,000 shared controls. Nature 447: 661–678 nature05911 [pii];10.1038/nature05911 [doi]. 17554300 10.1038/nature05911 PMC2719288

[23] Aulchenko YS, Ripke S, Isaacs A, van Duijn CM (2007) GenABEL: an R library for genome-wide association analysis. Bioinformatics 23: 1294–1296 btm108 [pii];10.1093/bioinformatics/btm108 [doi]. 17384015 10.1093/bioinformatics/btm108

[24] Lange K, Weeks D, Boehnke M (1988) Programs for pedigree analysis: MENDEL, FISHER, and dGENE. Genet Epidemiol 5: 471–472. 3061869 10.1002/gepi.1370050611

[25] Antoniou AC, Cunningham AP, Peto J, Evans DG, Lalloo F, et al (2008) The BOADICEA model of genetic susceptibility to breast and ovarian cancers: updates and extensions. Br J Cancer 98: 1457–1466. 18349832 10.1038/sj.bjc.6604305 PMC2361716

[26] Mulligan AM, Couch FJ, Barrowdale D, Domchek SM, Eccles D, et al (2011) Common breast cancer susceptibility alleles are associated with tumor subtypes in *BRCA1* and *BRCA2* mutation carriers: results from the Consortium of Investigators of Modifiers of *BRCA1*/2. Breast Cancer Res 13: R110 bcr3052 [pii];10.1186/bcr3052 [doi]. 22053997 10.1186/bcr3052 PMC3326552

[27] Howie B, Marchini J, Stephens M (2011) Genotype imputation with thousands of genomes. G3 (Bethesda) 1: 457–470 10.1534/g3.111.001198 [doi];GGG_001198 [pii]. 22384356 10.1534/g3.111.001198 PMC3276165

[28] Antoniou AC, Beesley J, McGuffog L, Sinilnikova OM, Healey S, et al (2010) Common Breast Cancer Susceptibility Alleles and the Risk of Breast Cancer for *BRCA1* and *BRCA2* Mutation Carriers: Implications for Risk Prediction. Cancer Res 70: 9742–9754 0008-5472.CAN-10-1907 [pii];10.1158/0008-5472.CAN-10-1907 [doi]. 21118973 10.1158/0008-5472.CAN-10-1907 PMC2999830

[29] Mavaddat N, Barrowdale D, Andrulis IL, Domchek SM, Eccles D, et al (2012) Pathology of breast and ovarian cancers among *BRCA1* and *BRCA2* mutation carriers: results from the Consortium of Investigators of Modifiers of *BRCA1/2* (CIMBA). Cancer Epidemiol Biomarkers Prev 21: 134–147 1055-9965.EPI-11-0775 [pii];10.1158/1055-9965.EPI-11-0775 [doi]. 22144499 10.1158/1055-9965.EPI-11-0775 PMC3272407

[30] Goode EL, Chenevix-Trench G, Song H, Ramus SJ, Notaridou M, et al (2010) A genome-wide association study identifies susceptibility loci for ovarian cancer at 2q31 and 8q24. Nat Genet 42: 874–879 ng.668 [pii];10.1038/ng.668 [doi]. 20852632 10.1038/ng.668 PMC3020231

[31] Bojesen S, Pooley KA, Johnatty SE, Beesley J, Michailidou K, et al (2012) Multiple independent TERT variants associated with telomere length and risks of breast and ovarian cancer. Nat Genet In Press. 10.1038/ng.2566 PMC3670748 23535731

[32] Michailidou K, Hall P, Gonzalez-Neira A, Ghoussaini M, Dennis J, et al (2012) Large-scale genotyping identifies 41 new loci associated with breast cancer risk. Nat Genet In Press. 10.1038/ng.2563 PMC3771688 23535729

[33] Sladek R, Rocheleau G, Rung J, Dina C, Shen L, et al (2007) A genome-wide association study identifies novel risk loci for type 2 diabetes. Nature 445: 881–885 nature05616 [pii];10.1038/nature05616 [doi]. 17293876 10.1038/nature05616

[34] Garcia-Closas M, Couch FJ, Lindstrom S, Michailidou K, Schmidt MK, et al (2012) Genome-wide association studies identify four ER-negative specific breast cancer risk loci. Nat Genet In Press. 10.1038/ng.2561 PMC3771695 23535733

[35] Atwal GS, Kirchhoff T, Bond EE, Montagna M, Menin C, et al (2009) Altered tumor formation and evolutionary selection of genetic variants in the human *MDM4* oncogene. Proc Natl Acad Sci U S A 106: 10236–10241 0901298106 [pii];10.1073/pnas.0901298106 [doi]. 19497887 10.1073/pnas.0901298106 PMC2700939

[36] Curtis C, Shah SP, Chin SF, Turashvili G, Rueda OM, et al (2012) The genomic and transcriptomic architecture of 2,000 breast tumours reveals novel subgroups. Nature 486: 346–352 nature10983 [pii];10.1038/nature10983 [doi]. 22522925 10.1038/nature10983 PMC3440846

[37] Laurie NA, Donovan SL, Shih CS, Zhang J, Mills N, et al (2006) Inactivation of the p53 pathway in retinoblastoma. Nature 444: 61–66 nature05194 [pii];10.1038/nature05194 [doi]. 17080083 10.1038/nature05194

[38] Wade M, Wahl GM (2009) Targeting Mdm2 and Mdmx in cancer therapy: better living through medicinal chemistry? Mol Cancer Res 7: 1–11 7/1/1 [pii];10.1158/1541-7786.MCR-08-0423 [doi]. 19147532 10.1158/1541-7786.MCR-08-0423 PMC2629357

[39] Fairfax BP, Makino S, Radhakrishnan J, Plant K, Leslie S, et al (2012) Genetics of gene expression in primary immune cells identifies cell type-specific master regulators and roles of HLA alleles. Nat Genet 44: 501–510 ng.2205 [pii];10.1038/ng.2205 [doi]. 10.1038/ng.2205 PMC3437404 22446964

[40] Ge B, Pokholok DK, Kwan T, Grundberg E, Morcos L, et al (2009) Global patterns of cis variation in human cells revealed by high-density allelic expression analysis. Nat Genet 41: 1216–1222 ng.473 [pii];10.1038/ng.473 [doi]. 19838192 10.1038/ng.473

[41] Grundberg E, Adoue V, Kwan T, Ge B, Duan QL, et al (2011) Global analysis of the impact of environmental perturbation on cis-regulation of gene expression. PLoS Genet 7: e1001279 doi:10.1371/journal.pgen.1001279. 21283786 10.1371/journal.pgen.1001279 PMC3024267

[42] Stefansson H, Helgason A, Thorleifsson G, Steinthorsdottir V, Masson G, et al (2005) A common inversion under selection in Europeans. Nat Genet 37: 129–137 ng1508 [pii];10.1038/ng1508 [doi]. 15654335 10.1038/ng1508

[43] Bowen NJ, Walker LD, Matyunina LV, Logani S, Totten KA, et al (2009) Gene expression profiling supports the hypothesis that human ovarian surface epithelia are multipotent and capable of serving as ovarian cancer initiating cells. BMC Med Genomics 2: 71 1755-8794-2-71 [pii];10.1186/1755-8794-2-71 [doi]. 20040092 10.1186/1755-8794-2-71 PMC2806370

[44] Ikram MA, Fornage M, Smith AV, Seshadri S, Schmidt R, et al (2012) Common variants at 6q22 and 17q21 are associated with intracranial volume. Nat Genet 44: 539–544 ng.2245 [pii];10.1038/ng.2245 [doi]. 22504418 10.1038/ng.2245 PMC3618290

[45] Simon-Sanchez J, Schulte C, Bras JM, Sharma M, Gibbs JR, et al (2009) Genome-wide association study reveals genetic risk underlying Parkinson's disease. Nat Genet 41: 1308–1312 ng.487 [pii];10.1038/ng.487 [doi]. 19915575 10.1038/ng.487 PMC2787725

[46] Pharoah P, Tsai YY, Ramus S, Phelan C, Goode EL, et al (2012) GWAS meta-analysis and replication identifies three new susceptibility loci for ovarian cancer. Nat Genet In Press. 10.1038/ng.2564 PMC3693183 23535730

