# Supplementary figures and images for: Genome-Wide Association Study in BRCA1 Mutation Carriers Identifies Novel Loci Associated with Breast and Ovarian Cancer Risk
Source: PLoS Genet. 2013 Mar 27;9(3):e1003212. doi: 10.1371/journal.pgen.1003212 (PMC3609646; doi:10.1371/journal.pgen.1003212)

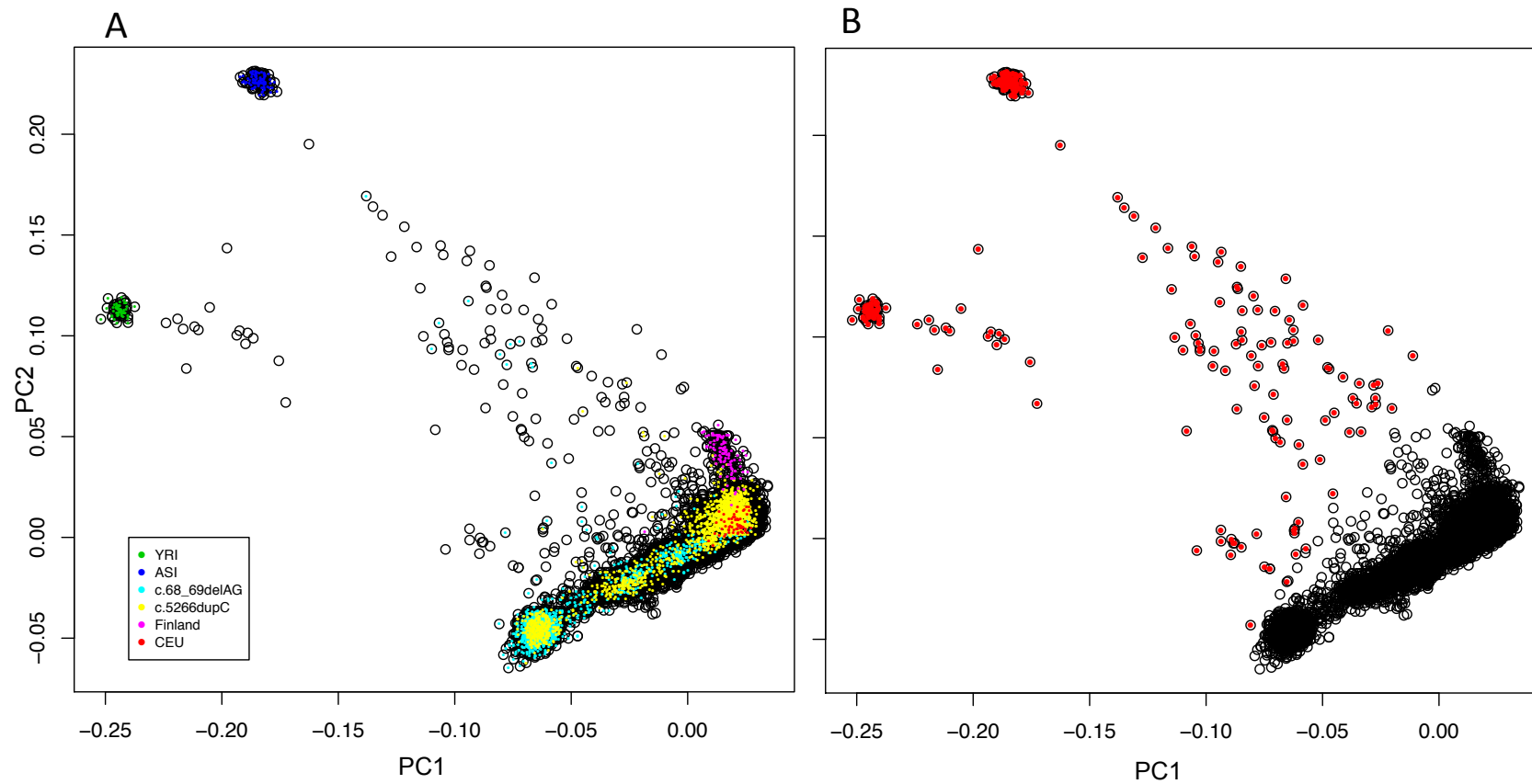

Supplementary Figure 1

Supplement: Figure S1 — Multidimensional scaling of stage 1 and stage 2 (genotyped on iCOGS) samples. Panel A: Graphical representation of the first two components, for the BRCA1 carriers, for subgroups defined by the common 185delAG (c.68_69delAG) BRCA1 Jewish founder mutation, the 5382insC (c.5266dupC) Eastern European founder mutation and Hapap individuals (CEU: European; ASI: Includes CHB and JPT populations; YRI: African). Panel B: Red dots represent the samples with >22% non-European ancestry, excluded from the analysis. (PDF) [file pgen.1003212.s001.pdf]

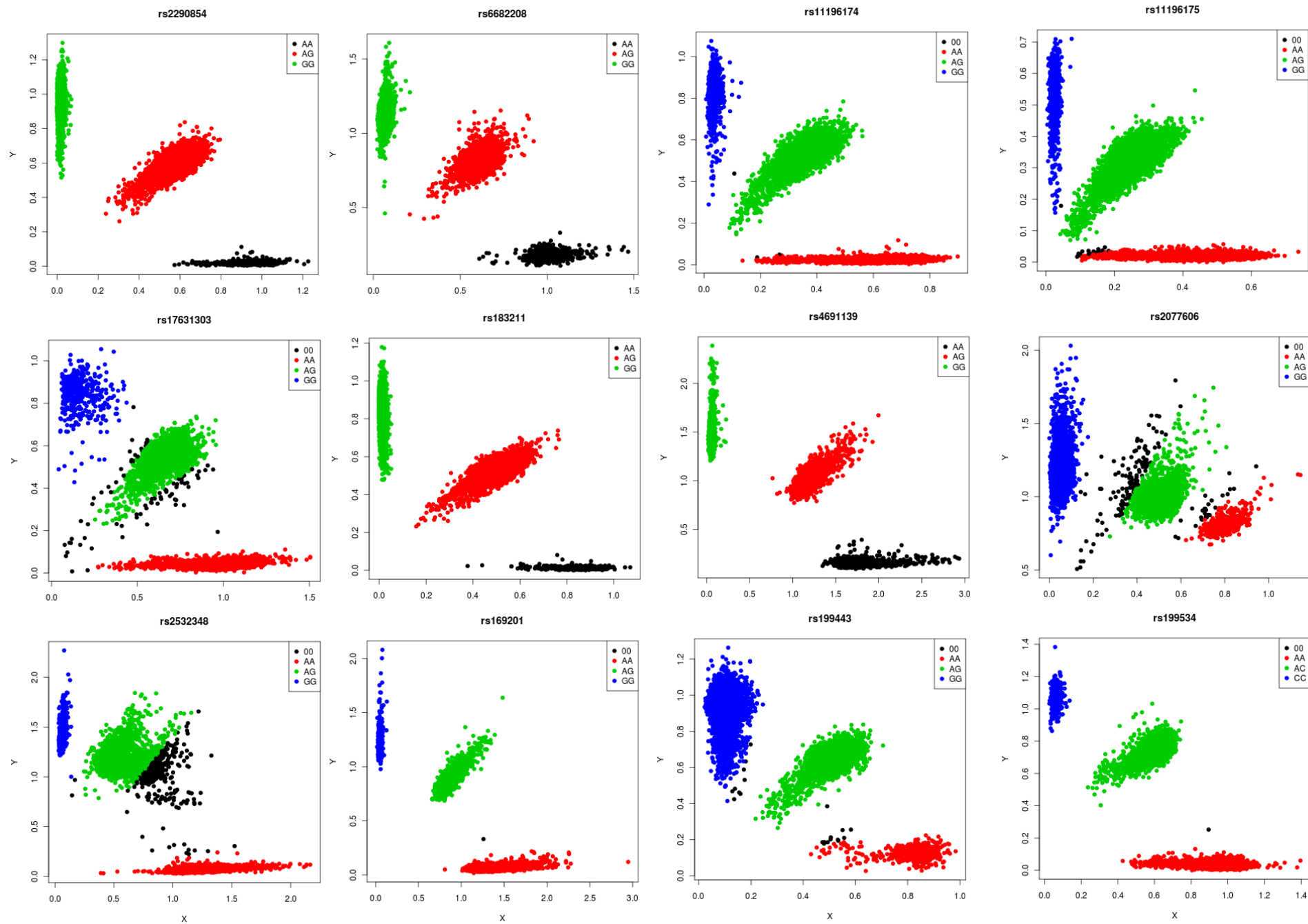

Supplementary Figure 2

Supplement: Figure S2 — Genotyping cluster plots in the BRCA1 samples for the key associated SNPs. (PDF) [file pgen.1003212.s002.pdf]

A. Breast Cancer

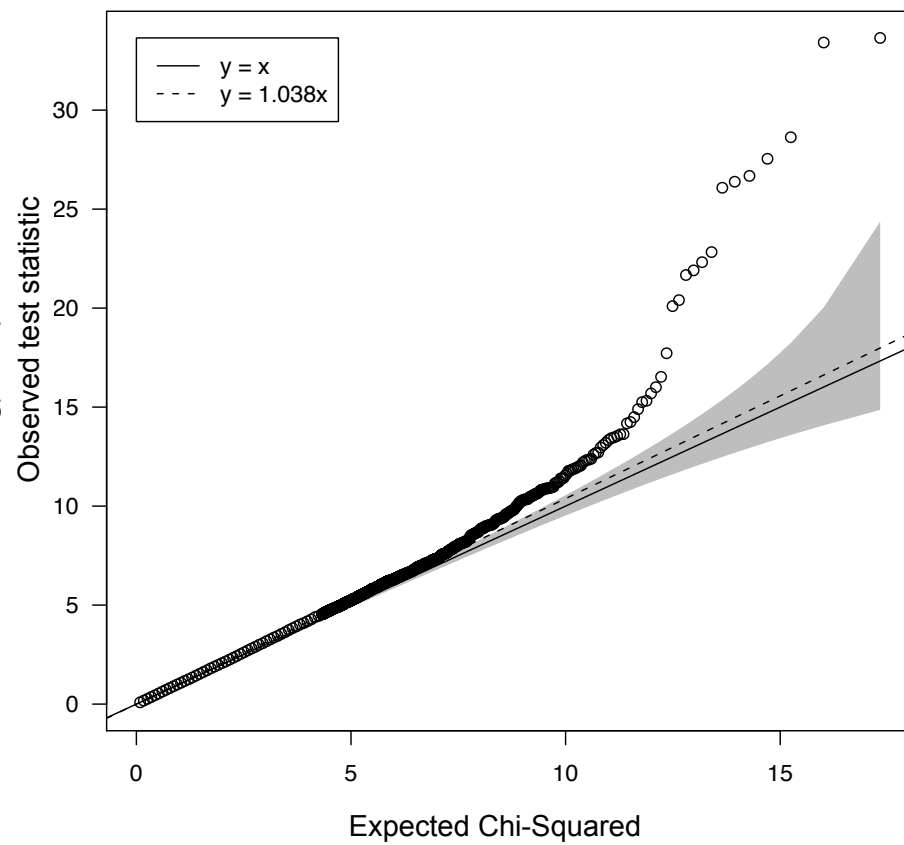

B. Ovarian Cancer

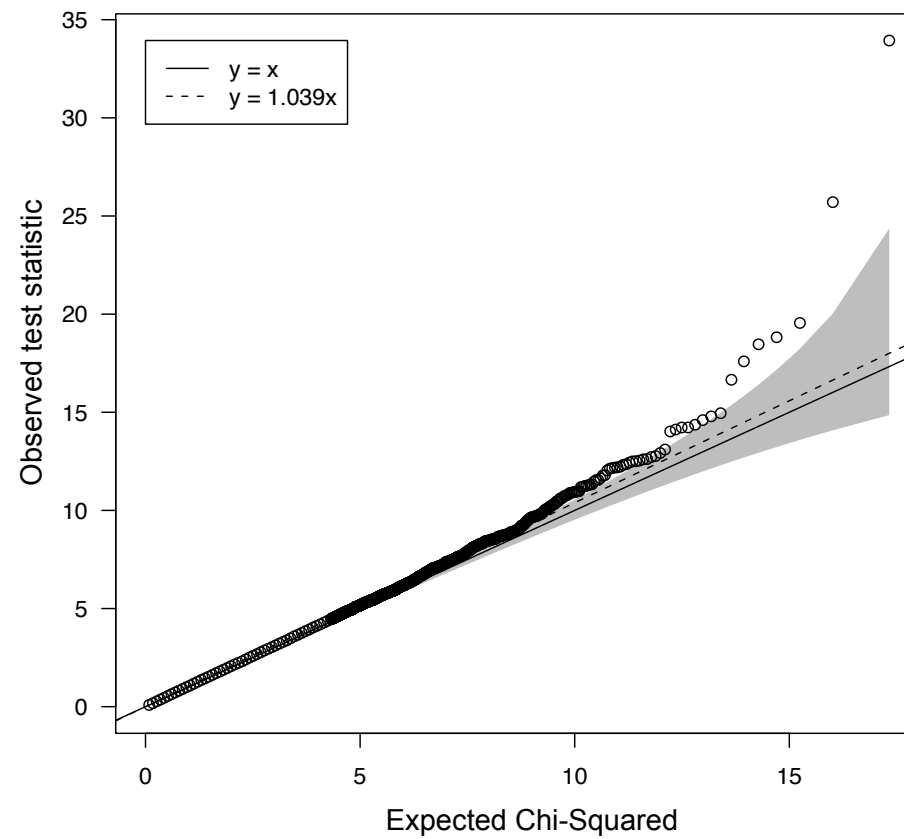

Supplement: Figure S3 — Quantile-quantile plot for the kinship adjusted score test statistic for stage 2 samples (1 degree of freedom χ2 trend test) for the associations with breast cancer (panel A) and ovarian cancer (panel B) risk for BRCA1 mutation carriers. The y = x line corresponds to the expected distribution, under the hypothesis of no inflation. Inflation was estimated using the values of the lowest 90% test statistics. (PDF) [file pgen.1003212.s003.pdf]

**A. Breast Cancer**

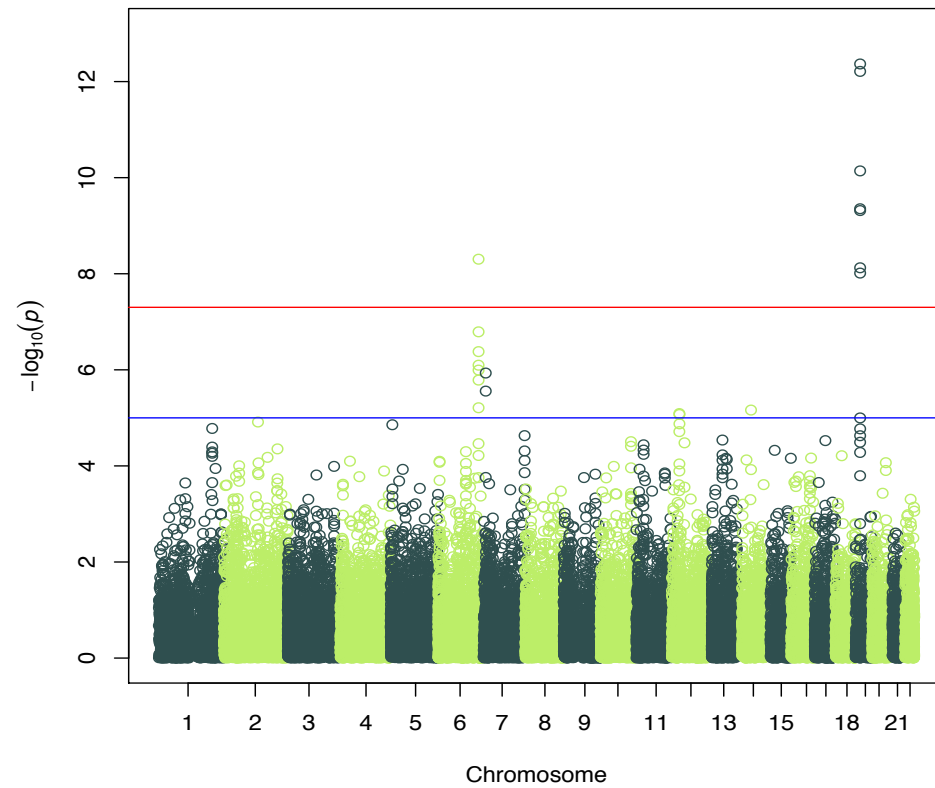

**B. Ovarian Cancer**

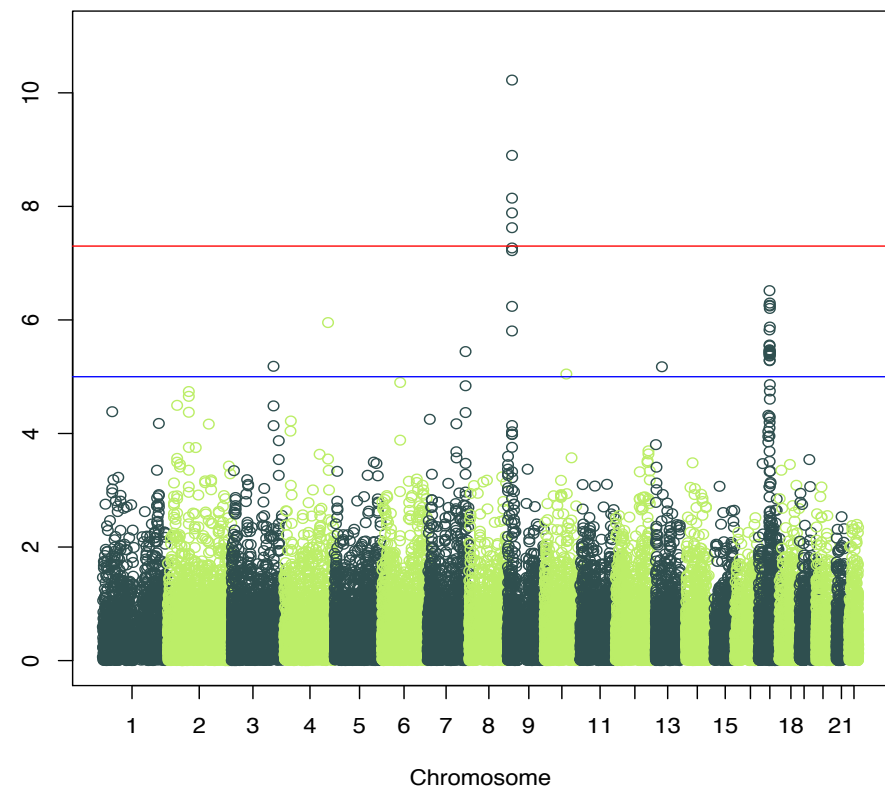

Supplement: Figure S4 — P-values (on −log10 scale) by chromosomal position, for the associations of 31,812 BRCA1 GWAS SNPs with breast (panel A) and ovarian (panel B) cancer risk for BRCA1 mutation carriers in the combined stage 1 and stage 2 samples. Blue lines correspond to a P-value of 10−5; red lines correspond to P-value 5×10^−8^. (PDF) [file pgen.1003212.s004.pdf]

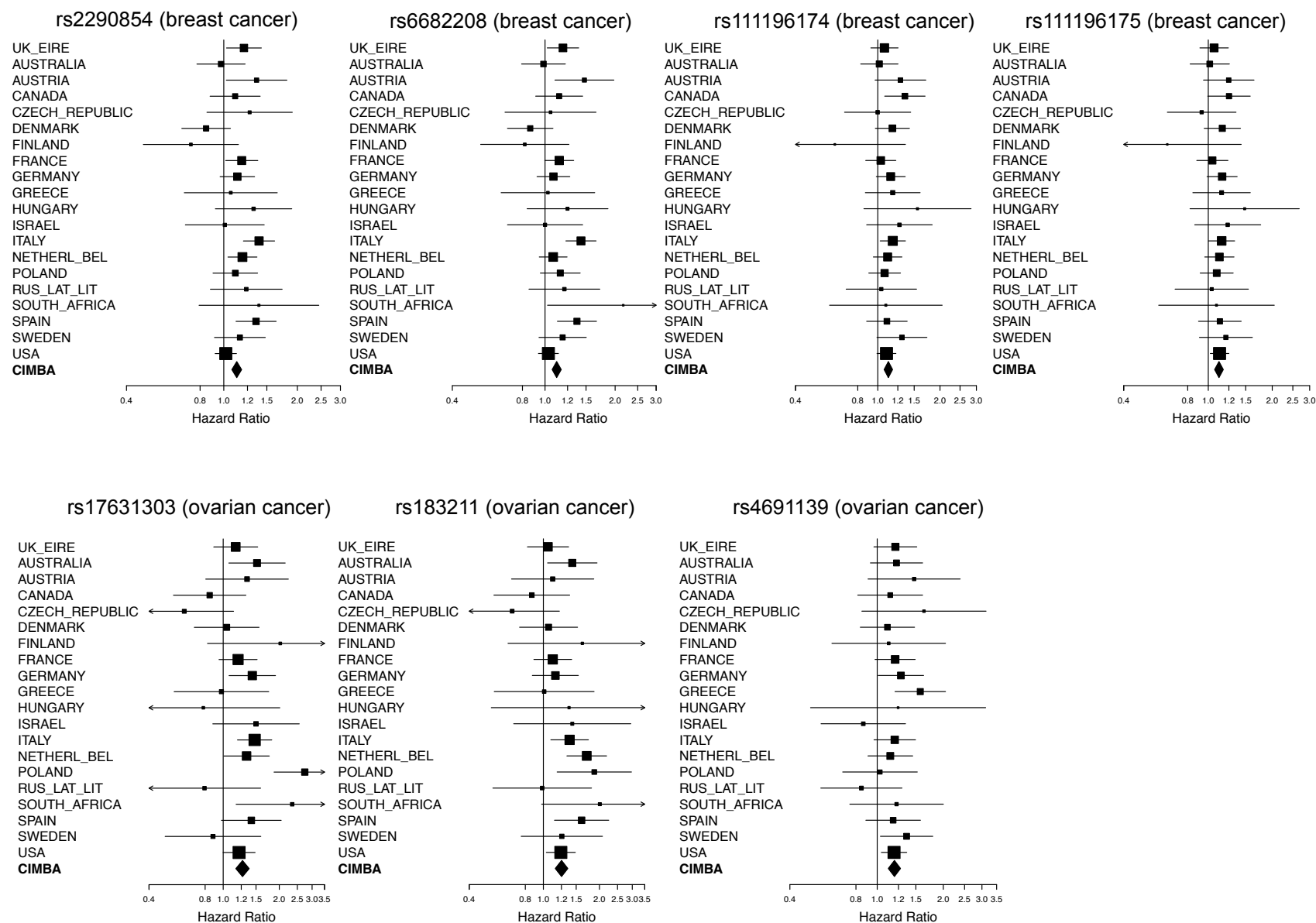

Supplementary Figure 5

Supplement: Figure S5 — Forest plots of the associations by country of residence of BRCA1 mutation carriers in the combined stage 1, stage 2 and stage 3 samples for SNPs found to be associated with breast and ovarian cancer risk for BRCA1 mutation carriers. Squares indicate the country specific, per-allele HR estimates for the SNPs. The area of the square is proportional to the inverse of the variance of the estimate. Horizontal lines indicate 95% confidence intervals. There was some evidence of heterogeneity in country-specific HR estimates for the rs2290854 and rs6682208 SNP (P = 0.04 and 0.02 respectively, Figure S3), but after accounting for opposite effects of these SNPs in Finland/Denmark, there was no evidence of heterogeneity. There was some evidence of heterogeneity in the country-specific HRs for rs17631303 (P-het = 0.004, df = 19) but this was no longer present after excluding one country (Poland, P-het = 0.12, df = 18), or when restricting analyses to Stage 1 and 2 samples only (P-het = 0.09, df = 19). There was no evidence of heterogeneity for correlated SNP rs183211 (P-het = 0.10). There was no evidence of hereterogeneity in the country-specific HRs for any of the other SNPs (P>0.68). (PDF) [file pgen.1003212.s005.pdf]

rs4951407 ( CEU )

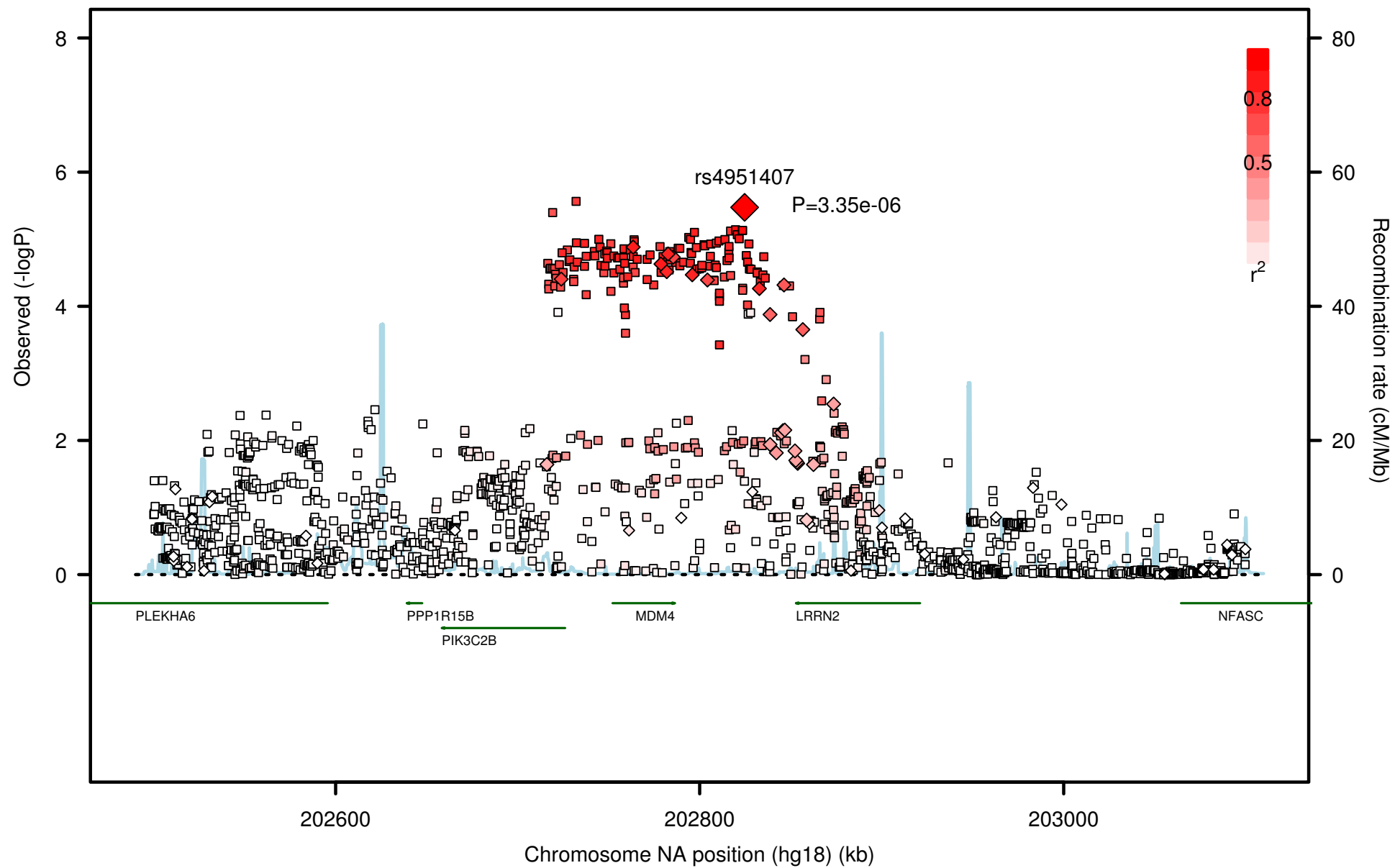

Supplementary Figure 6

Supplement: Figure S6 — MDM4 regional association plot using BRCA1 stage 1 and stage 2 samples. P-values for association (−log10 scale) with breast cancer risk for BRCA1 mutation carriers for genotyped SNPs (diamond symbols ◊) and SNPs imputed from the 1000 genomes project data (square symbols □), by position (hg18) on chromosome 1. Red gradient represents r2 value with the most significant genotyped SNP rs4951407. The blue peaks represent recombination rate in the region. (PDF) [file pgen.1003212.s006.pdf]

# rs11196174 ( CEU )

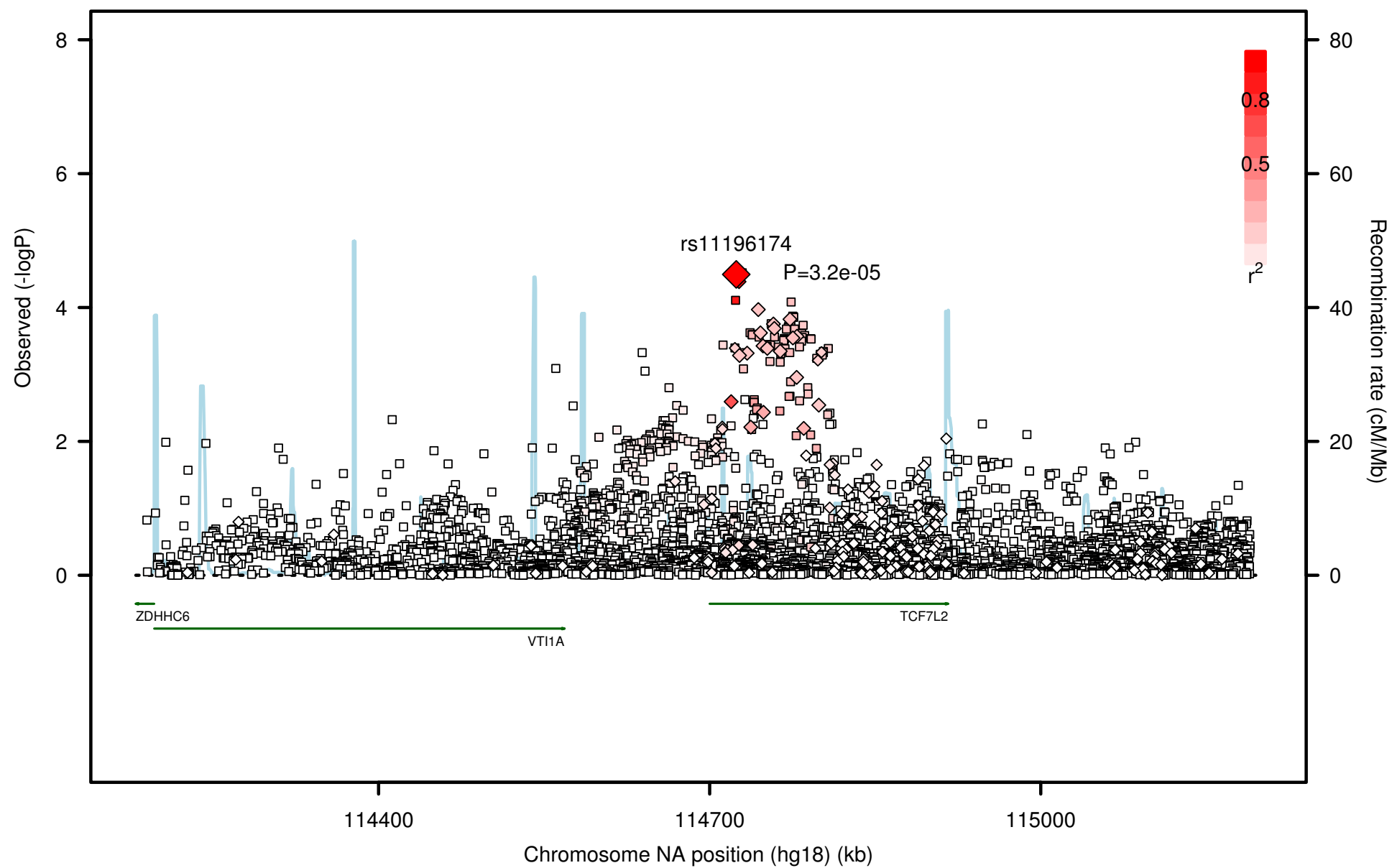

Supplementary Figure 7

Supplement: Figure S7 — TCF7L2 regional association plot using BRCA1 stage 1 and stage 2 samples. P-values for association (−log10 scale) with breast cancer risk for BRCA1 mutation carriers for genotyped SNPs (diamond symbols ◊) and SNPs imputed from the 1000 genomes project data (square symbols □), by position (hg18) on chromosome 1. Missing genotypes were replaced by imputed results. Red gradient represents r2 value with the most significant genotyped SNP rs11196174. The blue peaks represent recombination rate in the region. (PDF) [file pgen.1003212.s007.pdf]

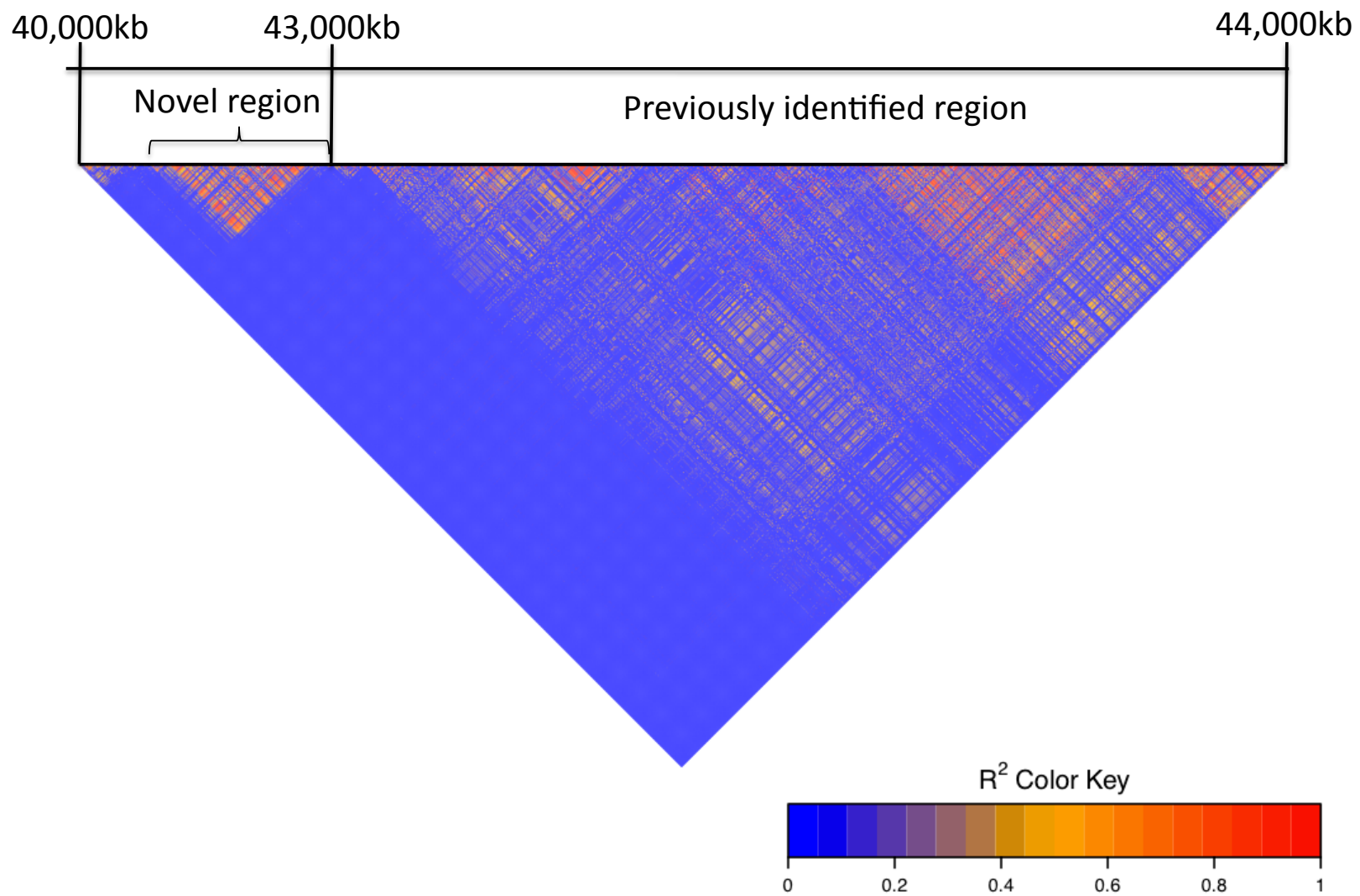

Supplementary Figure 8

Supplement: Figure S8 — Linkage disequilibrium patterns between the SNPs in the novel (17q21.31) and previously identified regions on 17q21. SNPs in the novel region are uncorrelated with SNPs in the 43.3–44.3 Mb region (positions according to hg build 36.3). (PDF) [file pgen.1003212.s008.pdf]

# rs4691139 ( CEU )

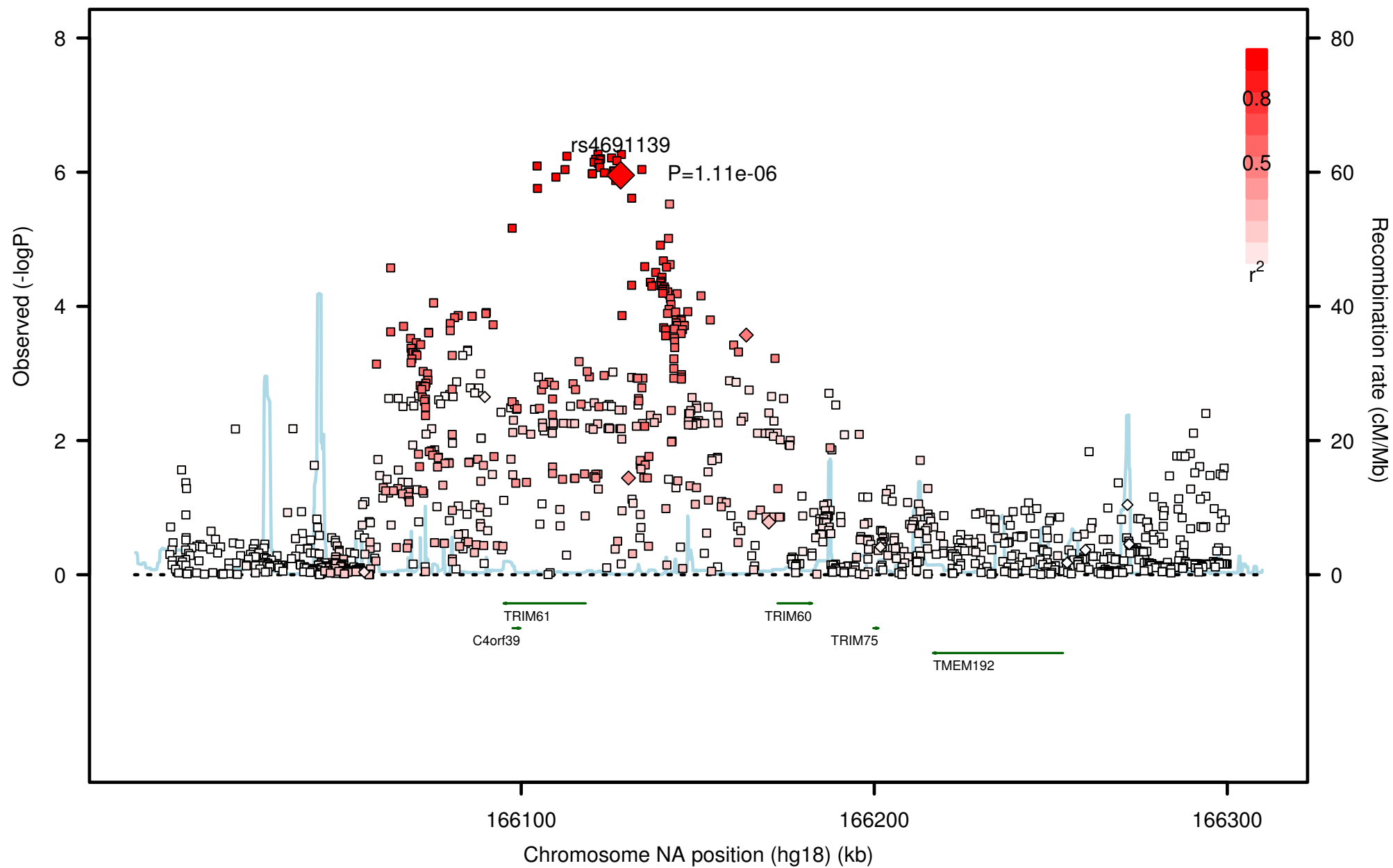

Supplementary Figure 9

Supplement: Figure S9 — 4q32.3 regional association plot using BRCA1 stage 1 and stage 2 samples. P-values for association (−log10 scale) with ovarian cancer risk for BRCA1 mutation carriers for genotyped SNPs (diamond symbols ◊) and imputed SNPs from the 1000 genomes project data (square symbols □), by position (hg18) on chromosome 1. Red gradient represents r2 value with the most significant genotyped SNP rs4691139. Blue peaks represent recombination rate in the region. (PDF) [file pgen.1003212.s009.pdf]

**A. Breast Cancer**

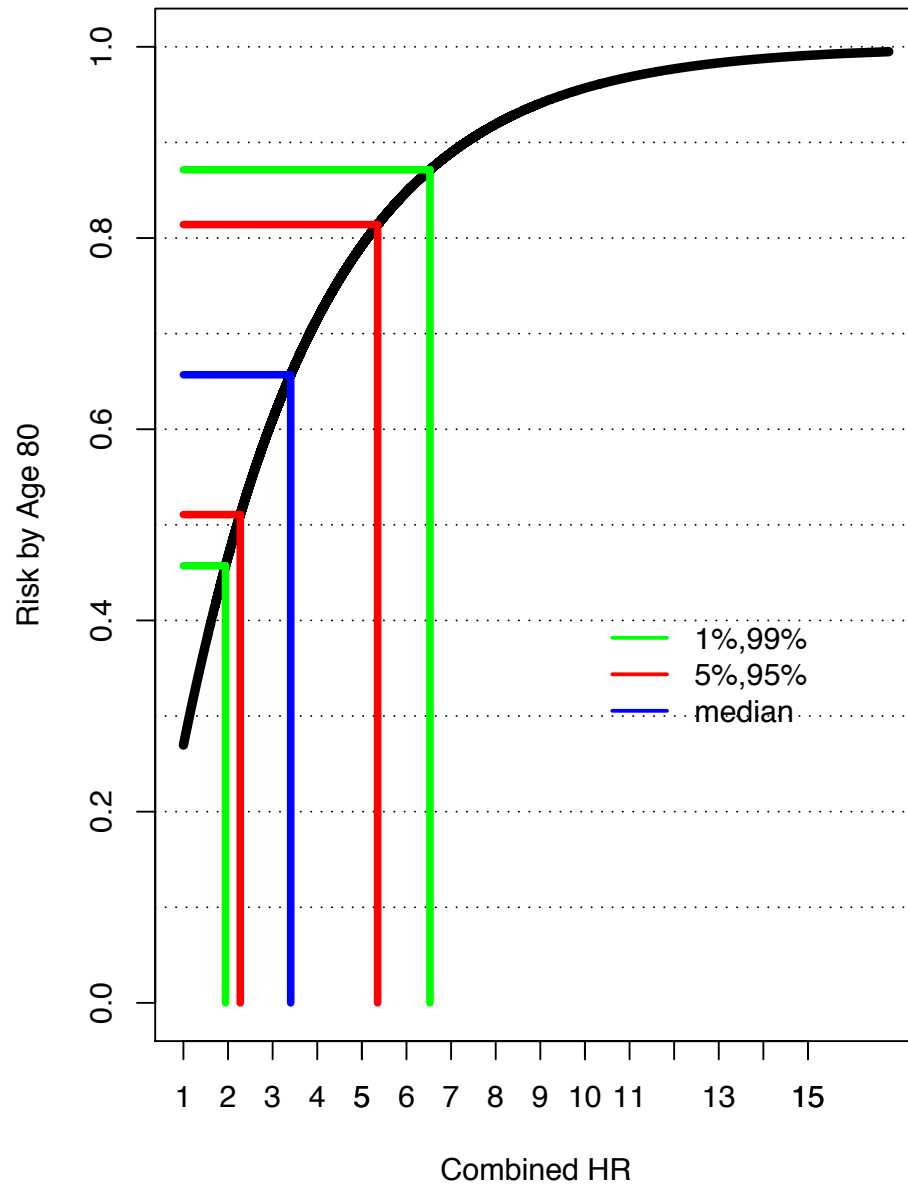

**B. Ovarian Cancer**

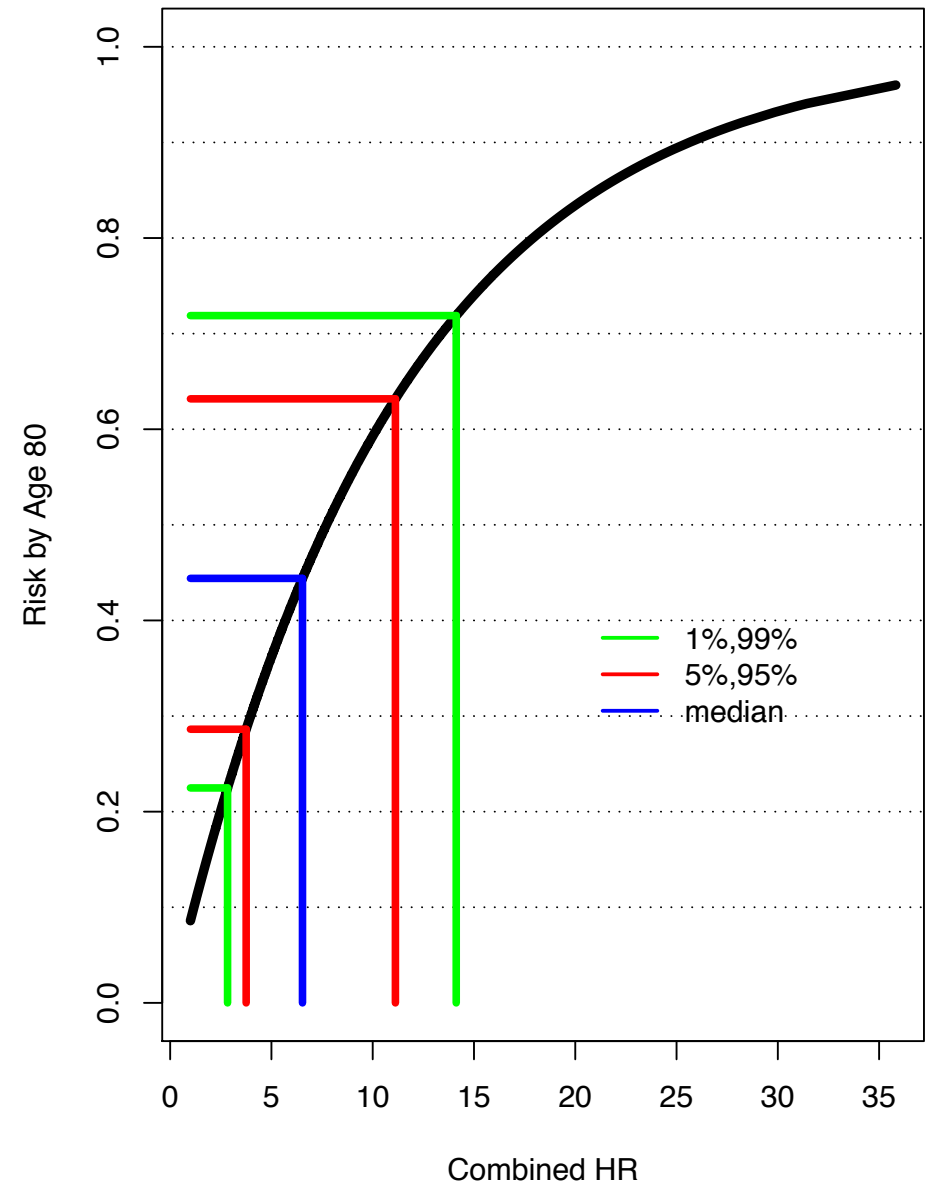

Supplement: Figure S10 — Combined Hazard Ratios (HR) for breast and ovarian cancer for BRCA1 mutation carriers. (A) HR for Breast Cancer based on 10 loci associated with breast cancer risk for BRCA1 mutation carriers. (B) Ovarian Cancer based on 7 loci associated with ovarian cancer risk for BRCA1 mutation carriers. All HRs computed relative to the lowest risk category. The Y-axes translate the combined HRs into absolute risks of developing breast or ovarian cancer by age 80. The absolute risks and HRs at different percentiles of the combined genotype distribution are also marked. The combined HRs were obtained under the assumption that the loci interact multiplicatively. (PDF) [file pgen.1003212.s010.pdf]

**A**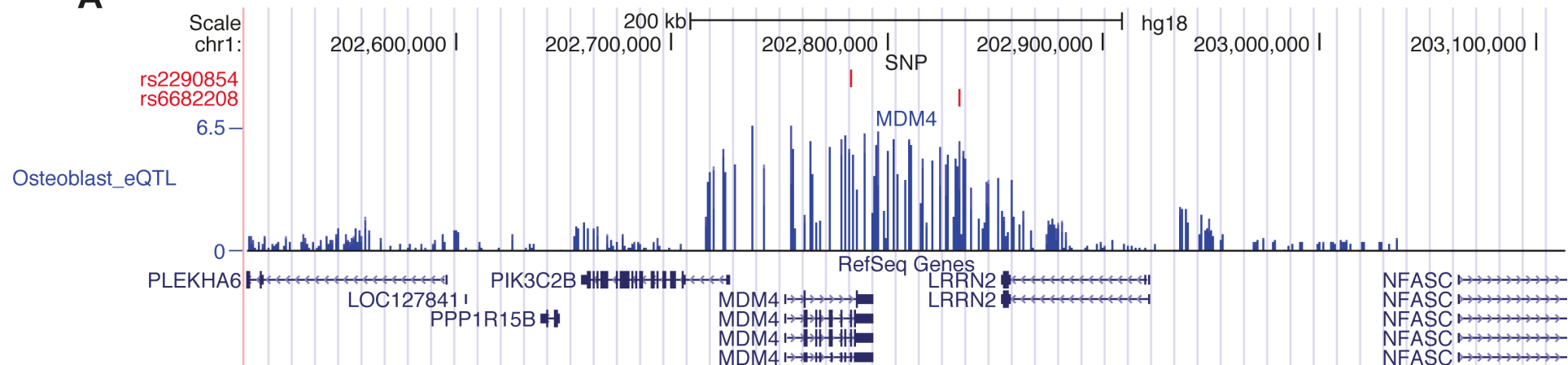**B**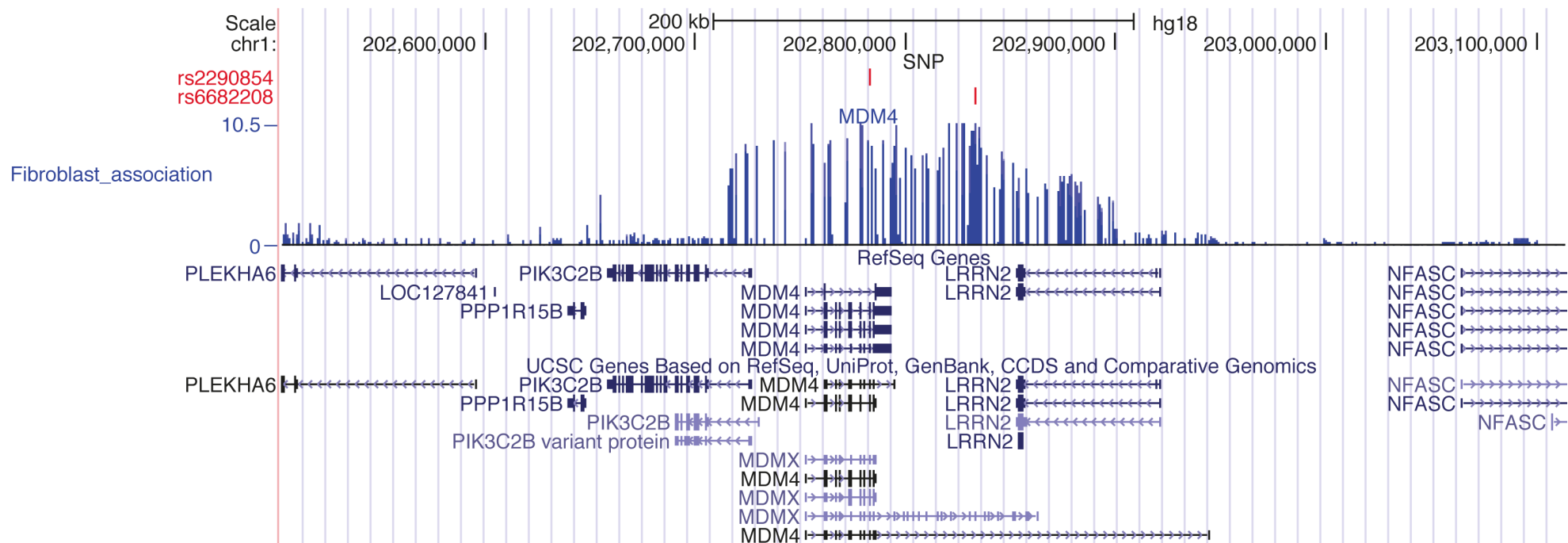

Supplementary Figure 11

Supplement: Figure S11 — Cis-eQTL and allelic expression (AE) analyses at MDM4 locus. A) Cis-eQTLs for SNPs at MDM4 locus using expression data from primary human osteoblasts (HOb). B) AE mapping for cis-regulatory variation in MDM4 locus using primary skin fibroblasts. Coordinates (hg18) for locus shown on top; blue tracks indicate the −log10(P value) of the association across all SNPs tested. The location of transcripts in this region is shown below. (PDF) [file pgen.1003212.s011.pdf]
