# Supplementary material for: Genome-Wide Association Study in BRCA1 Mutation Carriers Identifies Novel Loci Associated with Breast and Ovarian Cancer Risk
Source: PLoS Genet. 2013 Mar 27;9(3):e1003212. doi: 10.1371/journal.pgen.1003212 (PMC3609646; doi:10.1371/journal.pgen.1003212)
Supplement: Table S1 — Affected and unaffected BRCA1 mutation carriers by study country in the breast and ovarian cancer analysis used in SNP selection for the iCOGS array. (DOCX) [file pgen.1003212.s013.docx]

| **Table S1:** Affected and unaffected *BRCA1* mutation carriers by study country in the breast and ovarian cancer analysis used in SNP selection for the iCOGS array. | | | | |
| --- | --- | --- | --- | --- |
| **Country** | **Breast Cancer Analysis** | | **Ovarian Cancer Analysis** | |
|  | **Unaffected** | **Affected** | **Unaffected** | **Affected** |
| Australia | 91 | 114 | 156 | 49 |
| Canada | 113 | 90 | 150 | 53 |
| France | 24 | 89 | 86 | 27 |
| Germany | 95 | 173 | 189 | 79 |
| Italy | 93 | 127 | 123 | 97 |
| Poland | 102 | 73 | 164 | 11 |
| Spain | 53 | 64 | 79 | 38 |
| Sweden | 59 | 73 | 102 | 30 |
| Netherlands | 144 | 162 | 248 | 58 |
| UK | 240 | 211 | 337 | 114 |
| USA | 287 | 250 | 410 | 127 |
|  |  |  |  |  |
| Total | 1,301 | 1,426 | 2,044 | 683 |
