# Supplementary material for: Genome-Wide Association Study in BRCA1 Mutation Carriers Identifies Novel Loci Associated with Breast and Ovarian Cancer Risk
Source: PLoS Genet. 2013 Mar 27;9(3):e1003212. doi: 10.1371/journal.pgen.1003212 (PMC3609646; doi:10.1371/journal.pgen.1003212)
Supplement: Table S2 — Origin of BRCA1 samples by Country and Stage used in the current analysis. (DOCX) [file pgen.1003212.s014.docx]

| **Table S2**: Origin of *BRCA1* samples by Country and Stage used in the current analysis. | | | |
| --- | --- | --- | --- |
|  | **iCOGS** | |  |
|  | **Stage 1**^a^ | **Stage 2** | **Stage3** |
| **Country** |  |  |  |
|  |  |  |  |
| Australia | 179 | 483 | 130 |
| Austria |  | 332 | 4 |
| Canada | 194 | 353 | 16 |
| Czech Republic |  | 174 | 18 |
| Denmark |  | 459 | 248 |
| Finland |  | 97 | 2 |
| France | 74 | 642 | 235 |
| Germany | 236 | 376 | 518 |
| Greece |  | 100 | 72 |
| Hungary |  | 130 | 22 |
| Israel |  | 260 | 23 |
| Italy | 192 | 577 | 149 |
| Netherlands/Belgium | 285 | 1026 | 45 |
| Poland | 124 | 579 | 473 |
| Russia*/Latvia/Lithuania |  | 67 | 127 |
| South Africa |  | 61 | 1 |
| Spain | 94 | 240 | 136 |
| Sweden | 124 | 251 | 34 |
| UK/EIRE | 448 | 792 | 3 |
| USA | 437 | 2319 | 390 |
|  |  |  |  |
| Total | 2387 | 9318 | 2646 |
| *St Petersburg region. All Baltic region countries grouped together because individual study numbers were small.  ^a^: Samples genotyped on iCOGS that were also used for SNP selection purposes. | | | |
