# Supplementary material for: Genome-Wide Association Study in BRCA1 Mutation Carriers Identifies Novel Loci Associated with Breast and Ovarian Cancer Risk
Source: PLoS Genet. 2013 Mar 27;9(3):e1003212. doi: 10.1371/journal.pgen.1003212 (PMC3609646; doi:10.1371/journal.pgen.1003212)
Supplement: Table S3 — Sample and SNP quality control summary. (DOCX) [file pgen.1003212.s015.docx]

| **Table S3**: Sample and SNP quality control summary | | | | | |
| --- | --- | --- | --- | --- | --- |
| **Sample exclusion reason** | **N samples**  **excluded** | **N eligible**  **samples** | **SNP exclusion reason** | **N SNPs**  **excluded** | **N eligible**  **SNPs** |
| Genotyped samples |  | 13510 | SNPs on iCOGS |  | 211155 |
| Ineligible | 578 |  | Y chromosome | 79 |  |
| Incorrect gender | 50 |  | Call rate <95% | 4152 |  |
| Call rate <95% | 393 |  | Monomorphic | 3833 |  |
| Excess heterozygosity  (P<10^-6^) | 38 |  | Hardy-Weinberg  equilibrium failures (P<10^-7^) | 1827 |  |
| Non-European ancestry | 209 |  | High discordance rate  among duplicates | 22 |  |
| Duplicates (including cryptic) | 373 |  |  |  |  |
| Genotype inconsistencies  iCOGS vs previous genotyping | 164 |  |  |  |  |
|  |  |  |  |  |  |
| Total |  | 11705 |  |  | 201242 |
