# Supplementary material for: Genome-Wide Association Study in BRCA1 Mutation Carriers Identifies Novel Loci Associated with Breast and Ovarian Cancer Risk
Source: PLoS Genet. 2013 Mar 27;9(3):e1003212. doi: 10.1371/journal.pgen.1003212 (PMC3609646; doi:10.1371/journal.pgen.1003212)
Supplement: Table S4 — Associations with breast cancer risk for BRCA1 mutation carriers, for known breast cancer susceptibility variants. (DOCX) [file pgen.1003212.s016.docx]

| **Table S4:** Associations with breast cancer risk for *BRCA1* mutation carriers, for known breast cancer susceptibility variants. | | | | | | | | |
| --- | --- | --- | --- | --- | --- | --- | --- | --- |
|  | **Previous published association in *BRCA1*** | | | | **Association in present dataset** | | | |
| **Locus** | **SNP** | **all1/all2**  **(freq)** | **HR (95%CI)** | **P** | **Best tag SNP**  **(r^2^)** | **all1/all2**  **(freq)** | **HR (95%CI)** | **P** |
| *FGFR2* | rs2981582 | G/A  (0.40) | 1.03  (0.97-1.09) | 0.31 | rs2981582  (1.0) | G/A  (0.40) | 0.99  (0.94-1.03) | 0.57 |
| *MAP3K1* | rs889312 | A/C  (0.29) | 0.99  (0.93-1.05) | 0.63 | rs889312  (1.0) | A/C  (0.29) | 1.03  (0.97-1.08) | 0.32 |
| ***LSP1*** | rs3817198 | T/C  (0.32) | 1.05  (0.99-1.11) | 0.11 | **rs3817198**  **(1.0)** | **A/G**  **(0.32)** | **1.09**  **(1.04-1.14)** | **9.4×10^-4^** |
| 8q24 | rs13281615 | A/G  (.043) | 1.00  (0.95-1.05) | 0.93 | rs13281615  (1.0) | A/G  (0.42) | 1.01  (0.96-1.06) | 0.62 |
| *NEK10* | rs4973768 | C/T  (0.49) | 1.03  (0.98-1.08) | 0.26 | rs4973768  (1.0) | G/A  (0.49) | 1.02  (0.98-1.07) | 0.35 |
| COX11 | rs6504950 | G/A  (0.27) | 1.02  (0.96-1.08) | 0.59 | rs6504950  (1.0) | G/A  (0.27) | 0.99  (0.94-1.05) | 0.75 |
| 5p12 | rs10941679 | A/G  (0.25) | 0.96  (0.90-1.02) | 0.16 | rs10941679  (1.0) | A/G  (0.25) | 0.98  (0.92-1.03) | 0.38 |
| 1p11.2 | rs11249433 | T/C  (0.41) | 0.97  (0.92-1.02) | 0.20 | rs11249433  (1.0) | A/G  (0.41) | 1.00  (0.96-1.05) | 0.90 |
| ***RAD51L1*** | rs999737 / rs10483813 | C, T /T, A  (0.27) | 0.96  (0.90-1.03) | 0.27 | **rs999737**  **(1.0)** | **G/A**  **(0.22)** | **0.94**  **(0.89-0.99)** | **0.035** |
| *CDK2NA/B* | rs1011970 | G/T  (0.19) | 1.03  (0.96-1.09) | 0.45 | rs1011970  (1.0) | C/A  (0.17) | 1.02  (0.96-1.09) | 0.52 |
| *ZNF365* | rs10995190 | G/A  (0.16) | 0.99  (0.93-1.05) | 0.64 | rs10995190  (1.0) | G/A  (0.15) | 1.01  (0.95-1.08) | 0.81 |
| *ZMIZ1* | rs704010 | C/T  (0.37) | 1.02  (0.97-1.07) | 0.42 | rs704010  (1.0) | G/A  (0.37) | 0.99  (0.94-1.04) | 0.58 |
| 11q13 | rs614367 | C/T  (0.15) | 1.05  (0.98-1.12) | 0.15 | rs614367  (1.0) | G/A  (0.15) | 1.04  (0.98-1.11) | 0.23 |
| 12q24 | rs1292011 | A/G  (0.42) | 1.00  (0.94-1.06) | 0.99 | rs1292011  (1.0) | A/G  (0.42) | 0.99  (0.95-1.04) | 0.78 |
| 9q31.2 | rs865686 | T/G  (0.36) | 1.00  (0.96-1.05) | 0.85 | rs865686  (1.0) | A/C  (0.37) | 0.99  (0.94-1.04) | 0.67 |
| Freq= frequency of allele 2 in unaffected *BRCA1* carriers  HR= Per allele Hazard Ratio associated with allele 2  r^2^=r^2^ between published SNPs and the most significantly associated SNP in the present study  * SNP not in *BRCA1* GWAS SNP allocation on iCOGS chip | | | | | | | | |
