# Supplementary material for: Genome-Wide Association Study in BRCA1 Mutation Carriers Identifies Novel Loci Associated with Breast and Ovarian Cancer Risk
Source: PLoS Genet. 2013 Mar 27;9(3):e1003212. doi: 10.1371/journal.pgen.1003212 (PMC3609646; doi:10.1371/journal.pgen.1003212)
Supplement: Table S5 — Associations with BRCA1 breast or ovarian cancer risk for SNPs genotyped at stages 1, 2, and 3. (DOCX) [file pgen.1003212.s017.docx]

| **Table S5:** Associations with *BRCA1* breast or ovarian cancer risk for SNPs genotyped at stages 1, 2 and 3 | | | | | | | | |
| --- | --- | --- | --- | --- | --- | --- | --- | --- |
|  |  | **Stages 1 & 2** | | | **Stage3** | | | **All Stages** |
| **SNP** | **Chrom.** | **N** | **P-trend** | **Log(HR)** | **N** | **P-trend** | **Log(HR)** | **P-trend** |
| ***Breast Cancer Associations*** | | | | | | |  |  |
| rs2290854 | 1 | 11705 | 1.66×10^-5^ | 0.113 | 2645 | 1.28×10^-3^ | 0.176 | 1.39×10^-7^ |
| rs6682208 | 1 | 11705 | 5.44×10^-5^ | 0.104 | 2644 | 8.61×10^-4^ | 0.178 | 4.25×10^-7^ |
| rs11196174 | 10 | 11702 | 3.13×10^-5^ | 0.110 | 2644 | 5.71×10^-3^ | 0.153 | 7.48×10^-7^ |
| rs11196175 | 10 | 11682 | 3.90×10^-5^ | 0.109 | 2645 | 7.52×10^-3^ | 0.148 | 1.15×10^-6^ |
| rs765855 | 7 | 11702 | 1.17×10^-6^ | -0.125 | 2646 | 0.64 | -0.025 | 4.25×10^-6^ |
| rs2349485 | 7 | 11586 | 2.77×10^-6^ | -0.118 | 2646 | 0.68 | -0.022 | 9.74×10^-6^ |
| rs11616749 | 13 | 11705 | 2.89×10^-5^ | 0.124 | 2644 | 0.18 | 0.084 | 1.27×10^-5^ |
| rs17544947 | 17 | 11701 | 2.97×10^-5^ | -0.115 | 1039 | 0.21 | -0.116 | 1.31×10^-5^ |
| rs4716985 | 7 | 11696 | 2.34×10^-5^ | -0.108 | 2635 | 0.31 | -0.053 | 2.05×10^-5^ |
| rs1958654 | 14 | 11703 | 6.87×10^-6^ | 0.166 | 2645 | 0.90 | 0.009 | 3.24×10^-5^ |
| rs10252939 | 7 | 11705 | 4.88×10^-5^ | -0.106 | 2644 | 0.48 | -0.037 | 7.02×10^-5^ |
| rs10835161 | 11 | 11692 | 3.63×10^-5^ | -0.101 | 2644 | 0.86 | 0.009 | 2.22×10^-4^ |
|  |  |  |  |  |  |  |  |  |
| ***Ovarian Cancer Associations*** | | | | | | |  |  |
| rs1911544 | 2 | 11674 | 3.19×10^-5^ | 0.20 | 2642 | 0.905 | 0.012 | 1.58×10^-4^ |
| rs4691139 | 4 | 11705 | 1.11×10^-6^ | 0.18 | 2645 | 0.009 | 0.199 | 3.45×10^-8^ |
| rs17106475 | 10 | 11651 | 8.96×10^-6^ | -0.35 | 2646 | 0.753 | 0.050 | 1.11×10^-4^ |
| rs17631303 | 17 | 11624 | 3.04×10^-7^ | 0.24 | 2646 | 0.014 | 0.251 | 1.41×10^-8^ |
| rs183211 | 17 | 11705 | 5.67×10^-7^ | 0.22 | 2646 | 0.018 | 0.226 | 3.13×10^-8^ |
|  |  |  |  |  |  |  |  |  |
