# Supplementary material for: Genome-Wide Association Study in BRCA1 Mutation Carriers Identifies Novel Loci Associated with Breast and Ovarian Cancer Risk
Source: PLoS Genet. 2013 Mar 27;9(3):e1003212. doi: 10.1371/journal.pgen.1003212 (PMC3609646; doi:10.1371/journal.pgen.1003212)
Supplement: Table S6 — Analysis of breast cancer associations by BRCA1 mutation class. (DOCX) [file pgen.1003212.s018.docx]

| **Table S6**: Analysis of breast cancer associations by *BRCA1* mutation class. | | | | | | | | | | | | |
| --- | --- | --- | --- | --- | --- | --- | --- | --- | --- | --- | --- | --- |
|  |  |  |  |  |  | **Class 1** | | | **Class2** | | |  |
| **SNP** | **chrom** | **position** | **Locus** | **a1** | **a2** | **N** | **log(HR)** | **P** | **N** | **log(HR)** | **P** | **P-diff** |
| rs2290854 | 1 | 202782648 | *MDM4* | G | A | 9305 | 0.103 | 2.15×10^-4^ | 3981 | 0.185 | 3.58×10^-5^ | 0.12 |
| rs6682208 | 1 | 202832806 | *MDM4* | G | A | 9303 | 0.100 | 2.65×10^-4^ | 3982 | 0.168 | 1.64×10^-4^ | 0.19 |
| rs2349485 | 7 | 8517481 |  | A | C | 9240 | -0.105 | 1.13×10^-4^ | 3934 | -0.047 | 0.26 | 0.25 |
| rs765855 | 7 | 8519139 |  | G | A | 9302 | -0.117 | 2.39×10^-5^ | 3982 | -0.029 | 0.50 | 0.08 |
| rs4716985 | 7 | 155577229 |  | A | G | 9298 | -0.112 | 4.38×10^-5^ | 3969 | -0.079 | 0.066 | 0.52 |
| rs10252939 | 7 | 155587448 |  | A | G | 9303 | -0.110 | 7.21×10^-5^ | 3982 | -0.073 | 0.093 | 0.47 |
| rs11196174 | 10 | 114724086 | *TCF7L2* | A | G | 9301 | 0.079 | 5.09×10^-3^ | 3981 | 0.164 | 2.10×10^-4^ | 0.11 |
| rs11196175 | 10 | 114726604 | *TCF7L2* | A | G | 9284 | 0.077 | 6.46×10^-3^ | 3979 | 0.160 | 3.08×10^-4^ | 0.12 |
| rs10835161 | 11 | 27295265 |  | A | C | 9295 | -0.056 | 0.032 | 3977 | -0.070 | 0.092 | 0.79 |
| rs11616749 | 13 | 59373854 |  | G | A | 9304 | 0.109 | 5.94×10^-4^ | 3981 | 0.119 | 1.86×10^-2^ | 0.86 |
| rs1958654 | 14 | 53298267 |  | G | A | 9302 | 0.118 | 2.62×10^-3^ | 3982 | 0.133 | 3.59×10^-2^ | 0.85 |
| rs17544947 | 17 | 40274492 |  | A | C | 8508 | -0.079 | 9.65×10^-3^ | 3249 | -0.167 | 1.91×10^-3^ | 0.16 |
| a1,a2: allele1,2  log(HR): log Hazards ratio associated with allele2  P: p-value under the kinship adjusted score test | | | | | | | | | | | | |
