# Supplementary material for: Genome-Wide Association Study in BRCA1 Mutation Carriers Identifies Novel Loci Associated with Breast and Ovarian Cancer Risk
Source: PLoS Genet. 2013 Mar 27;9(3):e1003212. doi: 10.1371/journal.pgen.1003212 (PMC3609646; doi:10.1371/journal.pgen.1003212)
Supplement: Table S7 — Associations with Breast Cancer ER status in BRCA1 carriers for SNPs genotyped in stages 1–3. (DOCX) [file pgen.1003212.s019.docx]

| **Table S7**: Associations with Breast Cancer ER status in *BRCA1* carriers for SNPs genotyped in stages 1-3 | | | | | | | | | | | | | | |
| --- | --- | --- | --- | --- | --- | --- | --- | --- | --- | --- | --- | --- | --- | --- |
|  |  | **N (allele 2 freq)** | | | | **ER-positive** | | | | **ER-negative** | | | | **P-diff** |
|  |  |  | **Breast Cancer** | | | **HR** | **95%CI** | |  | **HR** | **95%CI** | |  |  |
| **SNP** | **chrom** | **Unaffected** | **ER+** | **ER-** | **Unknown** |  | **LB** | **UB** | **P** |  | **LB** | **UB** | **P** |  |
| rs2290854 | 1 | 7037  (0.31) | 634  (0.31) | 1953  (0.34) | 4727  (0.33) | 1.04 | 0.93 | 1.16 | 0.516 | 1.16 | 1.10 | 1.22 | 1.21×10^-7^ | 0.10 |
| rs6682208 | 1 | 7035  (0.32) | 634  (0.34) | 1953  (0.35) | 4727 (0.34) | 1.09 | 0.97 | 1.21 | 0.141 | 1.13 | 1.07 | 1.20 | 5.43×10^-6^ | 0.49 |
| rs10252939 | 7 | 7035  (0.32) | 634  (0.30) | 1953 (0.29) | 4727 (0.30) | 0.95 | 0.85 | 1.08 | 0.439 | 0.90 | 0.85 | 0.95 | 2.21×10^-4^ | 0.40 |
| rs2349485 | 7 | 6985  (0.37) | 629 (0.35) | 1935 (0.35) | 4683 (0.34) | 0.88 | 0.78 | 0.99 | 0.033 | 0.91 | 0.86 | 0.96 | 7.38×10^-4^ | 0.64 |
| rs4716985 | 7 | 7028  (0.33) | 634 (0.30) | 1952  (0.29) | 4717 (0.30) | 0.94 | 0.83 | 1.06 | 0.318 | 0.89 | 0.84 | 0.95 | 1.71×10^-4^ | 0.51 |
| rs765855 | 7 | 7036  (0.34) | 634  (0.32) | 1952  (0.32) | 4726  (0.31) | 0.88 | 0.78 | 0.99 | 0.030 | 0.90 | 0.85 | 0.96 | 3.47×10^-4^ | 0.68 |
| rs11196174 | 10 | 7035  (0.28) | 634  (0.29) | 1951  (0.31) | 4726 (0.31) | 1.09 | 0.97 | 1.22 | 0.147 | 1.14 | 1.07 | 1.20 | 9.55×10^-6^ | 0.52 |
| rs11196175 | 10 | 7026  (0.28) | 633 (0.29) | 1947 (0.30) | 4721 (0.31) | 1.08 | 0.97 | 1.22 | 0.171 | 1.14 | 1.07 | 1.20 | 1.21×10^-5^ | 0.50 |
| rs10835161 | 11 | 7030  (0.43) | 634  (0.41) | 1949 (0.41) | 4723 (0.41) | 0.94 | 0.84 | 1.04 | 0.230 | 0.92 | 0.87 | 0.97 | 1.83×10^-3^ | 0.77 |
| rs11616749 | 13 | 7036  (0.20) | 634  (0.21) | 1953 (0.21) | 4726 (0.22) | 1.14 | 1.01 | 1.29 | 0.041 | 1.12 | 1.05 | 1.19 | 5.15×10^-4^ | 0.81 |
| rs1958654 | 14 | 7035  (0.12) | 634 (0.12) | 1952 (0.14) | 4727 (0.13) | 1.02 | 0.86 | 1.21 | 0.824 | 1.19 | 1.10 | 1.28 | 9.96×10^-6^ | 0.13 |
| rs17544947 | 17 | 6260  (0.29) | 585 (0.28) | 1848  (0.27) | 4050 (0.26) | 0.95 | 0.84 | 1.07 | 0.387 | 0.88 | 0.82 | 0.93 | 5.67×10^-5^ | 0.27 |
|  |  |  |  |  |  |  |  |  |  |  |  |  |  |  |
