# Supplementary material for: Genome-Wide Association Study in BRCA1 Mutation Carriers Identifies Novel Loci Associated with Breast and Ovarian Cancer Risk
Source: PLoS Genet. 2013 Mar 27;9(3):e1003212. doi: 10.1371/journal.pgen.1003212 (PMC3609646; doi:10.1371/journal.pgen.1003212)
Supplement: Table S8 — Imputed SNPs at the novel 17q21 region with P-values less than the most significant genotyped SNP (rs169201). (DOCX) [file pgen.1003212.s020.docx]

| **Table S8**: Imputed SNPs at the novel 17q21 region with P-values less than the most significant genotyped SNP (rs169201) | | | | | | | | | | |
| --- | --- | --- | --- | --- | --- | --- | --- | --- | --- | --- |
|  |  |  |  | **BRCA1** | | | **BRCA2** | | | **BRCA1 and BRCA2** |
| **SNP** | **Position** | **A1** | **A2** | **Freq A.1** | **Rsq** | **P** | **Freq all.1** | **Rsq** | **P** | **P** |
| 17-44034340 | 44034340 | T | C | 0.166 | 0.77 | 1.25×10^-7^ | 0.178 | 0.777 | 3.18×10^-6^ | 3.52×10^-12^ |
| 17-44034486 | 44034486 | G | A | 0.177 | 0.791 | 1.08×10^-7^ | 0.19 | 0.799 | 5.84×10^-6^ | 4.82×10^-12^ |
| 17-43681414 | 43681414 | G | C | 0.199 | 0.655 | 3.35×10^-7^ | 0.204 | 0.644 | 3.57×10^-6^ | 1.26×10^-11^ |
| 17-44034194 | 44034194 | T | C | 0.15 | 0.725 | 1.16×10^-7^ | 0.157 | 0.724 | 1.89×10^-5^ | 1.38×10^-11^ |
| 17-44034209 | 44034209 | C | T | 0.15 | 0.726 | 1.20×10^-7^ | 0.158 | 0.724 | 1.95×10^-5^ | 1.47×10^-11^ |
| 17-43691152 | 43691152 | T | A | 0.169 | 0.799 | 2.90×10^-7^ | 0.177 | 0.79 | 7.84×10^-6^ | 1.89×10^-11^ |
| 17-43702778 | 43702778 | A | G | 0.114 | 0.61 | 6.05×10^-8^ | 0.114 | 0.58 | 8.70×10^-5^ | 2.67×10^-11^ |
| 17-43702792 | 43702792 | A | G | 0.113 | 0.611 | 6.67×10^-8^ | 0.113 | 0.58 | 8.26×10^-5^ | 2.84×10^-11^ |
| 17-44034575 | 44034575 | C | T | 0.159 | 0.757 | 6.68×10^-7^ | 0.172 | 0.763 | 4.58×10^-6^ | 2.96×10^-11^ |
| 17-43681222 | 43681222 | T | C | 0.221 | 0.709 | 6.61×10^-7^ | 0.224 | 0.696 | 4.46×10^-6^ | 3.19×10^-11^ |
| 17-43664545 | 43664545 | A | C | 0.184 | 0.78 | 5.85×10^-7^ | 0.196 | 0.785 | 6.12×10^-6^ | 3.25×10^-11^ |
| 17-43682098 | 43682098 | C | A | 0.161 | 0.768 | 1.07×10^-6^ | 0.176 | 0.781 | 3.12×10^-6^ | 3.58×10^-11^ |
| 17-43682129 | 43682129 | T | C | 0.162 | 0.77 | 1.35×10^-6^ | 0.177 | 0.783 | 4.15×10^-6^ | 5.75×10^-11^ |
| 17-44773783 | 44773783 | G | A | 0.798 | 0.949 | 3.42×10^-7^ | 0.783 | 0.948 | 2.73×10^-5^ | 5.86×10^-11^ |
| A1: Allele 1  A2:Allele 2  Rsq: Imputation accuracy r^2^ | | | | | | | | | | |
