# Supplementary material for: Genome-Wide Association Study in BRCA1 Mutation Carriers Identifies Novel Loci Associated with Breast and Ovarian Cancer Risk
Source: PLoS Genet. 2013 Mar 27;9(3):e1003212. doi: 10.1371/journal.pgen.1003212 (PMC3609646; doi:10.1371/journal.pgen.1003212)
Supplement: Table S9 — SNPs associated (P<1×10^−5^) with local expression and Allelic Imbalance. (DOCX) [file pgen.1003212.s021.docx]

| **Table S9:** SNPs associated (P<1×10^-5^) with local expression and Allelic Imbalance | | | | | | | | | |
| --- | --- | --- | --- | --- | --- | --- | --- | --- | --- |
| **SNP^a^** | **Chr** | **Position^b^** | **Cell type^c^** | **Gene** | **Probe** | **P-value** | **Best functional SNP** | **r^2,d^** | **Reference** |
| **Chromosome 1** | | |  |  |  |  |  |  |  |
| rs7556371 | 1 | 202723959 | B cell | MDM4 | NM_002393.2 | 8.40E-08 | rs11240751 | 0.92 | Fairfax et al, 2012^e^ |
| rs7556371 | 1 | 202723959 | Monocytes | MDM4 | NM_002393.2 | 7.55E-11 | rs2290854 | 0.85 | Fairfax et al, 2012^e^ |
| rs11240751 | 1 | 202728673 | B cell | MDM4 | NM_002393.2 | 9.65E-09 | rs11240751 | 1 | Fairfax et al, 2012^e^ |
| rs11240751 | 1 | 202728673 | Monocytes | MDM4 | NM_002393.2 | 1.10E-10 | rs2290854 | 0.92 | Fairfax et al, 2012^e^ |
| rs2290854 | 1 | 202782648 | B cell | MDM4 | NM_002393.2 | 7.38E-08 | rs11240751 | 0.92 | Fairfax et al, 2012^e^ |
| rs2290854 | 1 | 202782648 | Monocytes | MDM4 | NM_002393.2 | 4.80E-11 | rs2290854 | 1 | Fairfax et al, 2012^e^ |
| rs2290854 | 1 | 202782648 | Osteoblasts | MDM4 | NM_002393.1 | 5.99E-06 | rs10900594 | 0.96 | Unpublished^f^ |
| rs2290854 | 1 | 202782648 | Fibroblast (AE) | MDM4 | uc001hbc.2 | 1.70E-09 | rs12130686 | 0.82 | Unpublished^g^ |
| rs4245739 | 1 | 202785465 | B cell | MDM4 | NM_002393.2 | 3.88E-06 | rs11240751 | N/A | Fairfax et al, 2012^e^ |
| rs4245739 | 1 | 202785465 | Monocytes | MDM4 | NM_002393.2 | 1.76E-10 | rs2290854 | 0.87 | Fairfax et al, 2012^e^ |
| rs10900597 | 1 | 202789112 | B cell | MDM4 | NM_002393.2 | 6.07E-07 | rs11240751 | 0.96 | Fairfax et al, 2012^e^ |
| rs10900597 | 1 | 202789112 | Monocytes | MDM4 | NM_002393.2 | 1.89E-09 | rs2290854 | 0.96 | Fairfax et al, 2012^e^ |
| rs6682208 | 1 | 202832806 | B cell | MDM4 | NM_002393.2 | 4.99E-08 | rs11240751 | 0.80 | Fairfax et al, 2012^e^ |
| rs6682208 | 1 | 202832806 | Monocytes | MDM4 | NM_002393.2 | 1.63E-07 | rs2290854 | 0.81 | Fairfax et al, 2012^e^ |
| rs6682208 | 1 | 202832806 | Fibroblast (AE) | MDM4 | uc001hbc.2 | 1.60E-10 | rs12130686 | 0.96 | Unpublished^g^ |
| rs10900601 | 1 | 202838673 | B cell | MDM4 | NM_002393.2 | 5.33E-05 | rs11240751 | 0.66 | Fairfax et al, 2012^e^ |
| rs10900601 | 1 | 202838673 | Monocytes | MDM4 | NM_002393.2 | 6.22E-07 | rs2290854 | 0.67 | Fairfax et al, 2012^e^ |
| **Chromosome 17** | | |  |  |  |  |  |  |  |
| rs12947718 | 17 | 40848884 | B cell | MGC57346 | XM_377476.4 | 1.95E-22 | rs11012 | 1 | Fairfax et al, 2012^e^ |
| rs12947718 | 17 | 40848884 | Monocytes | MGC57346 | XM_377476.4 | 6.44E-54 | rs17631676 | 1 | Fairfax et al, 2012^e^ |
| rs11012 | 17 | 40869224 | B cell | MGC57346 | XM_377476.4 | 1.69E-23 | rs11012 | 1 | Fairfax et al, 2012^e^ |
| rs11012 | 17 | 40869224 | Monocytes | MGC57346 | XM_377476.4 | 1.73E-54 | rs17631676 | 1 | Fairfax et al, 2012^e^ |
| rs17631676 | 17 | 40905309 | B cell | MGC57346 | XM_377476.4 | 2.20E-23 | rs11012 | 1 | Fairfax et al, 2012^e^ |
| rs17631676 | 17 | 40905309 | Monocytes | MGC57346 | XM_377476.4 | 1.80E-55 | rs17631676 | 1 | Fairfax et al, 2012^e^ |
| rs12942666 | 17 | 40855622 | Osteoblasts | C17orf69 | NM_152466.1 | 2.29E-10 | rs17762073 | 0.66 | Unpublished^f^ |
| rs17631303 | 17 | 40872185 | Osteoblasts | C17orf69 | NM_152466.1 | 4.03E-12 | rs17762073 | 0.66 | Unpublished^f^ |
| rs17631303 | 17 | 40872185 | Lymphoblastoid (AE) | ARHGAP27 | NM_199282 | 8.50E-12 | rs12947718 | 1 | Unpublished^g^ |
| rs418891 | 17 | 41049321 | Osteoblasts | C17orf69 | NM_152466.1 | 1.30E-15 | rs17762073 | 1 | Unpublished^f^ |
| rs1105569 | 17 | 41149171 | Osteoblasts | C17orf69 | NM_152466.1 | 3.85E-14 | rs17762073 | 0.73 | Unpublished^f^ |
| rs1880752 | 17 | 41162047 | Osteoblasts | C17orf69 | NM_152466.1 | 7.08E-14 | rs17762073 | 1 | Unpublished^f^ |
| rs17762954 | 17 | 41255567 | Osteoblasts | C17orf69 | NM_152466.1 | 6.51E-13 | rs17762073 | 1 | Unpublished^f^ |
| rs17762954 | 17 | 41255567 | Lymphoblastoid (AE) | KANSL1 | uc002ikc.1 | 4.30E-09 | rs878887 | 0.83 | Unpublished^g^ |
| rs1876829 | 17 | 41267224 | Osteoblasts | C17orf69 | NM_152466.1 | 8.74E-15 | rs17762073 | 0.80 | Unpublished^f^ |
| rs1876829 | 17 | 41267224 | Lymphoblastoid (AE) | KANSL1 | uc002ikc.1 | 1.30E-10 | rs878887 | 0.89 | Unpublished^g^ |
| rs17691610 | 17 | 41326456 | Osteoblasts | C17orf69 | NM_152466.1 | 5.18E-14 | rs17762073 | 1 | Unpublished^f^ |
| rs17691610 | 17 | 41326456 | Lymphoblastoid (AE) | KANSL1 | uc002ikc.1 | 1.90E-07 | rs878887 | 0.79 | Unpublished^g^ |
| rs17563965 | 17 | 41346747 | Osteoblasts | C17orf69 | NM_152466.1 | 3.85E-14 | rs17762073 | 1 | Unpublished^f^ |
| rs17563965 | 17 | 41346747 | Lymphoblastoid (AE) | KANSL1 | uc002ikc.1 | 1.90E-07 | rs878887 | 0.79 | Unpublished^g^ |
| rs17650335 | 17 | 41368172 | Osteoblasts | C17orf69 | NM_152466.1 | 1.33E-13 | rs17762073 | 1 | Unpublished^f^ |
| rs17650335 | 17 | 41368172 | Lymphoblastoid (AE) | KANSL1 | uc002ikc.1 | 1.90E-07 | rs878887 | 0.79 | Unpublished^g^ |
| rs17650381 | 17 | 41368853 | Osteoblasts | C17orf69 | NM_152466.1 | 3.70E-14 | rs17762073 | 1.0 | Unpublished^f^ |
| rs17650381 | 17 | 41368853 | Lymphoblastoid (AE) | KANSL1 | uc002ikc.1 | 1.90E-07 | rs878887 | 0.79 | Unpublished^g^ |
| rs17652121 | 17 | 41429810 | Osteoblasts | C17orf69 | NM_152466.1 | 3.41E-12 | rs17762073 | 0.57 | Unpublished^f^ |
| rs1991556 | 17 | 41439239 | Osteoblasts | C17orf69 | NM_152466.1 | 4.28E-12 | rs17762073 | 0.90 | Unpublished^f^ |
| rs17652961 | 17 | 41464202 | Osteoblasts | C17orf69 | NM_152466.1 | 3.07E-14 | rs17762073 | 1.0 | Unpublished^f^ |
| rs17652961 | 17 | 41464202 | Lymphoblastoid (AE) | KANSL1 | uc002ikc.1 | 1.90E-07 | rs878887 | 0.79 | Unpublished^g^ |
| rs17660464 | 17 | 41533806 | Osteoblasts | C17orf69 | NM_152466.1 | 1.13E-15 | rs17762073 | 1.0 | Unpublished^f^ |
| rs17660464 | 17 | 41533806 | Lymphoblastoid (AE) | KANSL1 | uc002ikc.1 | 1.90E-07 | rs878887 | 0.79 | Unpublished^g^ |
| rs17577094 | 17 | 41543275 | Osteoblasts | C17orf69 | NM_152466.1 | 3.89E-14 | rs17762073 | 1.0 | Unpublished^f^ |
| rs17577094 | 17 | 41543275 | Lymphoblastoid (AE) | KANSL1 | uc002ikc.1 | 1.90E-07 | rs878887 | 0.79 | Unpublished^g^ |
| rs183211 | 17 | 42143493 | Lymphoblastoid (AE) | WNT3 | NM_030753 | 1.60E-11 | rs183211 | 1 | Unpublished^g^ |
| a: All SNPs at Chr 1 are associated with breast cancer risk for *BRCA1* (P<10^-4^ in stage 1 and 2 samples), and SNPs at Chr 17 associated with ovarian cancer risk for *BRCA1/2* combined (P<10^-8^) | | | | | | | | |  |
| b: genome build 36.3 | | | | | | | | |  |
| c: Allelic expression analysis in fibroblasts and lymphoblastoid cell lines is indicated by AE | | | | | | | | |  |
| d: Correlation between the risk associated SNP and the best SNP associated with the transcript | | | | | | | | |  |
| e: Fairfax, B.P. et al. Genetics of gene expression in primary immune cells identifies cell type-specific master regulators and roles of HLA alleles. Nat Genet(2012) | | | | | | | | | |
| f: Unpublished data based on experiments described in: Grundberg,E. et al. Global analysis of the impact of environmental perturbation on cis-regulation of gene expression. PLoS Genet 7, e1001279 (2011).  g: Unpublished data based on experiments described in: Ge,B. et al. Global patterns of cis variation in human cells revealed by high-density allelic expression analysis. *Nat Genet* 41, 1216-1222 (2009). | | | | | | | | | |
