# Supplementary material for: Genome-Wide Association Study in BRCA1 Mutation Carriers Identifies Novel Loci Associated with Breast and Ovarian Cancer Risk
Source: PLoS Genet. 2013 Mar 27;9(3):e1003212. doi: 10.1371/journal.pgen.1003212 (PMC3609646; doi:10.1371/journal.pgen.1003212)
Supplement: Text S1 — Supplementary Methods. (DOCX) [file pgen.1003212.s022.docx]

**Text S1**

Supporting Information includes Supplementary Methods, nine Supplementary Tables and twelve Supplementary Figures.

**Supplementary Methods**

**Genotype calling**

Genotypes for samples genotyped on the iCOGS array were called using Illumina’s GenCall algorithm. Initial calling used a cluster file generated using 270 Hapmap2 samples. To generate the final calls, we first selected a subset of 3,018 individuals, including samples from each genotyping centers, each participating consortium, and each major ethnicity. Only plates with a consistent high call rate in the initial calling were used. We also included 380 samples of European, African and Asian ancestry genotyped by the HapMap and 1000 Genomes Project, and 160 samples that were known positive controls for rare variants on iCOGS. This subset was used to generate a cluster file that was applied to call the genotypes for the remaining samples. We also investigated the Illumnus[1] and GenoSNP[2] algorithms. The calls were >99% concordant for 91% of the SNPs on the array. However, manual inspection of a sample of discrepant SNPs indicated that the GenCall calls were superior. Therefore, only the GenCall genotypes were used.

**Genotype concordance**

Of the 13,510 samples, 578 did not fulfil eligibility criteria based on phenotypic data and were excluded. Samples found to be discordant between iCOGS and other genotyping efforts were excluded from the analysis. 7,559 samples had also been genotyped for 80 SNPs in common with the iCOGS array as part of a rapid replication of Stage 1 results[3]. The genotype concordance among these samples was 99.7%. In addition, 9,362 samples had been previously genotyped for ≥14 SNPs that were also on the iCOGS array, as part of previous CIMBA genotyping efforts (genotype concordance: 98.1%). Samples found to be discordant between iCOGS and other genotyping efforts were excluded from the analysis.

**Associations by tumor subtype**

The analysis of associations by breast cancer ER-status was carried out by an extension of the retrospective likelihood approach to model the simultaneous effect of each SNP on more than one tumor subtype[4]. Briefly, this involved modeling the conditional likelihood of the observed SNP genotypes and tumor subtypes, given the disease phenotypes. The HRs for each tumor subtype were estimated simultaneously. To maximise available information, genotyped mutation carriers with missing ER-status were included in the analysis, and their ER-status was assumed to be missing at random[4].

**Cis-eQTL and allelic expression analyses**

For SNPs at chromosomes 1 and 17 showing association with breast or ovarian cancer risk, allelic expression cis-associations were mapped using the llumina Human1M-duo BeadChip for lymphoblastoid cell lines from Caucasians (CEU population) (n=53)[5] and Illumina Human 1M Omni-quad for primary skin fibroblasts derived from Caucasian donors (n = 62)[6] (Pastinen et al unpublished data). Genotyping was carried out in parallel and direct HapMap genotype calls (CEU) or imputed (MACH 1.0) HapMap genotypes for fibroblasts were used for mapping. The unrelated fibroblast panel consisted of 31 parent-offspring trios, where the genotypes of offspring were used to allow for accurate phasing. Mapping of each allelic expression trait was carried out by first normalizing allelic expression ratios at each SNP using a polynomial method[7] and then calculating averaged phased allelic expression scores across annotated transcripts, followed by correlation of these scores to local (transcript +/-250kb) SNP genotypes in population panels as described earlier[5]. Cis-eQTLs for SNPs of these two loci were also analysed using expression data from primary human osteoblasts (HOb). Association analyses were carried out using the Illumina HumRef-8v2 BeadChips for expression traits and Illumina HapMap 550k Duo chip for genotypes[7]. Cis-regulatory effects were tested using SNPs mapping the candidate SNP +/- 250kb. For the osteoblast samples, we similarly imputed the genotypes (MACH 1.0) for HapMap SNPs to provide a larger density of SNPs to test. Cis-associations were tested by applying a linear regression model implemented in the PLINK software using the imputed genotypes. Similarly, the Caucasian fibroblast cohort, used also in allelic expression mapping (above), was subjected to cis-eQTL mapping applying Illumina HumanRef-8 v3.0 for expression traits (Pastinen et al., unpublished data).

**References**

1. Teo YY, Inouye M, Small KS, Gwilliam R, Deloukas P et al. (2007) A genotype calling algorithm for the Illumina BeadArray platform. Bioinformatics 23: 2741-2746. btm443 [pii];10.1093/bioinformatics/btm443 [doi].

2. Giannoulatou E, Yau C, Colella S, Ragoussis J, Holmes CC (2008) GenoSNP: a variational Bayes within-sample SNP genotyping algorithm that does not require a reference population. Bioinformatics 24: 2209-2214. btn386 [pii];10.1093/bioinformatics/btn386 [doi].

3. Antoniou AC, Wang X, Fredericksen ZS, McGuffog L, Tarrell R et al. (2010) A locus on 19p13 modifies risk of breast cancer in BRCA1 mutation carriers and is associated with hormone receptor-negative breast cancer in the general population. Nat Genet 42: 885-892. ng.669 [pii];10.1038/ng.669 [doi].

4. Mulligan AM, Couch FJ, Barrowdale D, Domchek SM, Eccles D et al. (2011) Common breast cancer susceptibility alleles are associated with tumor subtypes in BRCA1 and BRCA2 mutation carriers: results from the Consortium of Investigators of Modifiers of BRCA1/2. Breast Cancer Res 13: R110. bcr3052 [pii];10.1186/bcr3052 [doi].

5. Ge B, Pokholok DK, Kwan T, Grundberg E, Morcos L et al. (2009) Global patterns of cis variation in human cells revealed by high-density allelic expression analysis. Nat Genet 41: 1216-1222. ng.473 [pii];10.1038/ng.473 [doi].

6. Morcos L, Ge B, Koka V, Lam KC, Pokholok DK et al. (2011) Genome-wide assessment of imprinted expression in human cells. Genome Biol 12: R25. gb-2011-12-3-r25 [pii];10.1186/gb-2011-12-3-r25 [doi].

7. Grundberg E, Adoue V, Kwan T, Ge B, Duan QL et al. (2011) Global analysis of the impact of environmental perturbation on cis-regulation of gene expression. PLoS Genet 7: e1001279. 10.1371/journal.pgen.1001279 [doi].
