# Supplementary material for: Genome-Wide Association Study in BRCA1 Mutation Carriers Identifies Novel Loci Associated with Breast and Ovarian Cancer Risk
Source: PLoS Genet. 2013 Mar 27;9(3):e1003212. doi: 10.1371/journal.pgen.1003212 (PMC3609646; doi:10.1371/journal.pgen.1003212)
Supplement: Figure S12 — Cis-eQTL and allelic expression (AE) analyses at chr17q21.31 locus. (Upper panel) Cis-eQTLs for SNPs at c17orf69 locus using expression data from primary human osteoblasts. Allelic expression mapping for cis-regulatory variation in KANSL1 (middle panel) and WNT3 loci (lower panel) using a CEU population panel of lymphoblastoid cells. Coordinates (hg18) for loci are shown on top; blue tracks indicate the −log10(P value) of the association across all SNPs tested. The location of transcripts in these regions are shown. (PDF) [file pgen.1003212.s012.pdf]

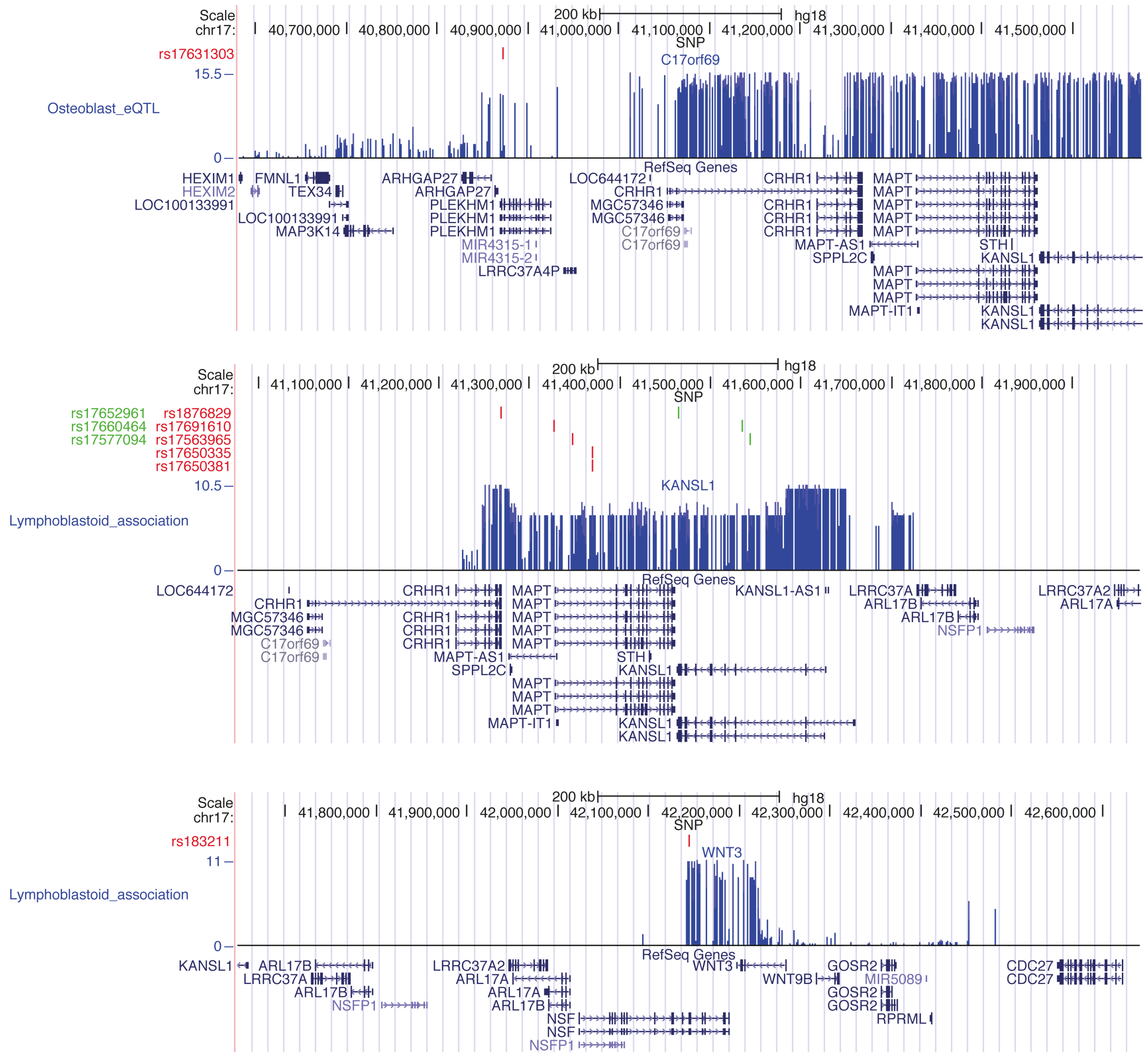

Supplementary Figure 12
